# Mechanistic Insights and Synthetic Explorations of the Photoredox-Catalyzed Activation of Halophosphines

**DOI:** 10.1021/acs.inorgchem.3c01946

**Published:** 2023-10-19

**Authors:** Anna I. Arkhypchuk, Thuan T. Tran, Rima Charaf, Leif Hammarström, Sascha Ott

**Affiliations:** Department of Chemistry—Ångström, Laboratory Uppsala University, P.O. Box 523, 751 20 Uppsala, Sweden

## Abstract

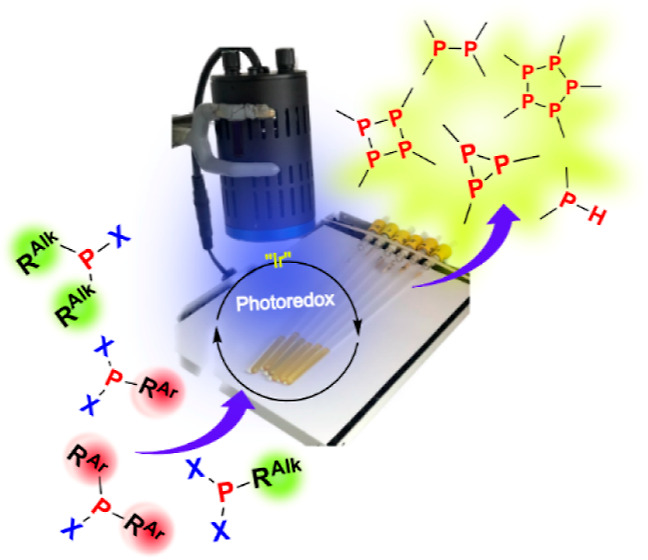

The light-driven activation of halophosphines R_2_PX (R = alkyl- or aryl, X = Cl, Br) by an Ir^III^-based photocatalyst is described. It is shown that initially formed secondary phosphines R_2_PH react readily with the remaining R_2_PX in a parent–child reaction to form diphosphines R_2_P–PR_2_. Aryl-containing diphosphines can be further reduced to secondary phosphines R^Ar^_2_PH under identical photoredox conditions. Dihalophosphines RPX_2_ are also activated by the photoredox protocol, giving rise to unusual 3-, 4-, and 5-membered cyclophosphines. Transient absorption studies show that the excited state of the Ir photocatalyst is reductively quenched by the DIPEA (*N*,*N*-di-iso-propylethylamine) electron donor. Electron transfer to R_2_PX is however unexpectedly slow and cannot compete with recombination with the oxidized donor DIPEA^•+^. As DIPEA is not a perfectly reversible donor, a small proportion of the total Ir^II^ population escapes recombination, providing the reductant for the observed transformations.

## Abstract 2

Visible light-induced activation of halo- and dihalophosphines by an Ir-based photocatalyst gives access to a number of phosphorus-based synthons, the reactivity of which is investigated. Among the obtained products are diphosphines, cyclic oligophosphines, as well as secondary phosphines that can be obtained in synthetically useful yields.

The advent of photoredox-catalyzed organic transformations has had a transformative impact on organic chemistry over the past decade.^1−3^ It has reignited the interest in radical chemistry by providing sustainable access to radical species that previously required the involvement of hazardous radical initiators (e.g., AIBN, BEt_3_)^4^ or other toxic agents (e.g., Bu_3_SnH).^5^ A prominent entry into photoredox-catalyzed organic transformations is provided by substrates that carry halogen-heteroatoms. The photoredox-mediated reductive dehalogenation of carbon–halogen bonds gives access to carbon-based radicals,^6−8^ the reactivity of which can be explored in a variety of reactions (Figure 1A). Given the impact that photoredox catalysis has had on organic synthesis, it is surprising that it is highly underexplored in phospha-organic chemistry. Only recently, the Wolf group has reported the activation of P_4_ by aryl radicals that were produced photocatalytically from haloarenes.^9−11^ Photoredox activation of P–Cl bonds and explorations of the subsequent chemistry are, to the best of our knowledge, entirely unexplored. If viable, such photoredox routes (Figure 1B) could give rise to synthetically versatile synthons such as secondary phosphines and complex organophosphorus compounds (OPCs), providing highly sought-after alternatives to traditional nonbenign procedures.

**Figure 1:**
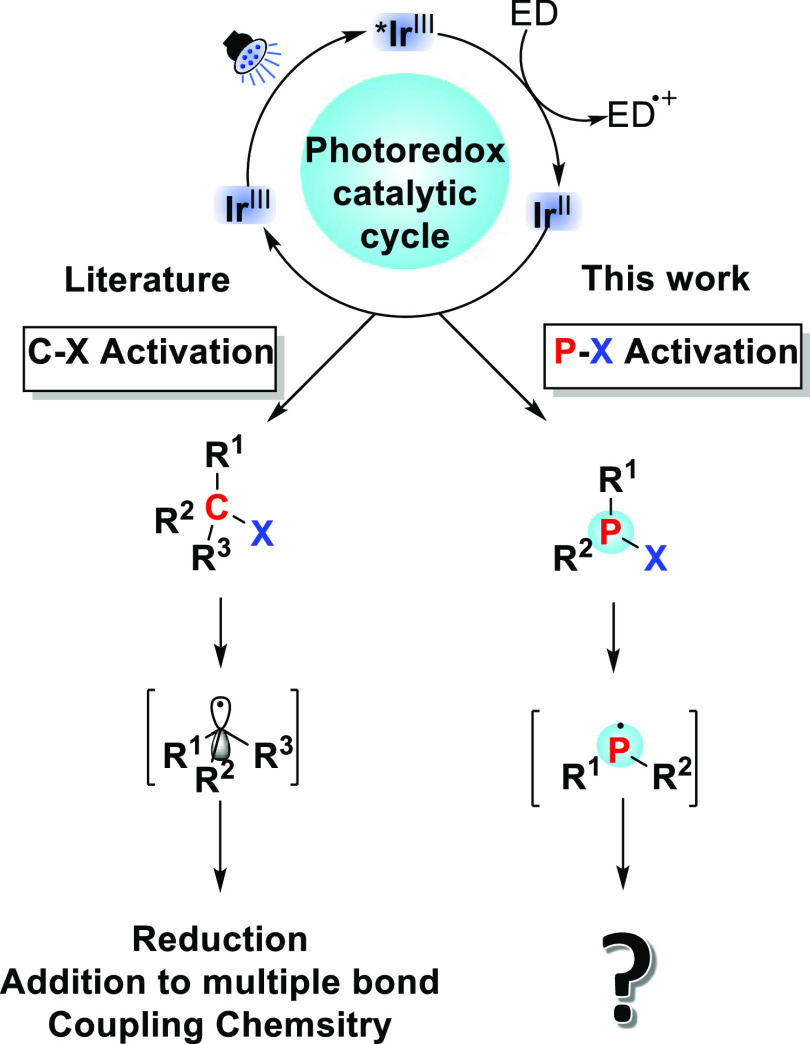
Photoredox-mediated activation of carbon–halogen bonds (A) and this work: novel photoredox-mediated activation of phosphorus–halogen bonds (B). An electron donor (ED) is used to transform *Ir^III^ to Ir^II^. This ED may be DIPEA.

Herein, we report the first study of the light-driven activation of halophosphines R_2_PX and dihalophosphines RPX_2_ (R = alkyl- or aryl, X = Cl, Br) catalyzed by a commercially available Ir^III^-based photocatalyst. In the case of R_2_PX, diphosphines R_2_P–PR_2_ are produced from reduced R_2_PH and parent R_2_PX as the first identifiable reaction product in almost all cases. Aryl-containing diphosphines R^Ar^_4_P_2_ can be further reduced under prolonged reaction times to form secondary phosphines R^Ar^_2_PH in very good overall yields. The photoreduction of dihalophosphines RPX_2_ is more complex as two P–X bonds can be broken, offering synthetic routes to higher P_*n*_ products. Under optimized reactions, synthetically highly challenging 3-, 4-, and 5-membered cyclophosphines are obtained in respectable yields. The mechanism of the photoredox reaction was scrutinized by emission quenching and transient absorption techniques, which showed that the excited state of the catalyst is reductively quenched by the DIPEA (*N*,*N*-di-iso-propylethylamine) electron donor. Electron transfer from the thereby produced Ir^II^ state to the halophosphine is however unexpectedly slow and cannot compete with recombination between the oxidized donor (DIPEA^•+^). Instead, the chemistry is driven by a small proportion of Ir^II^ that escapes recombination.

Solutions of the halophosphine starting materials in CH_3_CN were irradiated with a Kessil Tuna Blue lamp in the presence of DIPEA and catalytic amounts (0.1 mol %) of Ir[dFFppy]_2_-(4,4′-dCF_3_bpy)PF_6_. The progress of all reactions was monitored by quantitative ^31^P NMR spectroscopy, using tris(4-fluorophenyl)phosphine as an internal standard. All yields are determined by quantitative ^31^P NMR spectroscopy of crude mixtures, if not specifically stated otherwise (see Supporting Information for details.)

## Alkyl-Substituted Chlorophosphines

**Scheme 1:**
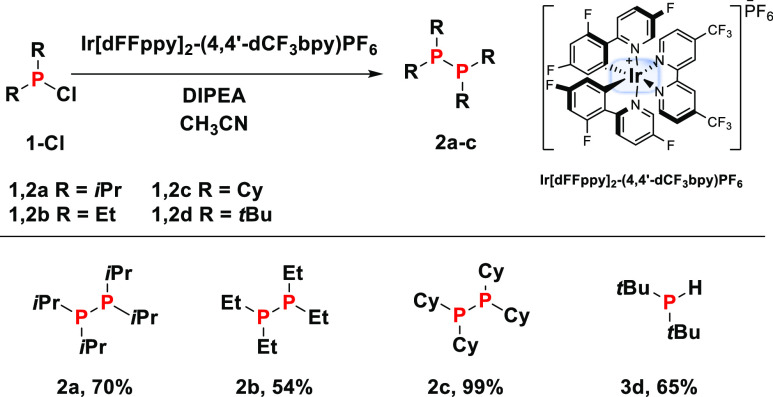
Iridium-Catalyzed Photoredox Reaction of Dialkylchlorophosphines R^Alk^_2_PCl **(1a–d)-Cl** Reaction was performed in Young-type NMR tubes on 20 μL of substrate, 100 μL of DIPEA, and ca. 0.1 mol % Ir[dFFppy]_2_-(4,4′-dCF_3_bpy)PF_6_ in 0.5 mL of CH_3_CN. Tris(4-fluorophenyl)phosphine is used as an internal standard to determine reaction yields.

Chlorophosphines with two aliphatic substituents (R^Alk^_2_PCl) have a considerably more negative reduction potential than their diaryl analogues (R^Ar^_2_PCl, Figure S2) and were thus investigated separately. Exposed to the standard conditions described above, (*i*-Pr)_2_PCl (**1a-Cl**) was completely consumed over 36 h, during which a product emerged that was characterized by a singlet ^31^P NMR resonance at δ = −10.0 ppm. The compound was identified as tetra-iso-propyldiphosphine **2a** by comparison with the literature data^12^ and was afforded in a 70% yield (Scheme 1). Et_2_PCl and Cy_2_PCl react in the same fashion, giving the analogous diphosphine products **2b** and **2c** in 54 and 99% yields, respectively (Scheme 1).^12,13^ In contrast, phosphine **1d-Cl** with the more bulky *tert*-butyl substituents resulted in the formation of the secondary phosphine **3d**(14) in a 65% yield, and no diphosphine **2d** could be observed. In addition, the large steric bulk of the *tert*-butyl groups gives rise to vastly prolonged reaction times (104 days in THF). More important, though, is that the reaction produces the secondary phosphine **3d**, which suggests that the diphosphines **2a–c** may be formed in a child–parent reaction between the initially formed R^Alk^_2_PH and unreacted R^Alk^_2_PCl.

## Aryl-Substituted Chlorophosphines

With this hypothesis in mind, diarylchlorophosphines R^Ar^_2_PCl were exposed to identical photoredox conditions. After 12 h of irradiation, Ph_2_PCl (**1e-Cl**) had converted to a mixture of tetraphenyldiphosphine **2e** and the secondary phosphine Ph_2_PH (**3e**) in roughly equal amounts (Table 1, entry 1).^12,15^ Careful reaction monitoring revealed that diphosphine **2e** is formed at early stages of the experiment (65% after 45 min) but is subsequently consumed under the formation of Ph_2_PH **3e** that is obtained in a 74% yield after 24 h. To unambiguously prove the origin of **3e**, an independently prepared sample of **2e**(16) was treated under the photoredox conditions, and **3e** was obtained in a 93% yield. Control experiments that omitted either Ir[dFFppy]_2_-(4,4′-dCF_3_bpy)PF_6_, visible light, or DIPEA did not lead to any meaningful amounts of **3e** (<0.3% in the absence of photocatalyst). This observation confirmed that both the production of (Ph_2_P)_2_, as well as its subsequent reduction to Ph_2_PH are two separate processes, both of which are photoredox-catalyzed. The reduction of the diphosphines is exclusive to those that carry aryl substituents and is not observed for the alkyl analogues **2a–c**. The observed reactivity difference is consistent with the reduction potentials of the diphosphines that are considerably more negative for those that carry alkyl groups, compared to those with aryl substituents (Figure S3). While Lewis acid or Rh-mediated P–P bond reductions to regenerate secondary phosphines have been reported previously,^17,18^ the present work provides the first photocatalytic route for this reaction. As such, it presents opportunities for the development of future reactions that require low steady-state concentrations of secondary phosphines.

**Table 1:**
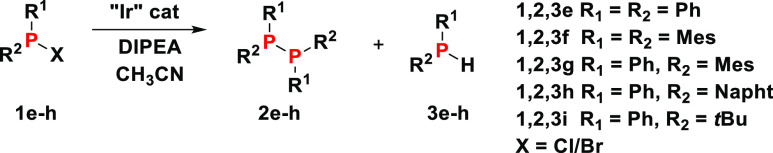
Photochemical Reduction of the Aryl Substituted Monohalophosphines

entry	starting material	irradiation time^d^	yield **2**, (%)	yield **3**, (%)
1	**1e-Cl**^a^	15 min	48	0
		45 min	65	0
		12 h	36	26
		24 h	0	74
2	**1e-Br**^b^	15 min	65	0
		45 min	68	0
		12 h	0	75
		24 h	0	82
3	**1f-X**^c^	17 h	56	0
4	**1f-Br**^b^	3 h	66	0
5	**1g-X**^c^	4 h	55	0
		7 days	0	63
6	**1g-Br**^b^	4 h	71	0
		7 days	0	76
7	**1h-X**^c^	45 min	91	0
		6 days	19	76
8	**1h-Br**^b^	45 min	92	0
		6 days	14	83
9	**1i-Cl**^a^	1 day	74	0

a Starting material is commercially available.

b Starting material was prepared from the corresponding chloride via TMSBr-assisted halogen exchange.

c Prepared as a mixture of bromo- and chlorodiarylphosphines.

d Reaction was performed in Young-type NMR tubes on 20 μL of substrate, 100 μL of DIPEA, and ca. 0.1 mol % Ir[dFFppy]_2_-(4,4′-dCF_3_bpy)PF_6_ in 0.5 mL of CH_3_CN. Tris(4-fluorophenyl)phosphine is used as an internal standard to determine reaction yields.

## Effects of Halide and Solvents for Reaction Optimization

**Figure 2:**
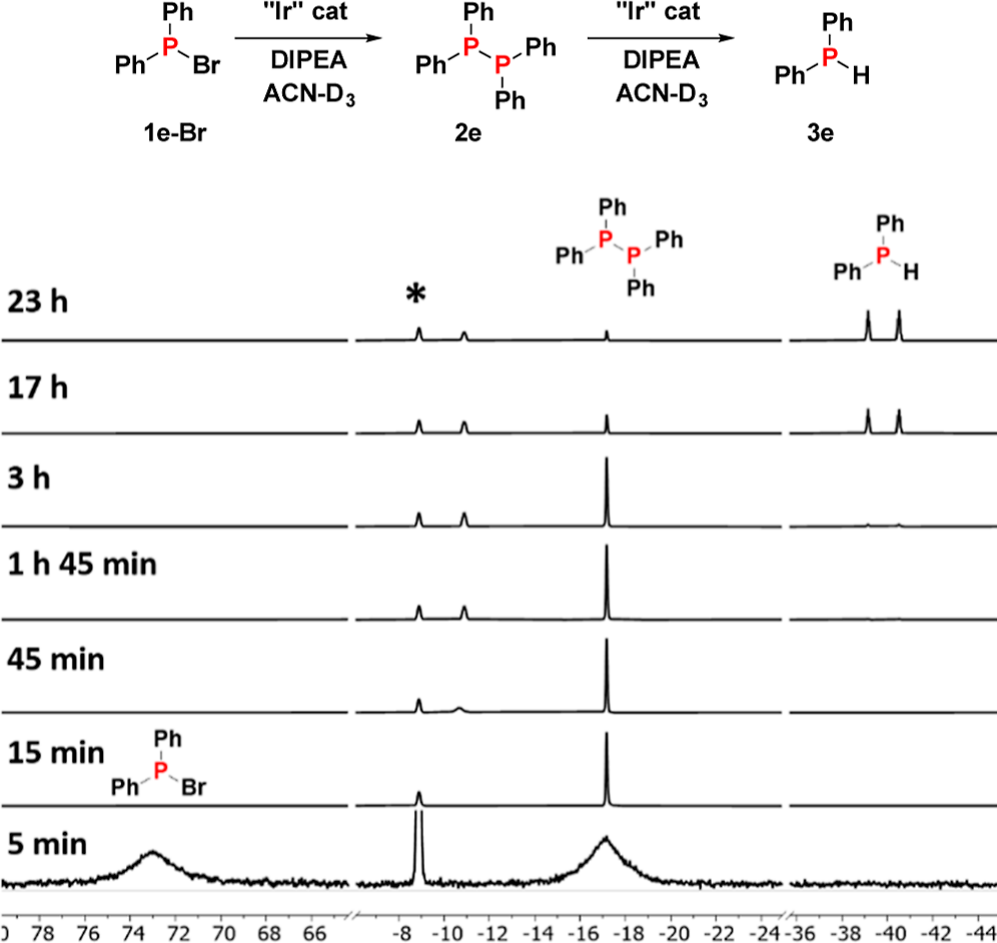
^31^P NMR monitoring of the iridium-catalyzed photoreaction of **1e-Br**. Tris(4-fluorophenyl)phosphine (δ = −8.9 ppm) was used as an internal standard to determine reaction yields and is marked with an asterisk. Spectrum at *t* = 5 min is enlarged in order to show the broad peaks of starting phosphine **1e-Br** (δ = 73.1 ppm) and diphosphine **2e** (δ = −17.0 ppm). The signal at −13 ppm arises from a currently unknown intermediate that gradually decreases during the course of the reaction.

Next, differences in reactivity between chloro- and bromo-substituted halophosphines were examined. As shown in Figure 2, **1e-Br** is fully consumed already after 15 min under the formation of diphosphine **2e**. The latter is converted to the secondary phosphine **3e** on the time scale of hours. Thus, both diphenylhalophosphines (**1e-Cl** and **1e-Br**) give rise to the same diphosphine **2e**, with the rate of this transformation being somewhat accelerated in the case of **1e-Br** (Table 1). The shorter reaction time in the case of **1e-Br** is accompanied by higher yields for the formation of **2e** (Table 1). The enhanced rate is consistent with Ph_2_PBr being easier to reduce and bromide generally being a better leaving group. Competition experiments of mixtures of chloro- and bromodiarylphosphines further confirmed the described reactivity pattern (Table 1). Such mixtures of PhNapthPX **1h-X**,^19^ PhMesPX **1g-X**,^20^ and Mes_2_PX **1f-X**(21) are obtained from the addition of solutions of PhPCl_2_ (or PCl_3_ in case of **1f**) to the corresponding bromo-Grignard reagent. Illumination of the mixtures of R^Ar^_2_PCl and R^Ar^_2_PBr starting materials results in the consumption of R^Ar^_2_PBr prior to that of the corresponding R^Ar^_2_PCl analogue (Figures S23 and S28). Ultimately, both halophosphines converge to the same diphosphines **2g–h** which are then reduced further to the secondary phosphines **3g–h** (Table 1). While diphosphine **2g** is obtained from **1g-Br** in a crude yield of 71% after 4 h, oxidative decomposition compromises the isolated yield to 48% at a preparative scale (see Supporting Information for details). Reaction times and yields also show a dependence on the steric bulk of the substrates, with bulkier halophosphines generally being less reactive.

As electron-transfer reactions and cage escape yields often vary with solvent polarity,^22^ the diphosphine formation and its subsequent reduction were studied in solvents of different polarities, namely CH_3_CN, THF, toluene, and pentane (Tables 2 and S1). Diphosphine formation proceeds in all cases, however, at very different rates. A general observation is that the reaction in CH_3_CN occurs on a time scale of hours, while that in THF or toluene is slowed down to days (Table 2). The most extreme cases are reactions in pentane, which can require weeks for completion (Table S1, entry 9). Reflecting the difference in reaction rate for diphosphine formation, the subsequent reductions to the secondary phosphines are also influenced by solvent polarity. While diphosphine **2e** is relatively stable in THF and toluene (Table S1, entries 7 and 8), it can be reduced to secondary phosphine in CH_3_CN (Table S1, entry 6).

**Table 2:**
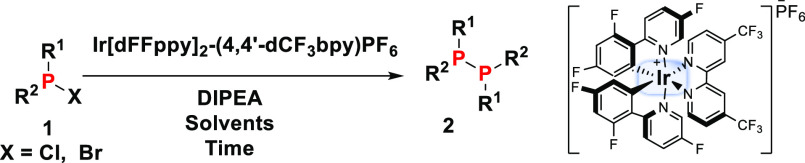

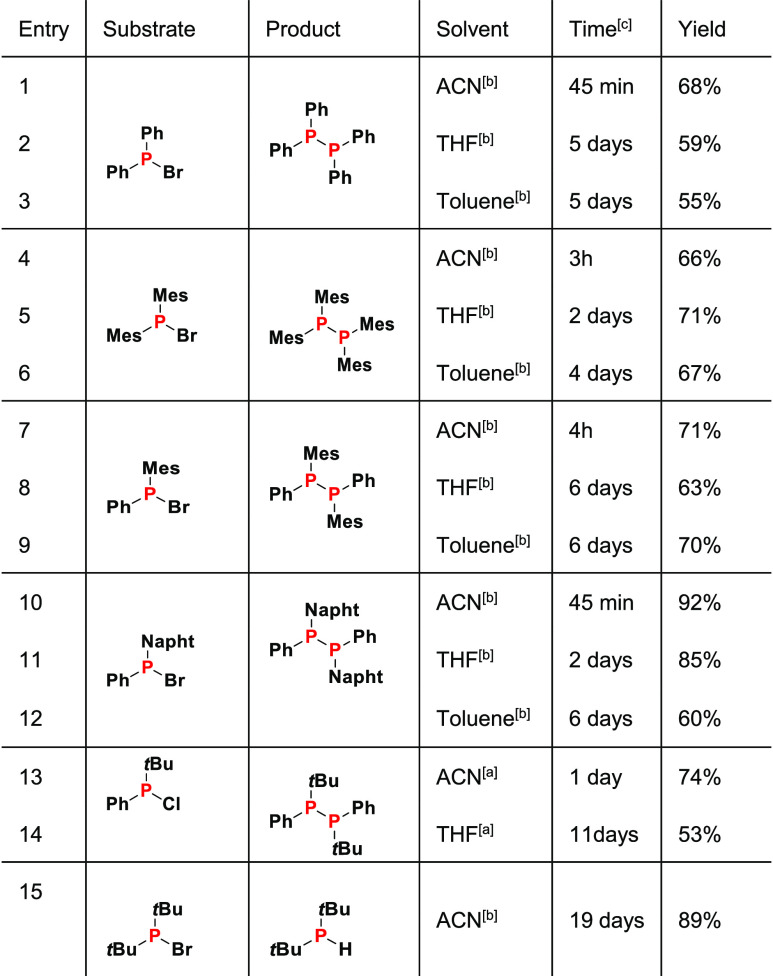
Reaction Scope and Optimized Conditions for the Photochemical Reduction of Monohalophosphines **1e–i**

a Starting material is commercially available, X = Cl.

b Starting material was prepared from corresponding chloride via TMSBr-assisted halogen exchange, X = Br.

c Reaction was performed in Young-type NMR tubes on 20 μL of substrate, 100 μL of DIPEA, and ca. 0.1 mol % Ir[dFFppy]_2_-(4,4′-dCF_3_bpy)PF_6_ was used in 0.5 mL of corresponding solvents. Reaction time corresponds to the irradiation time. Tris(4-fluorophenyl)phosphine was used as an internal standard to determine reaction yields.

Taken together, the informed choice of solvent and irradiation time allows for the selective formation of either diphosphine or secondary phosphine. As a rule of thumb, the secondary phosphine is obtained fastest and in the highest yields from the bromophosphines in CH_3_CN, while diphosphines can be obtained most reliably in less polar solvents such as THF or pentane but at the cost of longer reaction times (Table 2). Dialkylhalophosphines (R^Alk^_2_PX) generally react slower than their aryl analogues (R^Ar^_2_PX) and cannot be reduced further under the explored conditions. If needed, reaction times can presumably be accelerated by raising the catalyst loading to more than the 0.1 mol % that was used throughout this study. Diphosphines with cyclohexyl (**2c**) and mesityl (**2f**) substituents that are notoriously difficult to synthesize by other methods due to high steric hindrance can be prepared in high yields by the presented protocol.^23^

Asymmetrically disubstituted halophosphines result in the formation of a diastereomeric mixture of diphosphines, namely, as the meso- and rac-compounds. The mixture can, for example, be observed in 1,2-di-*tert*-butyl-1,2-diphenyldiphosphine, the ^31^P NMR spectrum of which features two singlets at δ_rac_ = 4.6 and δ_meso_ = −2.2 ppm, with the meso isomer being preferred in a relative ratio of 1:5.6.^24,25^ Similar results were also obtained for 1,2-dimesityl-1,2-diphenyldiphosphine,^26^ as well as 1,2-di(naphthalen-2-yl)-1,2-diphenyldiphosphine.

## Reaction Mechanism

Mechanistic details behind the observed photoredox catalysis were investigated by emission quenching and transient absorption spectroscopic studies on three different halophosphine substrates, Cy_2_PCl (**1c-Cl**), Ph_2_PCl (**1e-Cl**), and Ph_2_PBr (**1e-Br**). As expected, the emission of the *Ir^III^ excited state is reductively quenched by the DIPEA donor to produce Ir^II^ (Figure S5). Stern-Volmer plots reveal that this quenching is diffusion-controlled with rate constants *k*_q_ of 1.1 × 10^10^ M^–1^ s^–1^ (Figure S4). Inclusion of halophosphines to the quenching experiment has very little effect, and the observed quenching rate constants remain largely unaffected. This is consistent with control experiments that show that the halophosphines quench the *Ir^III^ excited state about 2 orders of magnitude slower (Figures S6, S8 and S10).

**Figure 3:**
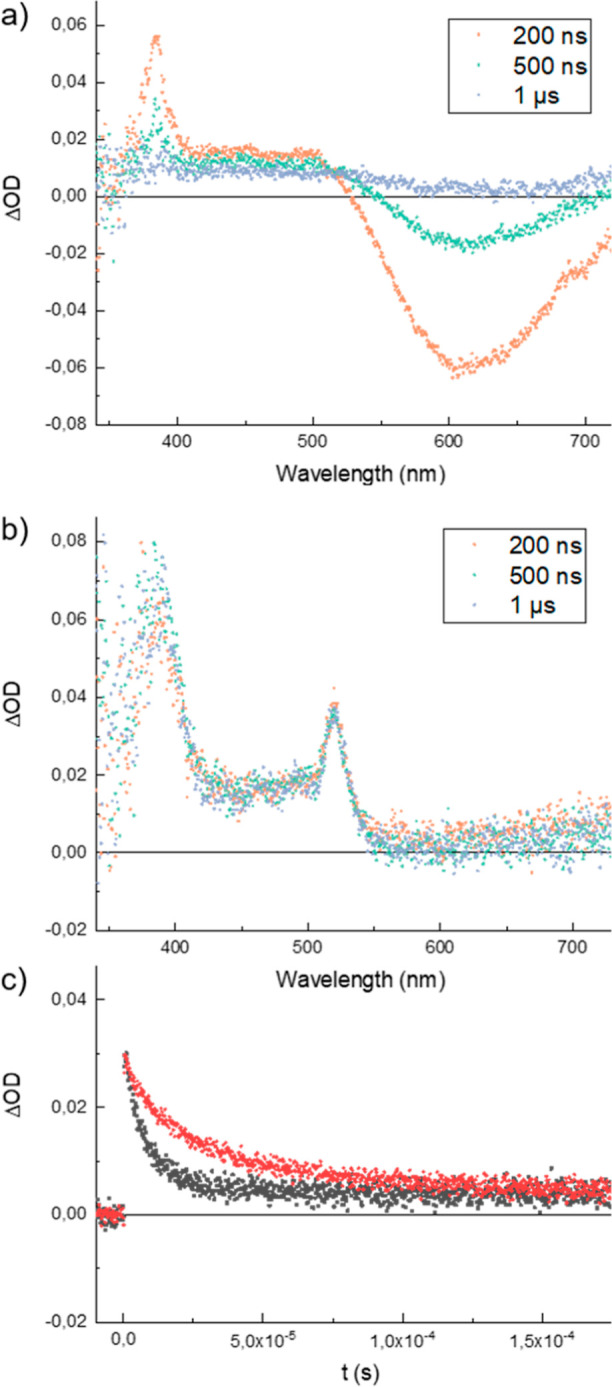
(a) Transient absorption difference spectra of Ir[dFFppy]_2_-(4,4′-dCF_3_bpy)PF_6_ (100 μM in CH_3_CN) at different time delays after laser excitation at 410 nm. (b) Same experiment as under (a) but in the presence of DIPEA electron donor. (c) Kinetic traces of the absorption decay at 520 nm, probing the recombination of the photoproduced Ir^II^ state with the oxidized DIPEA donor in the absence (black) and presence (red) of Ph_2_PBr phosphine substrate.

The fate of the photocatalyst after light excitation was further investigated by transient absorption spectroscopy. In the absence of any DIPEA donor (Figure 3a), the excited state is characterized by an absorption in the near-UV. The negative absorption centered around 610 nm is due to excited-state emission (photoluminescence). The catalyst returns to its ground state ca. 1 μs after excitation. In the presence of DIPEA (Figure 3b), the spectra were markedly different. No signal that arises from excited-state emission can be observed, and a new absorption emerges at 520 nm instead. This is consistent with the proposed reductive quenching of the *Ir^III^ excited state, and the formation of the reduced Ir^II^ state, the build-up and consumption of which can thus conveniently be probed at 520 nm. The kinetic traces at 520 nm shows that the photogenerated Ir^II^ recombines with oxidized donor, DIPEA^+^, in the absence of halophosphines (Figure 3c, black trace) on a 10 s of μs time scale. It also becomes evident that the recombination is not quantitative, as the trace does not revert to zero. Confirming this observation, illumination experiments on the second time scale show that Ir^II^ is built-up under illumination. This is consistent with earlier reports that DIPEA is a partially irreversible donor that decomposes on the time scale of the Ir^II^ state.^27^

Adding a halophosphine substrate to the experiment gives rise to some rather unexpected observations. The expectation would be that the substrate reacts with the Ir^II^ reductant toward product formation. Such a reaction would imply a faster recovery of the Ir^III^ in the transient absorption experiments, i.e., catalyst turnover. Much to our surprise, the kinetic trace at 520 nm clearly shows that the lifetime of the Ir^II^ state is actually prolonged by the addition of the substrate (Figure 3, red trace). This observation has multiple implications. First, the electron transfer from Ir^II^ to the substrate is slower than recombination with the DIPEA^+^. Second, as the recombination is decelerated in the presence of the halophosphine, a reversible interaction between DIPEA^+^ and the halophosphine must be invoked. As to the nature of this interaction, we can only speculate at present, but it has previously been shown that the oxidized DIPEA^+^ is in equilibrium with a deprotonated form, resulting in an acid/base equilibrium that may be disturbed by the halophosphine. On the microsecond time scale, the system levels off at the same concentration of residual Ir^II^ as in the absence of halophosphine. All of these observations point toward the interpretation that all productive chemistry that is observed on the synthetic time scale of minutes and longer actually originates from Ir^II^ that avoids recombination due to partial decomposition of the DIPEA^+^.

Thus, the light-driven buildup of Ir^II^ was followed on an even longer time scale to gain correlations with experimental observations. As can be seen in Figure S12, the buildup of Ir^II^ occurs even in the presence of Cy_2_PCl (**1c**), which is one of the least reactive substrates. Electron transfer between Ir^II^ and **1c** is thus slower than the buildup of the Ir^II^. In contrast, the addition of **1h-Br** to solutions of Ir^II^ leads to the disappearance of the absorption at 520 nm, consistent with electron transfer. This reactivity pattern parallels the experimental findings that diarylhalophosphines (R^Ar^_2_PX) react faster than dialkylchlorophosphines (R^Alk^_2_PX). In the absence of substrate, the accumulation of Ir^II^ on these time scales is more efficient in CH_3_CN compared to toluene (Figure S13), consistent with the experimental finding that reactions in CH_3_CN are faster.

**Figure 4:**
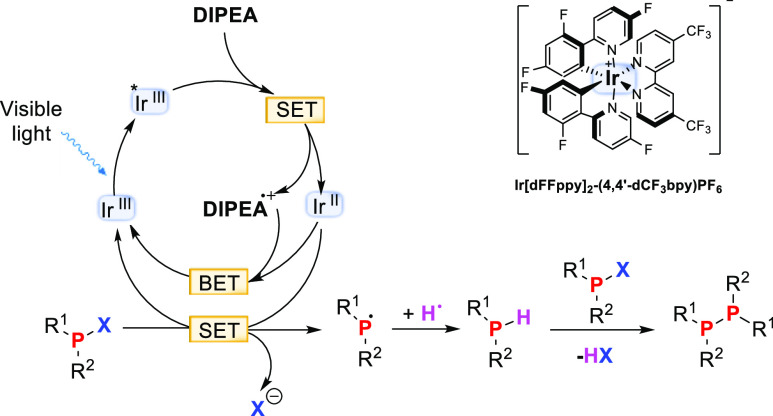
Proposed mechanism for the photoredox-catalyzed activation of halophosphines. SET refers to single electron transfer, and BET refers to back electron transfer.

Based on the above results, a more realistic mechanism for the light-driven activation of halophosphines can be proposed (Figure 4). Excitation and reductive quenching of the photocatalyst result in the formation of the Ir^II^ reductant, the large majority of which, however, recombines with oxidized DIPEA^•+^ (Figure 4, BET). As DIPEA is, however, not a perfectly reversible donor, a small amount of Ir^II^ is much longer lived, and it is this residual Ir^II^ population that drives the synthetic transformation. Electron transfer to the halophosphine is presumably followed by the extrusion of a halogen anion to produce a phosphine radical. The latter is highly reactive and will abstract an H atom from DIPEA itself or one of its oxidized degradation products. The thereby generated secondary phosphine can react with the remaining chlorophosphine in the presence of DIPEA base in a child–parent reaction to form the observed diphosphine (Figure 4). In the case of more bulky substrates such as *t*Bu_2_PCl (**1d**), diphosphine formation is hampered and the secondary phosphine **3d** is obtained. When the reduction of **1d** is performed in CD_3_CN, no deuterium incorporation is observed in **3d**, which supports the notion that DIPEA is the major hydrogen atom donor. This assignment is consistent with recent deuterium isotope labeling experiments reported by Rothfelder et al.^11^ An alternative mechanism that involves halogen atom abstraction from the substrate by DIPEA^+^ or one of its decomposition products cannot be ruled out completely^28^ but would be at odds with the experimental finding that Ir^II^ reacts with R^Ar^_2_PX.

## Cyclophosphines from Dihalophosphines

The methodology developed above can be extended to dihalophosphines, the reduction of which can be used to synthesize cyclophosphines.^29^ For example, irradiation of MesPX_2_ for 7 h under the standard photoredox conditions in CH_3_CN leads to the formation of cyclotriphosphine (PMes)_3_ (**5j**) as the major product in a 26% yield (Table 3, entry 1). The ^31^P NMR spectrum of **5j** features a doublet and a triplet at δ = −109 and −142 ppm, respectively, with a coupling constant of ^1^*J*_P–P_ = 184 Hz, consistent with the literature data.^30^ Applying the knowledge gained above, a change of the solvent to a mixture of CH_3_CN and pentane (1:4) results in an increased yield of 57%, albeit after 2 days of irradiation (Table 3, entry 2). Faster reaction times and even higher yields (76%) can be obtained when exclusively **4j-Br** is used for the formation of compound **5j** (Table 3, entry 4). On a preparative scale, isolated yields drop to 35% due to the oxidative degradation of **5j** during purification (see Supporting Information for details). The even more bulky Mes* group (Mes* = 1,3,5-*t*BuC_6_H_2_) in Mes*PCl_2_ prevents the formation of cyclic products, and linear species can be identified (Table 3, entry 6). For all examples, it is important to control the reaction times as excessive illumination results in secondary chemistry and the decomposition of the cyclic phosphines. Again, this observation parallels the reactivity of the arylated diphosphines.

**Table 3:**
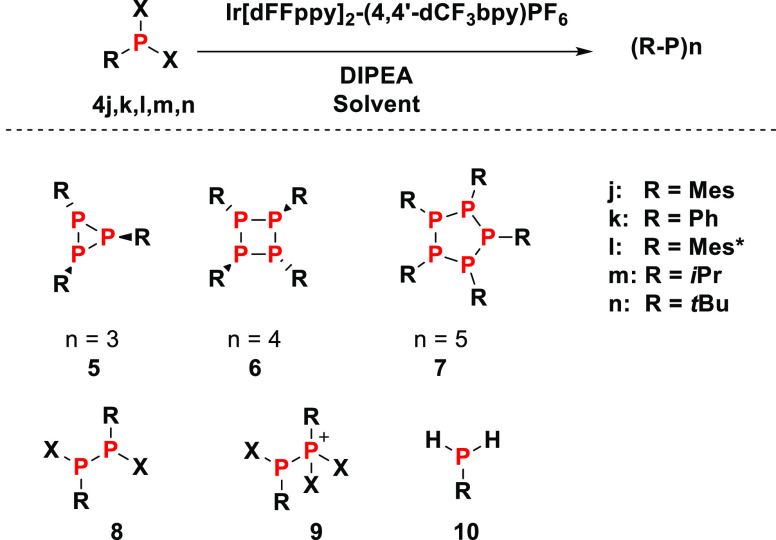
Reaction Scope and Conditions for Photochemical Reduction of Dihalophosphines **4j–n**

entry	substrate	solvent^a^	time	product	yield (%)
1	MesPX_2_	ACN	7 h	**5j**	26
2	MesPX_2_	pentane/ACN	2 days	**5j**	57
3	MesPBr_2_	ACN	7 h	**5j**	17
4	MesPBr_2_	pentane/ACN	7 h	**5j**	76
5	PhPBr_2_	pentane/ACN	17 h	**5k**	1
				**6k**	9
				**7k**	26
6	Mes*PCl_2_	ACN	24 h	**8l-H**	7
				**10L**	17
7	*i*PrPCl_2_	pentane/ACN	2 days	**6m**	20
8	*t*BuPCl_2_	ACN	4 days	**6n**	14
				**8n-Cl**	23
9	*t*BuPBr_2_	pentane/ACN	11 days	**6n**	49
				**8n-Br**	33
				**9n**	9

a Reactions were performed in Young-type NMR tubes in 0.5 mL of the solvent (ACN or pentane/ACN mixture 0.4/0.1 mL) with 150 μL of DIPEA and ca. 0.15 mol % of Ir[dFFppy]_2_-(4,4′-dCF_3_bpy)PF_6_. Reaction time corresponds to the irradiation time. Tris(4-fluorophenyl)phosphine was used as an internal standard to determine reaction yields.

Decreasing the size of the P-substituent gives access to larger cyclophosphines. For example, PhPBr_2_ results in the formation of cyclopentaphosphine **7k** in a 26% yield, accompanied by the P_4_-and P_3_-cycle **6k** and **5k** in 9 and 1% yields, respectively (Table 3, entry 5). The corresponding PhPCl_2_ results in very low yields of (PhP)_5_, together with trace amounts of multiple compounds including (PhP)_2_, (PhP)_3_, (PhP)_4_, (PhP)_6_, and PhPH_2_.

Dihalophosphines with aliphatic substituents as in **4m** (*i*Pr) and **4n** (*t*Bu) give cyclotetraphosphines **6m** and **6n** in 20 and 49% yields, respectively, as major products (Table 3 entries 7 and 9). These P_4_ cycles are characterized by one low-field resonance in their respective ^31^P NMR spectra at δ = −55.9 and −60.2 ppm for **6m** and **6n**, respectively^31,32^ The latter NMR spectrum also features signals that are assigned to a diastereomeric mixture of diphosphine **9n** [**9n-Br**: δ = 130 ppm (major), 149 ppm (minor)]. No signals of cyclotriphosphines **5m**, **n** were observed.

Therefore, dependent on the size of the substituents, three-, four-, or five-membered cyclophosphines can be predominantly formed under the applied conditions. Monosubstituted phosphorus dibromides give better results compared to the corresponding chlorides, which is consistent with the observations above. CH_3_CN as a solvent gives faster reaction times, however, at the expense of secondary photoredox processes.

## Conclusions

In summary, we have shown for the first time that halophosphines can be activated by an Ir^III^-based photocatalyst to form secondary phosphines. The reactions proceed under mild conditions and low catalyst loading (0.1 mol %) and are appealing alternatives to commonly used chemical pathways, using, for example, LiAlH_4_ or related highly reactive compounds as reductants. Under the experimental conditions, the secondary phosphines engage in base-promoted parent–child reactions to form diphosphines **2**, except in cases with excessive steric bulk. Diphosphines with aryl substituents can subsequently be reduced to secondary phosphine **3** in a second photocatalytic process. The overall yields for the formation of **3** from **1** can be as high as in the mid-80% range under optimized conditions, while the overall product distribution and kinetics of the reaction are sensitive to the nature of the P-substituent (aliphatic or aromatic), solvent, and nature of the halide.

Dihalophosphines can be reduced under identical conditions, giving rise to cyclic products, the size of which depends on the steric bulk of the P-substituents. It is worth mentioning that some of the obtained phosphorus cycles are challenging to obtain by other methods but become accessible through the photoredox catalysis route, presumably due to the low concentrations of reduced intermediates that are present at any given point in time.

The mechanism for the photoactivation of the halophosphines was investigated by transient absorption spectroscopy. As expected, DIPEA reductively quenches the excited state of the photocatalyst in a diffusion-controlled reaction to produce the Ir^II^ state, which, however, recombines with oxidized DIPEA^+^ faster than a productive electron transfer to the substrate. Together with the low catalyst loading that was used in the study, the charge recombination explains the experimentally observed reaction times. Irradiation on an hour time scale is, however, not at all uncommon in organic photoredox catalysis, raising the question of whether similar recombination phenomena are also a considerable factor in other systems. As DIPEA is not a perfectly reversible donor, some of the Ir^II^ state escapes recombination, and additional experiments on the seconds time scale show that it is this population that drives the reduction of the halophosphines.

We believe that the obtained results not only show a new pathway to secondary phosphines and intriguing OPCs but also highlight future directions in the field. Such projects could include the use of irreversible electron donors to accelerate reaction kinetics, donors that do not possess hydrogen atoms and that cannot interfere with the produced P-centered radical, and synthetic manipulations on the phosphorus precursors to avoid the parent–child reaction that is responsible for the formation of **2**. The realization of such protocols is the subject of ongoing work in our laboratory.

## Supporting Information Available

Details to the synthetic procedures and additional characterization material (PDF)
