# Supplementary material for: Mechanistic Insights and Synthetic Explorations of the Photoredox-Catalyzed Activation of Halophosphines
Source: Inorg Chem. 2023 Oct 19;62(45):18391–8. doi: 10.1021/acs.inorgchem.3c01946 (PMC10647117; doi:10.1021/acs.inorgchem.3c01946)
Supplement: Supplementary file 1 — ic3c01946_si_001.pdf [file ic3c01946_si_001.pdf]

Supporting information

Mechanistic Insights and Synthetic Explorations of the Photoredox-Catalyzed Activation of Halophosphines

*Anna I. Arkhypchuk, Thuan T. Tran, Rima Charaf, Leif Hammarström, Sascha Ott**

Department of Chemistry – Ångström Laboratory

Uppsala University

Box 523, 751 20 Uppsala

KEYWORDS: Photoredox chemistry, Diphosphine, main group chemistry, photoactivation, NMR spectroscopy.

Table of Contents

1. General experimental information ... 3

 1.1 Synthetic considerations ... 3

 1.2 Reaction yield determination based on ^{31}P NMR spectra ... 3

2. Preparation of the starting halophosphines ... 4

 2.1 Chloro/Bromo(mesityl)(phenyl)phosphine 1g ... 4

 2.2 Chloro/Bromo(naphthalen-1-yl)(phenyl)phosphine 1h ... 4

 2.3 Chloro/Bromodimesitylphosphine (1f) ... 5

 2.4 Chlorodiethylphosphine ... 5

 2.5 Dichloro(2,4,6-tri-tert-butylphenyl)phosphine ... 6

 2.6 Preparation of the bromophosphines from chlorophosphines: general protocol ... 6

  2.6.1 Bromodiphenylphosphine ... 6

  2.6.2 Bromodi-tert-butylphosphine ... 7

  2.6.3 Dibromo(phenyl)phosphine ... 7

  2.6.4 Dibromo(tert-butyl)phosphine ... 7

  2.6.5 Dibromo(mesityl)phosphine ... 7

3. Photochemical activation of phosphines ... 8

 3.1. Activation of monohalophosphines (R^{Alk}_2P-X and R^{Ar}_2P-X, X = Cl, Br) ... 8

 3.2. Activation of dihalophosphines ... 14

4. Cyclic voltammograms of representative halophosphines and diphosphines. ... 18

5. Emission quenching and UV-Vis spectra studies ... 19

6. NMR Spectra ... 24

7. References ... 50

1. General experimental information

1.1 Synthetic considerations

Unless specified otherwise, all manipulations were carried out using standard Schlenk line or glovebox techniques, in order to exclude air and moisture. Glassware was flame-dried, CH_3CN, toluene, THF and pentane were freshly taken from a solvent purification system under argon. DIPEA was freshly distilled from KOH and deoxygenated using standard freeze-pump-thaw technique (3 cycles). CD_3CN was dried by refluxing several hours under powdered CaH_2, distilled under argon and deoxygenated using standard freeze-pump-thaw technique (3 cycles). Commercially available mono and dichlorophosphines were purchased from Sigma Aldrich and distilled prior to use under argon. The syntheses of non-commercial halophosphines are described below. Iridium catalyst (CAS 2030437-92-2) was purchased from Sigma Aldrich and was used as received. NMR spectra were recorded on a JEOL (400YH magnet) Resonance 400 MHz spectrometer. Chemical shifts δ are reported in ppm and coupling constants *J* in Hz. ^{1}H NMR and ^{13}C NMR chemical shifts are referenced to the residual protic solvent signal and ^{31}P NMR spectra externally to 85% H_3PO_4(aq). High-resolution mass spectra (HR-MS) were recorded on a Thermo Scientific Orbitrap LTQ XL spectrometer.

1.2 Reaction yield determination based on ^{31}P NMR spectra

In order to find the best NMR parameters that would give a reliable integration of the signals in the ^{31}P NMR spectra, several experiments were performed. The parameters that were explored were the number of scans, the relaxation delay and the pulse angle. Yields obtained from ^{31}P NMR spectra were compared to those obtained from the integration of quantitative ^{1}H NMR spectral signals. The optimal parameters for accurate yield determination were found to be 128/256 scans with 20s relaxation delay with 45° pulse. These parameters were used throughout the paper.

Yields were calculated as following:

- ✓ Tris(4-fluorophenyl)phosphine was used as internal standard. Its ^{31}P NMR spectrum has one signal, the integration of which was always set to 1. Number of moles corresponding to this signal is calculated for each measurement (n(standard) mmol = m (added to NMR tube, mg)/M (316.26 g/mol)). The integral of the internal standard and product was determined using JEOL Delta 6.1.0 software.
- ✓ All products of the reaction are integrated and their concentrations are determined by the following formula: n (product) mmol = n (standard) mmol * integral value / number of contributing ^{31}P atoms.
- ✓ Yield of each product is calculated according to the formula:
  Yield % = (n (product) mmol * number of contributing ^{31}P atoms *100%) / n (starting Phosphine) mmol.

2. Preparation of the starting halophosphines

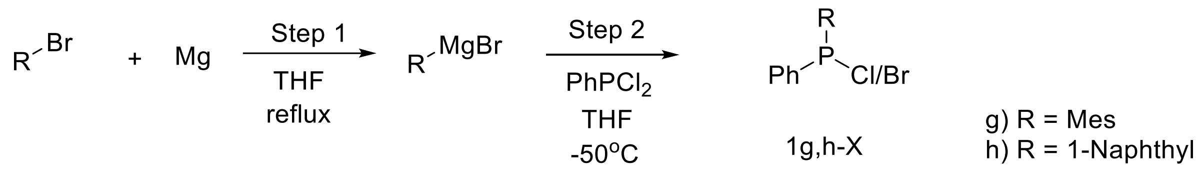

Scheme S1: Preparation of the monohalophosphines **1g, h** via Grignard reagent from $PhPCl_2$

2.1 Chloro/Bromo(mesityl)(phenyl)phosphine 1g

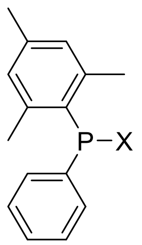

A flame-dried three neck flask was equipped with a reflux condenser and a dropping funnel, and flushed with argon. Magnesium turnings (0.87g, 0.036 mol, 1.1 eq.) were added together with a crystal of iodine, and the reactor was heated for several minutes with a heat gun under vigorous stirring. After the system had cooled down to r.t, 50 ml of dry THF was added to the magnesium. A dropping funnel was charged with a solution of mesityl bromide (6.5g, 0.033 mol, 1eq.) in 10 ml THF. The reaction mixture was brought to reflux and mesityl bromide was dropwise added to the magnesium under constant reflux. After completed addition (ca 25 min), the reaction mixture was allowed to reflux for 2 h. During this time, the colour of the solution changed to brown-grey under dissolution of the magnesium turnings. At this point, the reaction mixture was allowed to cool down to room temperature.

A second three neck flask was charged with 150 ml of THF and 6.44 g (0.036 mol, 1.1 eq.) of dichlorophenylphosphine. The solution was cooled down to -40°C and the freshly prepared solution of mesityl magnesium bromide (from above) was added dropwise over 20 min via canula (Note: it is very important to filter off remaining magnesium metal upon transfer of the Grignard reagent from the first flask). After completed addition, the reaction mixture was allowed to warm up to r.t. and stirred for additional 2 h. After this, the solvent was removed under vacuum, and the remaining solid extracted with dry pentane (2*150 ml). The pentane extracts were combined, pentane was distilled off, and the remaining oil purified by fractional distillation in vacuum to give the final product as pale-yellow oil (b.p. 140°C/ 0.01 mmHg.). Product was obtained as 1:1.22 mixture of Cl:Br and was used without further separation. Yield 9.2 g, 97%. ^{31}P NMR (C_6D_6): δ = 82.9 (MesPhPCl) and 72.3 (MesPhPBr) ppm. The ^{31}P NMR chemical shift matched the literature values. [1]

2.2 Chloro/Bromo(naphthalen-1-yl)(phenyl)phosphine 1h

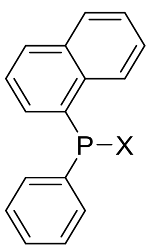

A flame dried three neck flask was equipped with a reflux condenser and a dropping funnel, andflushed with argon. Magnesium turnings (0.45g, 0.018 mol, 1.25 eq.) were added together with crystal of iodine, and reactor was heated for several minutes with a heat gun under vigorous stirring. After system had cooled down to r.t, 30 ml of dry THF was added to the magnesium. A dropping funnel was charged with a solution of 1-bromonaphthalene (3.2g, 0.016 mol, 1.05eq.) in 10 ml THF. Reaction mixture was brought to reflux and 1-bromonaphthalene was dropwise added to the magnesium under constant reflux. After completed addition (ca 15 min), the reaction mixture was allowed to reflux for 2 h. During this time, thecolour of the solution changed to brown-grey under dissolution of the magnesium turnings. At this point, the reaction mixture was allowed to cool down to room temperature.

A second three neck flask was charged with 150 ml of THF and 2.64 g (0.015 mol, 1 eq.) of dichlorophenylphosphine. The solution was cooled down to -40°C and freshly prepared solution of naphthalene magnesium bromide (from above) was added dropwise over 20 min via canula (Note: it is very important to filter off remaining magnesium metal upon transfer of the Grignard reagent from the first flask). After completed addition, the reaction mixture was allowed to warm up to r.t. and stirred for additional 2 h. After this, the solvent was removed under vacuum, and the remaining solid extracted with dry pentane (2*150 ml). The pentane extracts were combined, pentane was distilled off and the remaining oil purified by fractional distillation in vacuum to give the final product as yellow oil (b.p. 210°C/ 0.01 mmHg.). Product was obtained as 1:9 mixture of Cl: Br and was used without further separation. Yield 6g, 76%.^{31}P NMR (C_6D_6): δ = 80.5 (NaphtPhPCl) and 69.9 (NaphtPhPBr) ppm. The ^{31}P NMR chemical shift matched the literature values [2]

2.3 Chloro/Bromodimesitylphosphine (1f)

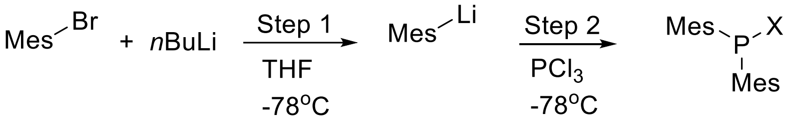

Scheme S2: Synthesis of halodimesitylphosphine

Mesityllithium was prepared by the dropwise addition of *n*BuLi (2.5 M in hexane, 11.4 mL, 0.028 mol, 2.1 eq) to a solution of Mes-Br (5.29g, 0.027 mol, 2 eq) in THF under argon at -78°C for 30 min. After addition, PCl_3 (1.82g, 0.013 mol, 1 eq) was added dropwise at -78°C. The mixture was allowed to warm to room temperature overnight to give an orange solution. The solvent was evaporated under vacuum and the remaining solid was extracted 3 times with pentane. Pentane was removed under vaccum, and the remaining oil distilled (b.p. 200°C/ 0.01 mm. Hg) to give 40% yield of Mes_2PX as a viscous pale-yellow oil that gradually solidified. ^{31}P NMR (C_6D_6): δ = 84.5 (Mes_2PCl) and 72.3 (Mes_2PBr) ppm. The ^{31}P NMR chemical shift matched the literature values.[3]

2.4 Chlorodiethylphosphine

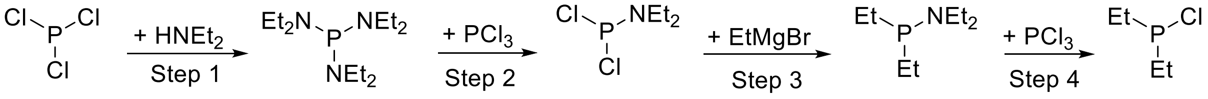

Scheme S3: Preparation of chlorodiethylphosphine

PCl_3 (3.29 mL, 0.04 mol, 1 eq) was added to a solution of 25 mL of $HNEt_2$ (0.242 mol, 6.05 eq) in 600 mL Et_2O at 0°C. The reaction mixture was stirred until complete conversion of the PCl_3 starting material (δ = 220 ppm) to product (δ = 120 ppm) had occurred, as monitored by ^{31}P NMR spectroscopy. This process took circa 1 hour. The obtained precipitate was filtered off and the Et_2O solvent was distilled off under Argon. The remaining oil was distilled under vacuum to yield the final product as a colorless oil (b.p 110-120°C/ 0.01 mmHg). Yield: 5.0 g, 51%. ^{31}P NMR (Et_2O): δ = 120 ppm. ^{31}P NMR chemical shift matches the literature values.[4]

The neat $P(NEt_2)_3$ obtained above (2.02g, 0.0082mol, 1eq) was then treated with PCl_3 (1.47 mL, 0.017 mol, 2.05 eq) at 0°C. The reaction mixture was stirred for 2 h and the product $PCl_2(NEt_2)$ was obtained as a colorless oil by distillation at around 30-40°C/ 0.01 mmHg. Yield: 3.04g, 71%. ^{31}P NMR (Et_2O): δ = 164 ppm. ^{31}P NMR chemical shift matches the literature values.[5]

$PCl_2(NEt_2)$ (2.85g, 0.0164 mol, 1eq) was then converted into $Et_2P(NEt_2)$ by the addition of EtMgCl (0.0336 mol, 2.05eq) in Et_2O at -40°C. The reaction mixture was stirred overnight. The obtained precipitate was filtered off and the solvent was removed. The desired product was obtained as colorless oil by distillation under vacuum at 40°C/ 0.01 mmHg. Yield: 1.708g, 65%. The compound $Et_2P(NEt_2)$ (1.09g, 0.0068 mol, 1eq) was directly treated with PCl_3 (0.59 mL, 0.0068 mol, 1eq) for 3 h to yield Et_2PCl. The product was isolated as colorless oil after distillation at 30°C/ 0.01 mmHg. Yield: 0.74g, 87%. ^{31}P NMR (C_6D_6): δ = 118.6 ppm. ^{31}P NMR chemical shift matches the literature values.[5]

2.5 Dichloro(2,4,6-tri-tert-butylphenyl)phosphine

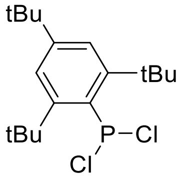

Dichloro(2,4,6-tri-tert-butylphenyl)phosphine was prepared according the literature procedure. ^{31}P NMR (ACN-D_3): δ = 153.9 ppm.[6]

2.6 Preparation of the bromophosphines from chlorophosphines: general protocol

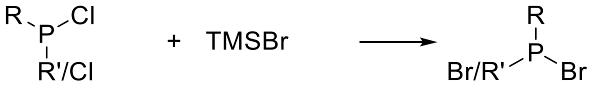

Scheme S4: Preparation of bromophosphines

Chlorophosphines were converted to the corresponding bromides according to **Scheme S4.** The neat chlorophosphine was treated with TMSBr (2 eq. per chloride to be exchanged) at room temperature if no additional information is stated. The reaction mixtures were stirred for 1h prior to distillation.

2.6.1 Bromodiphenylphosphine

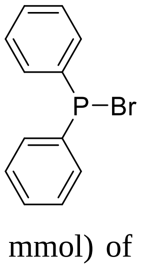

The reaction was performed on 1.8 g (8 mmol) of chlorodiphenylphosphine with 2.45g (16 mmol) of TMSBr at room temperature. The product was distilled at 100-105°C /0.01 mmHg,

and obtained as a pale-yellow oil. Yield: 1.92 g, 91%. ^{31}P NMR (C_6D_6): δ = 73.0 ppm. ^{31}P NMR chemical shift matches the literature value.[7]

2.6.2 Bromodi-tert-butylphosphine

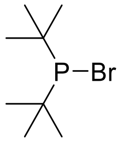

The reaction was performed on 0.95g (5.3 mmol) of chlorodi-tert-butyl phosphine with 1.6g (11 mmol) TMSBr at room temperature (30 min), followed by heating at 90°C for 4h. The product was distilled at 170°C /atm. Pressure, and obtained as a colorless oil. Yield: 0.92 g, 77%. ^{31}P NMR (ACN-D_3): δ = 151.7 ppm. ^{31}P NMR chemical shift matches the literature value.[8]

2.6.3 Dibromo(phenyl)phosphine

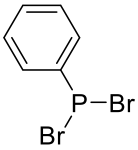

The reaction was performed on 1.3g (7.4 mmol) of dichlorophenyl phosphine with 4.5g (29.6 mmol) TMSBr at room temperature (1h), followed by heating at 60°C for 1h. The product was distilled at 85-90°C /0.01 mmHg, and obtained as a pale-yellow oil. Yield: 1.9 g, 96%. ^{31}P NMR (C_6D_6): δ = 151.1 ppm. ^{31}P NMR chemical shift matches the literature value.[9]

2.6.4 Dibromo(tert-butyl)phosphine

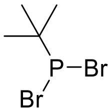

The reaction was performed on 0.67g (4.2 mmol) of dichlorotertbutyl phosphine with 2.6g (17 mmol) TMSBr at room temperature (30 min), followed by heating at 90°C for 3h. The product was distilled at 170°C /atm. Pressure, and obtained as a colorless oil. Yield: 0.62 g, 60%. ^{31}P NMR (ACN-D_3): δ = 204.8 ppm. ^{31}P NMR chemical shift matches the literature value.[10]

2.6.5 Dibromo(mesityl)phosphine

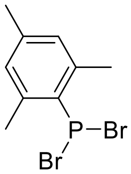

The reaction was performed on 1g (3.8 mmol) of dihalomesityl phosphine (mixture obtained from synthesis) with 1.7g (11.4 mmol) of TMSBr at room temperature (30 min), followed by heating at 90°C for 1h. The product was distilled at 120°C /0.01 mmHg, and obtained as a yellow oil. Yield: 1.1 g, 93%. ^{31}P NMR (Pentane): δ = 152.3 ppm. ^{31}P NMR chemical shift matches the literature value.[11]

3. Photochemical activation of phosphines

3.1. Activation of monohalophosphines ($R^{Alk}{}_2$P-X and $R^{Ar}{}_2$P-X, X = Cl, Br)

Typical protocol:

In a glove box, Young-type NMR tubes were charged with standard (tris(p-fluorophenyl)phosphine, ca 10 mg, exact amount stated in Table S1), substrate (ca 15-30 mg, exact amount used for each reaction stated in Table S1), solvent (0.4 ml plus 0.1 ml solution of the catalyst in the same solvent) and DIPEA (0.1 ml). The amount of catalyst was calculated to be ca 0.1 mol% per phosphorus atom and its solution was freshly prepared before each synthesis. After all components were added, the NMR tubes were closed and taken out of the glove box. A Kesill Tuna Blue lamp was used for irradiation (wavelength 460 nm, A160WE Tuna Blue light source, Power supply: 100-240 V AC (input), 19-24V DC (output), https://www.marine-aquatics.eu/en/kessil-a160we-tuna-blue-led-lighting-40w). The NMR tubes were placed on a laboratory shaker, directly exposed to the lamp (see the picture in Figure S1). NMR measurements were systematically performed to follow the reactions. The times stated in **Table S1** are irradiation times.

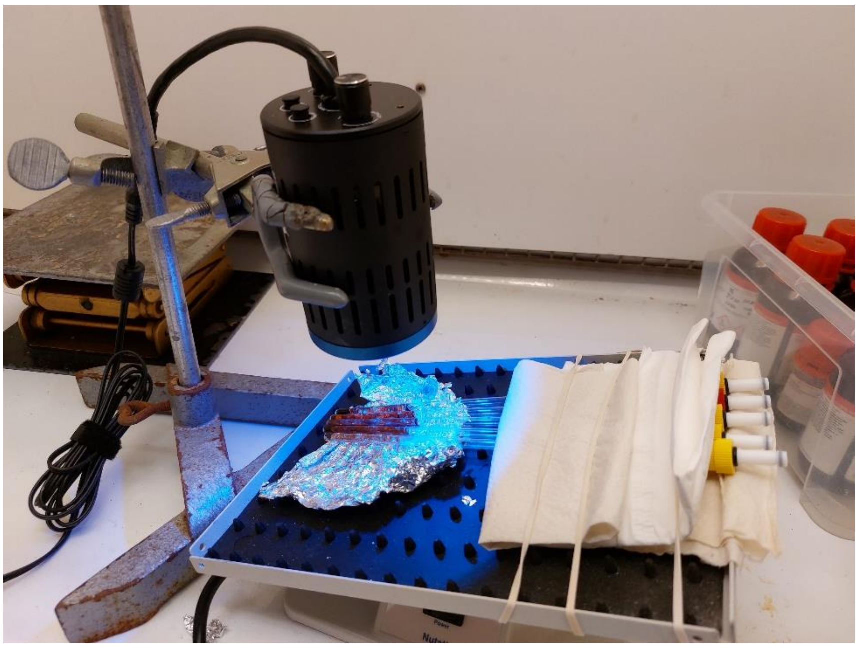

Figure S1: Irradiation set up with laboratory shaker and Kessil Tuna Blue lamp.

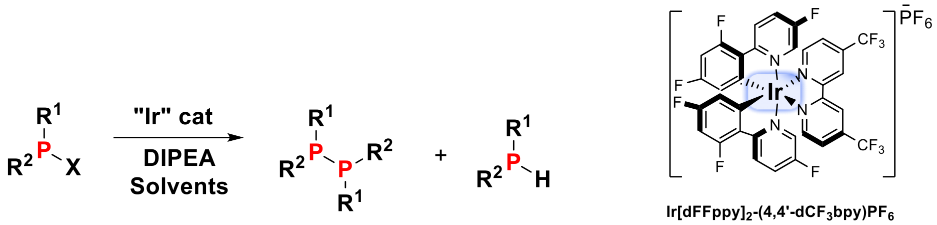

1a-h 2a-h 3a-h

Ir[dFFppy]$_2$-(4,4'-dCF$_3$bpy)PF$_6$

1,2,3a $R_1 = R_2 = i$Pr
1,2,3b $R_1 = R_2$ = Et
1,2,3c $R_1 = R_2$ = Cy
1,2,3d $R_1 = R_2 = t$Bu

1,2,3e $R_1 = R_2$ = Ph
1,2,3f $R_1 = R_2$ = Mes
1,2,3g R_1 = Ph, R_2 = Mes
1,2,3h R_1 = Ph, R_2 = Napht
1,2,3i R_1 = Ph, $R_2 = t$Bu

X = Cl/Br

Scheme S5: Photochemical activation of monohalophosphines **1a-1i**

Table S1. Reaction details on activation of monohalophosphines.

Entry	Substrate		m, mg (n mmol) of starting phosphine	m, mg (n, mmol) of standard	Solvent[a]	Time	Product	Integral	Yield	δ ^{31}P ppm (J_{PH} or J_{PP})	Ref.
1	iPr_2PCl	**1a**	19.2 mg (0.13)	10.4 mg (0.03)	ACN	36h	**2a**	2.68	70%	-10.0 (s)	[12]
2	Et_2PCl	**1b**	21.2 mg (0.17)	8.5 mg (0.03)	ACN	18h	**2b**	3.39	54%	-30.8 (s)	[12]
3	Cy_2PCl	**1c**	15.8 mg (0.07)	-	ACN-D3	12h	**2c**	-	100%	-20.8 (s, CCl_2D_2/C_6D_6)	[13]
4	*t*Bu_2PCl	**1d-Cl**	19.0 mg (0.11)	9.5 mg (0.03)	THF	104 d	**3d**	2.18	65%	+21.8 (d, J_{PH} = 198 Hz)	[14]
5	*t*Bu_2PBr	**1d-Br**	26.0 mg (0.12)	9.3 mg (0.03)	ACN	19 d	**3d**	3.43	89%	+19.9 (d, J_{PH} = 211 Hz)	[14]
6	Ph_2PCl	**1e-Cl**	22.6 mg (0.10)	9.9 mg (0.03)	ACN	15 min	**2e**	1.57	48%	-15.1 (s)	[12]
						45 min	**2e**	2.12	65%	-15.1 (s)	
						12h	**2e**	1.17	36%	-15.1 (s)	
							**3e**	086	26%	-37.8 (d, J_{PH} = 218 Hz)	[15]
						24h	**3e**	2.50	74%	-37.8 (d, J_{PH} = 218 Hz)	[15]
7	Ph_2PCl	**1e-Cl**	29.4 mg (0.13)	6.6 mg (0.02)	THF	30h	**2e**	4.33	68%	-13.5 (s)	[12]
8	Ph_2PCl	**1e-Cl**	30.0 mg (0.14)	11.0 mg (0.03)	Toluene	30 h	**2e**	2.06	53%	-13.0 (s)	[12]
9	Ph_2PCl	**1e-Cl**	29.4 mg (0.13)	9.3 mg (0.03)	Pentane	28d	**2e**	2.06	45%	-13.1 (s)	[12]
							**1e-Cl**	1.13	25%	+82.1 (s)	
10	Ph_2PBr	**1e-Br**	27.9 mg (0.11)	9.9 mg (0.03)	ACN	15 min	**2e**	2.19	65%	-15.1 (s)	[12]
						45 min	**2e**	2.29	68%	-15.1 (s)	[12]
						12h	**3e**	2.51	75%	-37.8 (d, J_{PH} = 218 Hz)	[15]
						24h	**3e**	2.77	82%	-37.8 (d, J_{PH} = 218 Hz)	[15]
11	Ph_2PBr	**1e-Br**	29.0 mg (0.11)	10.1mg (0.03)	THF	5 days	**2e**	2.05	59%	-14.2 (s)	[12]
12	Ph_2PBr	**1e-Br**	28.6 mg (0.11)	11.6mg (0.04)	Toluene	5 days	**2e**	1.61	55%	-14.1 (s)	[12]
13	Mes_2PX	**1f-X**	21.1 mg (0.06)	9.5 mg (0.03)	THF	7days	**2f**	1.24	59%	-28.6 (s)	[16]
							**3f**	0.04	2%	-92.3 (d, J_{PH} = 228 Hz)	
							**1f-Cl**	0.11	5%	87.7 (s)	
14	Mes_2PX	**1f-X**	20.6 mg(0.06)	10.6 mg (0.03)	Toluene	9 days	**2f**	0.93	51%	-28.5 (s)	[16]
							**1f-Cl**	0.29	16%	87.4 (s)	
15	Mes_2PX	**1f-X**	19.2 mg (0.06)	11.9 mg (0.04)	ACN	17h	**2f**	0.85	56%	-28.6 (s)	[16]

16	Mes_2PBr	**1f-Br**	21.9 mg (0.06)	9.8 mg (0.03)	ACN	3h	**2f**	1.34	66%	-28.7 (s)	[16]
17	Mes_2PBr	**1f-Br**	22.6 mg (0.06)	12.0 mg (0.04)	THF	2 day	**2f**	1.21	71%	-28 5 (s)	[16]
18	Mes_2PBr	**1f-Br**	23.9 mg (0.06)	9.1 mg (0.03)	Toluene	4 days	**2f**	1.59	67%	-28.4 (s)	[16]
19	MesPhPX	**1g-X**	24.6 mg (0.09)	11.0 mg (0.03)	ACN	4h	**2g**	0.16	7%	-29.5 (s)	[17]
								1.18	48%	-33.2 (s)	
						7 days	**3g**	1.58	63%	-74.3 (d, J_{PH} = 224 Hz)	
20	MesPhPBr	**1g-Br**	30.2 mg (0.10)	9.3 mg (0.03)	ACN	4h	**2g**	0.26	8%	-29.2 (s)	[17]
								2.09	63%	-32.8 (s)	
						7 days	**3g**	2.55	76%	-74.6 (d, J_{PH} = 222 Hz)	[18]
21			27.1 mg (0.09)	9.9 mg (0.03)	THF	6 days	**2g**	0.25	9%	-29.5 (s)	[17]
								1.52	54%	-33.2 (s)	
22			24. 6 mg (0.08)	10.8 mg (0.03)	Toluene	6 days	**2g**	0.20	9%	-29.2 (s)	[17]
								1.42	61%	-33.0 (s)	
23	Naphth-PhX	**1h-X**	27.9 mg (0.09)	11.1 mg (0.04)	ACN	45 min	**2h**	0.72	28%	-19.0 (s)	
								1.64	63%	-26.7 (s)	
						6 days	**2h**	0.51	19%	-19.0 (s)	
							**3h**	1.98	76%	-48.3 (d, J_{PH} = 223 Hz)	
24			19.9 mg (0.07)	6.6 mg (0.02)	THF	2 days	**2h**	0.8	24%	-19.0 (s)	
								2.01	60%	-25.6 (m)	
25			25.0 mg (0.08)	10.1mg (0.03)	Toluene	4d	**2h**	0.19	6%	-18.9 (s)	
								1.11	44%	-25.8 (s)	
							**1h-Cl**	0.51	20%	82.0 (s)	
26	Naphth-PhPBr	**1h-Br**	23.9 mg (0.08)	11.4 mg (0.04)	ACN	45 min	**2h**	0.59	28%	-18.9 (s)	
								1.35	64%	-26.6 (s)	
						6 day	**2h**	0.30	14%	-18.9 (s)	
							**3h**	1.73	83%	-48. 4 (d, J_{PH} = 223 Hz)	
27			22.5 mg (0.07)	10.4 mg (0.03)	THF	2 days	**2h**	0.52	24%	-19.0 (s)	
								1.32	61%	-25.7 (s)	
28			26.4 mg (0.08)	10.4 mg (0.03)	Toluene	6 days	**2h**	0.16	6%	-19.8 (s)	
								1.38	54%	-25.9 (s)	
29	PhtBuPCl	**1i**	21.3 mg (0.11)	9.48 mg (0.03)	ACN	2 days	**2i**	2.22	63%	-2.2 (s)	[19], [20]
								0.39	11%	4.6 (s)	
30		**1i**	19.1 mg (0.10)	12.2mg (0.049	THF	11days	**2i**	1.10	46%	-2.6 (s)	
								0.16	7%	3.8 (s)	
							**1i**	0.12	5%	110.0 (s)	

1,1,2,2-tetra-iso-propyldiphosphine (2a)

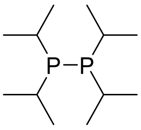

Compound was not isolated. ^{31}P NMR (ACN): δ = -10.0 ppm. Analytical data is in accordance with the literature.[12]

1,1,2,2-tetraethyldiphosphine (2b)

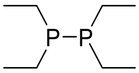

Compound was not isolated. ^{31}P NMR (ACN): δ = -30.8 ppm. Analytical data is in accordance with the literature.[12]

1,1,2,2-tetracyclohexyldiphosphine (2c)

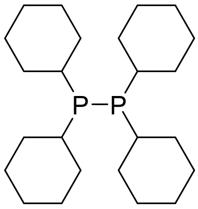

Compound crystalized out of ACN during the reaction and was isolated. Colorless crystals, 13.6 mg (0.034 mmol), 99%. ^{31}P NMR (CD_2Cl_2/C_6D_6): δ = -20.8 ppm; ^{1}H (CD_2Cl_2/C_6D_6) δ 1.86-1.26 ppm (44H, m, CH /CH_2); APT ^{13}C NMR (CD_2Cl_2/C_6D_6) δ 32.9, 31.4, 31.0, 27.9, 27.6, 26.8 ppm. Analytical data is in accordance with the literature.[13]

1,1,2,2-tetraphenyldiphosphine (2e)

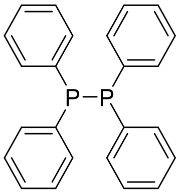

Compound was not isolated. ^{31}P NMR (ACN): δ = -15.1 ppm. Analytical data is in accordance with the literature.[12]

1,1,2,2-tetramesityldiphosphine (2f)

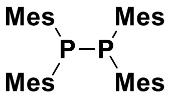

Compound was not isolated. ^{31}P NMR (THF): δ = -28.7 ppm. Analytical data is in accordance with the literature.[16]

1,2-dimesityl-1,2-diphenyldiphosphine (2g)

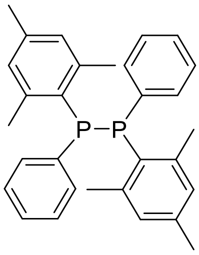

In a microwave vial equipped with a stirring bar, MesPhPBr (0.36 g, 1.2 mmol, 1eq), DIPEA (1.03 mL, 6 mmol, 5 eq), ''Ir'' catalyst (0.1% mol) were added to 5 mL acetonitrile in an argon filled glove box. The vial was sealed and irradiated with blue light. After 1 day, the part dissolved in ACN was removed to give white solid, that was washed 3 times with ACN (3mL) to remove all the DIPEA derivative. The addition of absolute ethanol caused the precipitation of **2g** as a white powder (0.14 g, 48%).

Compound was isolated. ^{31}P NMR (Benzene-D_6): δ = -30.1 and δ = -34.5 ppm. ^{1}H NMR (400 MHz, Benzene-D_6, major): δ = 7.58-7.56 (m, 4H), 7.00 – 6.91 (m, 6H), 6.49 (s, 4H), 2.34 (bs, 12H), 1.82 (bs, 6H) ppm. ^{1}H NMR (400 MHz, Benzene-D_6, minor): δ = 7.13-7.10 (m, 4H), 6.85 – 6.79 (m, 10H), 2.64 (bs, 12H), 2.06 (bs, 6H) ppm. APT ^{13}C NMR (101 MHz, Benzene-D_6, major): δ = 146.0 (t, *J* = 8 Hz), 141.3, 139.6, 131.6 (t, *J* = 13 Hz), 128.7, 126.9, 24.5 (t, *J* = 11 Hz), 20.9 ppm. Analytical data is in accordance with the literature.[17]

1,2-di(naphthalen-2-yl)-1,2-diphenyldiphosphine (2h)

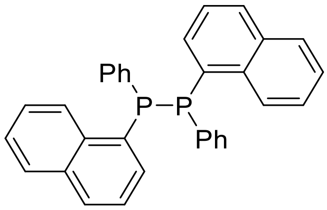

Compound was not isolated. ^{31}P NMR (ACN): δ = -19.1 and δ = -26.7 ppm indicating the simultaneous formation of *rac* and *meso* PhNaphtP-PPhNapht.

1,2-di-tert-butyl-1,2-diphenyldiphosphine (2i)

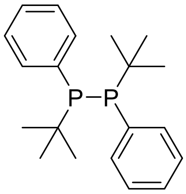

Compound was not isolated. ^{31}P NMR (ACN): δ = 4.6 and δ = -2.2 ppm indicating the simultaneous formation of *rac* and *meso* Ph(*t*Bu)P-P(*t*Bu)Ph. Analytical data is in accordance with the literature.[19-20]

3.2. Activation of dihalophosphines

Typical protocol:

In a glove box, Young type NMR tubes are charged with standard (tris(p-fluorophenyl)phosphine, ca 10 mg, exact amount stated in Table S2), substrate (ca 15-30 mg, exact amount used for each reaction stated in the Table S2), solvent (for reactions in ACN: 0.4 ml ACN plus 0.1 ml solution of the catalyst in ACN; for reactions in pentane/ACN: 0.4 ml of pentane plus 0.1 ml solution of the catalyst in ACN) and DIPEA (0.15 ml). The amount of catalyst was calculated to be ca 0.15 mol% per phosphorus atom and its solution was freshly prepared before each synthesis. After all components were added, the NMR tubes were closed and taken out of the glove box. Kesill Tuna Blue lamp was used for irradiation with full power settings. The NMR tubes were placed on a laboratory shaker, directly exposed to the lamp (see Figure S1). NMR measurements were systematically performed to follow the reactions. The times stated in **Table S2** are irradiation times.

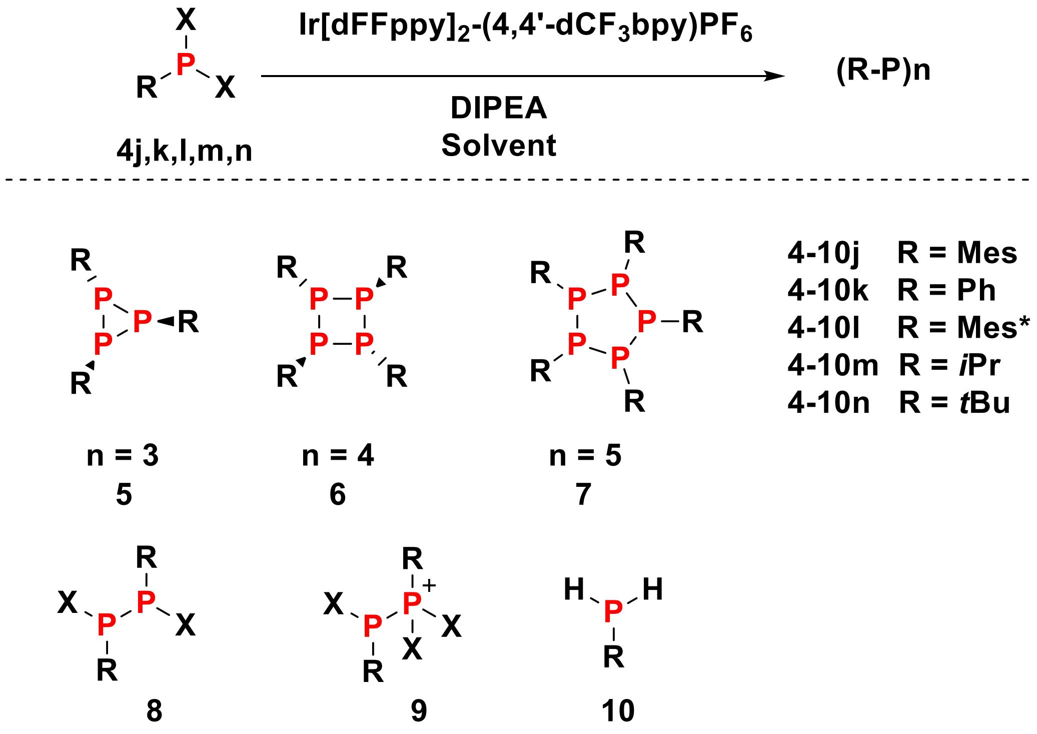

Scheme S6: Photochemical activation of dihalophosphines and obtained reaction products

Table S2. Reaction details on activation of dihalophosphines.

Entry	Substrate	R	m, mg (n, mmol) of starting phosphine	m, mg (n, mmol) of standard	Solvent[a]	Time	Product	Integral	Yield	$\delta^{31}P$ ppm (J_{PH} or J_{PP}, Hz)	Ref.
1	$MesPX_2$	Mes	27.2 mg (0.11)	9.05 mg(0.03)	ACN	7h	**5j**	0.67	26%	-108 (d, J_{PP} = 183 Hz)	[21]
								0.33		-146 (t, J_{PP} = 183 Hz)	
2	$MesPX_2$	Mes	27.2 mg (0.11)	13.6mg(0.04)	Pentane/ACN	34h	**5j**	0.98	57%	-108 (d, J_{PP} = 184 Hz)	[21]
								0.48		-142 (t, J_{PP} = 184 Hz)	
							**6d**	0.12		-42.0 (s)	
3	$MesPBr_2$	Mes	30.6mg (0.10)	9.05mg (0.03)	ACN	7 h	**5j**	0.40	17%	-108 (d, J_{PP} = 184 Hz)	[21]
								0.20		-142 (t, J_{PP} = 184 Hz)	
4	$MesPBr_2$	Mes	30.0 mg (0.10)	12.1 mg (0.04)	Pentane/ACN	7 h	**5j**	1.60	76%	-108 (d, J_{PP} = 184 Hz)	[21]
								0.81		-142 (t, J_{PP} = 184 Hz)	
5	Mes^*PCl_2	Mes*	33 mg (0.095)	12.6 mg (0.04)	ACN	24 h	**10l**	0.41	17%	-131.1 (t, J_{PH} = 209 Hz)	[22]
							**8l-H**	0.16	7%	-79.5 (dd, J_{PH} = 184, 17 Hz)	[23]
6	$PhPBr_2$	Ph	40.2 mg (0.15)	15.2 mg (0.05)	Pentane/ACN	17h	**5k**	0.02	≤1%		[24]
							**6k**	0.28	9%	-46.3 (s)	[25]
							**7k**	0.82	26%	-0.5 to +2 (m)	[26]
7	*i*$PrPCl_2$	*i*Pr	25.6 mg (0.18)	11.2 mg (0.04)	Pentane/ACN	2 days	**6m**	0.99	20%[1)]	-60 (s)	[27]
8	*t*$BuPCl_2$	*t*Bu	19.0 mg (0.12)	8.8 mg (0.03)	ACN	4 days	**6n**	0.61	14%	-57 (s)	[28]
							**8n-Cl**	0.97	23%	136	

9	*t*BuPBr$_2$	*t*Bu	36.7 mg (0.15)	10 mg (0.03)	Pentane/ACN	11 days	**6n**<br>**8n-Br**<br>**9n**	2.28<br>1.56<br>0.43	49%<br>33%<br>9%	-56 (s)<br>129.0<br>149<br>132<br>-1.2	[28]
10	*t*BuPBr$_2$	*t*Bu	19.6 mg (0.08)	10.5 mg (0.03)	ACN	6h	**6n**<br>decomposed	0.01	4%	-56 (s)	

1)The ^{31}P NMR spectrum shows four additional signals that are tentatively assigned to a linear version of a P_4 species. This compound is the major product, obtained in ca. 70 % crude yield.

(R)-1,2,3-trimesityltriphosphirane

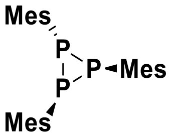

In a microwave vial equipped with a stirring bar, $MesPBr_2$ (0.33 g, 1.03 mmol, 1eq), DIPEA (1.03 mL, 7.9 mmol, 7.5 eq), ‘‘Ir’’ catalyst (0.15% mol) were added to 5 mL mixture solvent of pentane : acetonitrile (4:1) in an argon filled glove box. The vial was sealed and irradiated with blue light. After 1 day, the part dissolved in pentane (product) was extracted. The pentane solvent was evaporated under reduced pressure and the obtained solid residue was washed twice with ACN (3mL) to give final product **5j** as white powder (0.055 g, 35%).

Compound was isolated. ^{31}P NMR (THF-D_8): δ = -109.7 (d, $^1J_{P\text{-}P}$ = 184.1 Hz) and -144.6 (t, $^1J_{P\text{-}P}$ = 183.4 Hz) ppm. ^{1}H NMR (THF-D_8): δ = 6.76 (d, *J* = 3 Hz, 2H), 6.58 (s, 4H), 2.66 (s, 6H), 2.32 (s, 12H), 2.17 (s, 3H), 2.10 (s, 6H) ppm. APT ^{13}C NMR (101 MHz, THF-D_8): δ = 143.9 (t, *J* = 7.0 Hz), 142.2 (d, *J* = 9.6 Hz), 137.9, 137.2, 128.0, 127.7, 23.7 (dt, *J* = 11.8, 6.1 Hz), 23.4 (dd, *J* = 7.2 Hz), 20.0, 19.9 ppm. Analytical data is in accordance with the literature.[21]

(2,4,6-tri-*tert*-butylphenyl)phosphane

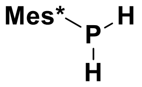

Compound was not isolated. ^{31}P NMR (ACN): δ = -131 ppm (dd, $^1J_{P\text{-}H}$ = 209 Hz). Analytical data is in accordance with the literature.[22]

1,2-bis(2,4,6-tri-*tert*-butylphenyl)diphosphane

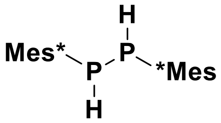

Compound was not isolated. ^{31}P NMR (ACN): δ = -79.5 (dd, $^1J_{P\text{-}H}$ = 184 Hz). Analytical data is in accordance with the literature.[23]

(1r,2r,3r,4r)-1,2,3,4-tetraisopropyltetraphosphetane

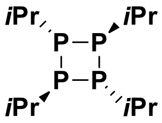

Compound was not isolated. ^{31}P NMR (Pentane:ACN = 0.5:0.1): δ = -60 ppm. Analytical data is in accordance with the literature.[27]

(1r,2r,3r,4r)-1,2,3,4-tetra-tert-butyltetraphosphetane

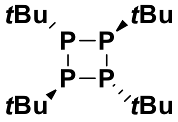

Compound was not isolated. ^{31}P NMR (Pentane:ACN = 0.5:0.1): δ = -55.9 ppm. Analytical data is in accordance with the literature.[28]

4. Cyclic voltammograms of representative halophosphines and diphosphines.

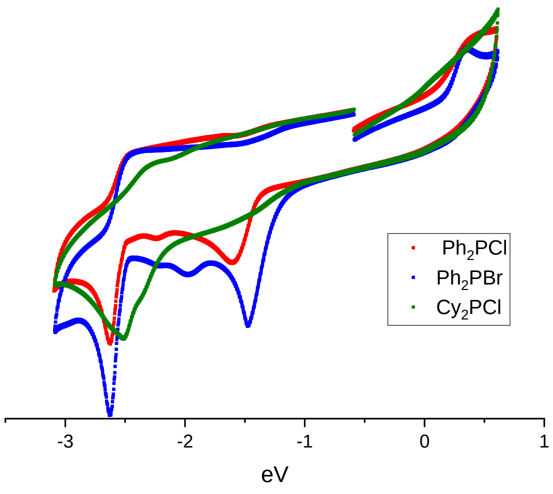

Figure S2: Cyclic voltammograms of Ph_2PCl (red), Ph_2PBr (blue), and Cy_2PCl (green). Voltammograms recorded in 0.1 M $[NBu_4][PF_6]$ CH_3CN solution at scan rate of 100 mV/s; with carbon working electrode, platinum wire counter electrode, and a silver wire reference electrode.

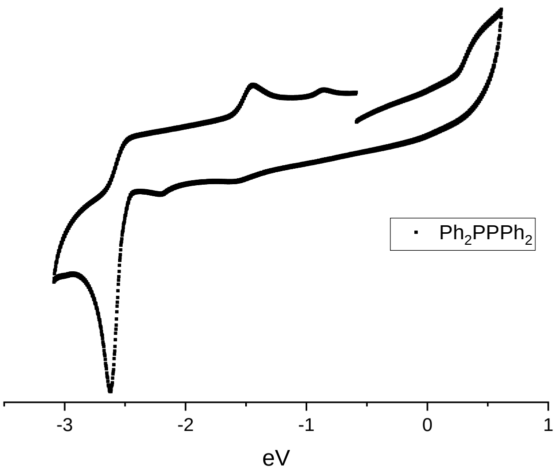

Figure S3: Cyclic voltammogram of Ph_4P_2 (black), recorded in 0.1 M $[NBu_4][PF_6]$ CH_3CN solution at scan rate 100 mv/s, with carbon working electrode, platinum wire counter electrode, and a silver wire reference electrode. The corresponding CV of the tetra-cyclohexyl analogue Cy_4P_2 shows no feature within the solvent window.

5. Emission quenching and UV-Vis spectra studies

Emission spectra were recorded on a Horiba Jobin Yvon Fluorolog, with the excitation wavelength set to 380 nm, slits at 1 nm and integration time of 1 s.

For the transient absorption measurements, the samples were excited using a Nd:YAG laser (Ekspla, NT342B laser) with an OPO set at 410 nm and energies of 7-9 mJ/pulse. The spectrometer (LP920, Edinburgh Instruments) consisted of a pulsed 450 W ozone-free Xe arc lamp, a symmetrical Czerny-Turner monochromator (TMS300) with 5 nm bandwidth and detectors for either single kinetic traces (LP900 photomultiplier, with Tektronix TDS3012C oscilloscope) or entire spectra (Andor SH720 ICCD camera).

The quenchers were added to a 2 mL solution of Ir[dFFppy]2-(4,4'-dCF3bpy)PF_6 50 µM in ACN. The excitation wavelength was set to 380 nm. The fluorescence was measured and the plots were constructed according to the Stern-Volmer equation:

$\frac{I_0}{I} = 1 + k_Q \tau_0 [Q]$, where I_0 and I are the emission intensities at the emission maximum (620 nm) without and with quencher, respectively, k_Q is the kinetic constant of quenching, τ_0 is the measured lifetime in absence of quencher (293 ns for Ir[dFFppy]$_2$-(4,4'-dCF_3bpy)PF_6 in ACN) and [Q] is the concentration of quencher. The quenching constants for DIPEA, Cy_2PCl, Ph_2PCl and Ph_2PBr were $k_{Q\,(DIPEA)} = 1.1 \times 10^{10} M^{-1}s^{-1}$, $k_{Q\,(Cy2PCl)} = 1.2 \times 10^{8} M^{-1}s^{-1}$, $k_{Q\,(Ph2PCl)} = 1.5 \times 10^{8} M^{-1}s^{-1}$, and $k_{Q\,(Ph2PBr)} = 1.7 \times 10^{8} M^{-1}s^{-1}$ respectively.

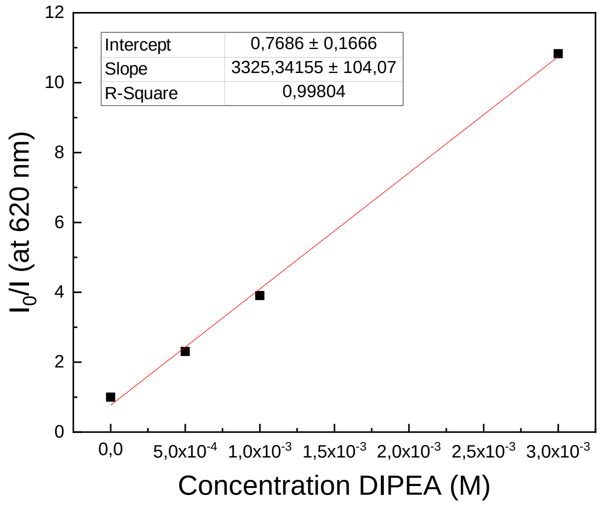

Figure S4: Stern-Volmer plot for the emission quenching of Ir[dFFppy]$_2$-(4,4'-dCF_3bpy)PF_6 with DIPEA in ACN

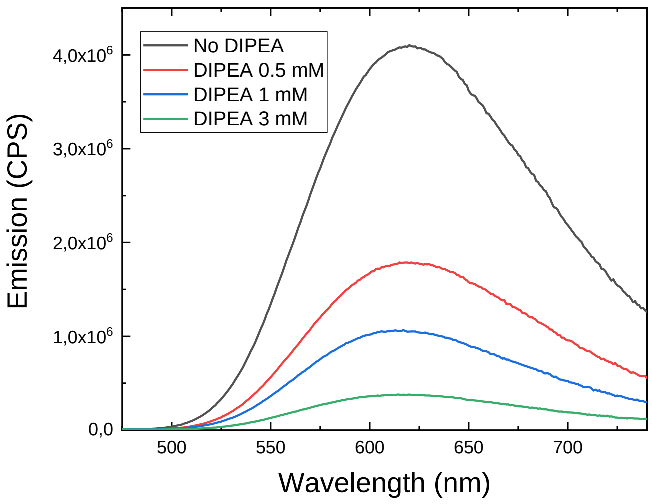

Figure S5: Emission spectra of Ir[dFFppy]$_2$-(4,4'-dCF$_3$bpy)PF$_6$ at different concentration of DIPEA in ACN.

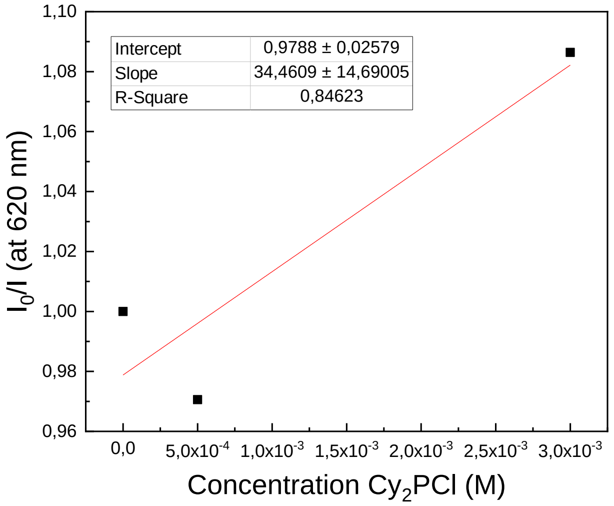

Figure S6: Stern-Volmer plot for the emission quenching of Ir[dFFppy]$_2$-(4,4'-dCF$_3$bpy)PF$_6$ with Cy_2PCl in ACN.

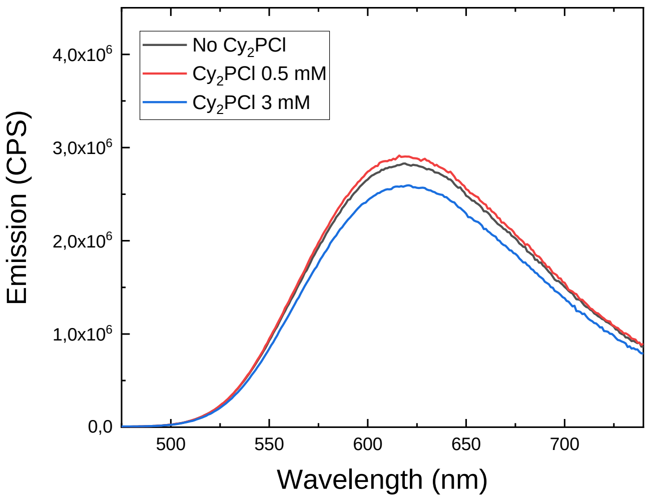

Figure S7: Emission spectra of Ir[dFFppy]$_{2}$-(4,4’-dCF$_{3}$bpy)PF$_{6}$ at different concentration of Cy2PCl in ACN.

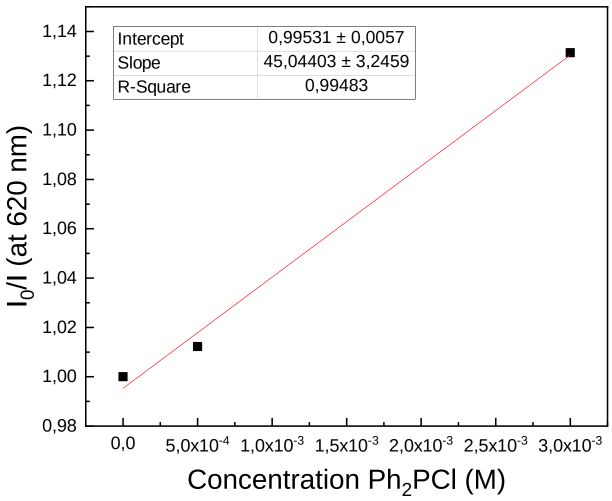

Figure S8: Stern-Volmer plot for the emission quenching of Ir[dFFppy]$_{2}$-(4,4’-dCF$_{3}$bpy)PF$_{6}$ with Ph_2PCl in ACN

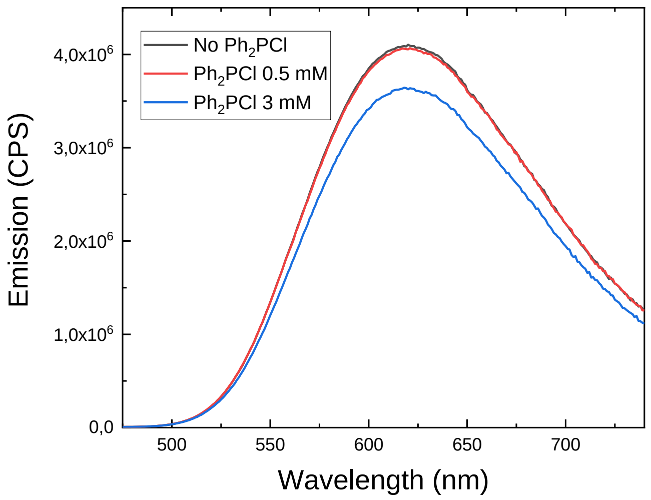

Figure S9: Emission spectra of Ir[dFFppy]$_2$-(4,4'-dCF$_3$bpy)PF$_6$ at different concentration of Ph_2PCl in ACN.

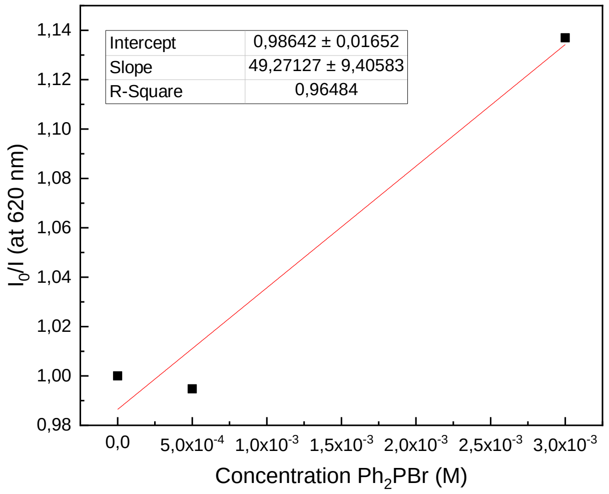

Figure S10: Stern-Volmer plot for the emission quenching of Ir[dFFppy]$_2$-(4,4'-dCF$_3$bpy)PF$_6$ with Ph_2PBr in ACN.

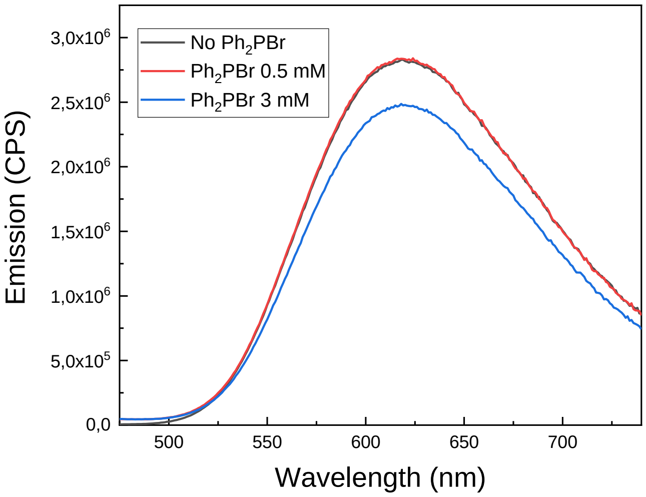

Figure S11: Emission spectra of Ir[dFFppy]$_{2}$-(4,4’-dCF$_{3}$bpy)PF$_{6}$ at different concentration of Ph_2PBr in ACN.

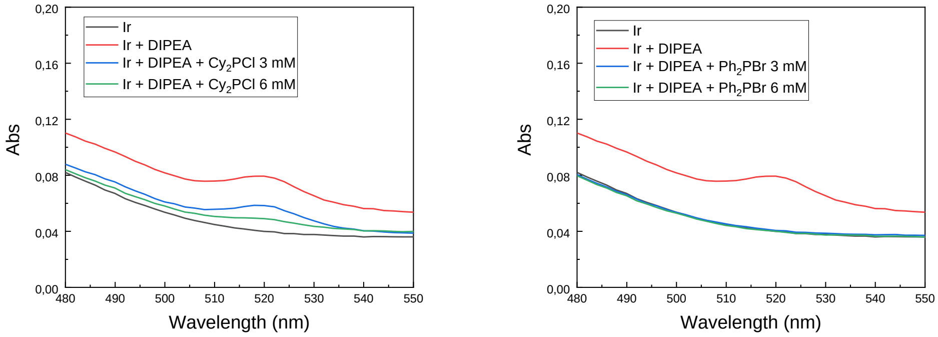

Figure S12: Steady state UV-Vis spectra of Ir[dFFppy]$_{2}$-(4,4’-dCF$_{3}$bpy)PF$_{6}$ 100 µM in the presence of DIPEA 3 mM and two different concentrations of substrates (Cy_2PCl and Ph_2PBr).

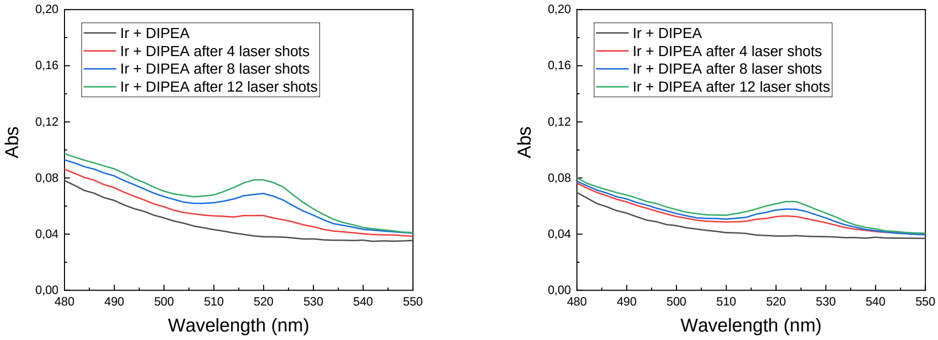

Figure S13: Steady state UV-Vis spectra of Ir[dFFppy]$_{2}$-(4,4’-dCF$_{3}$bpy)PF$_{6}$ 100 µM and DIPEA 3 mM in ACN (left) and Toluene (right) after different amounts of laser shots at 410 nm and 7 mJ/pulse.

6. NMR Spectra

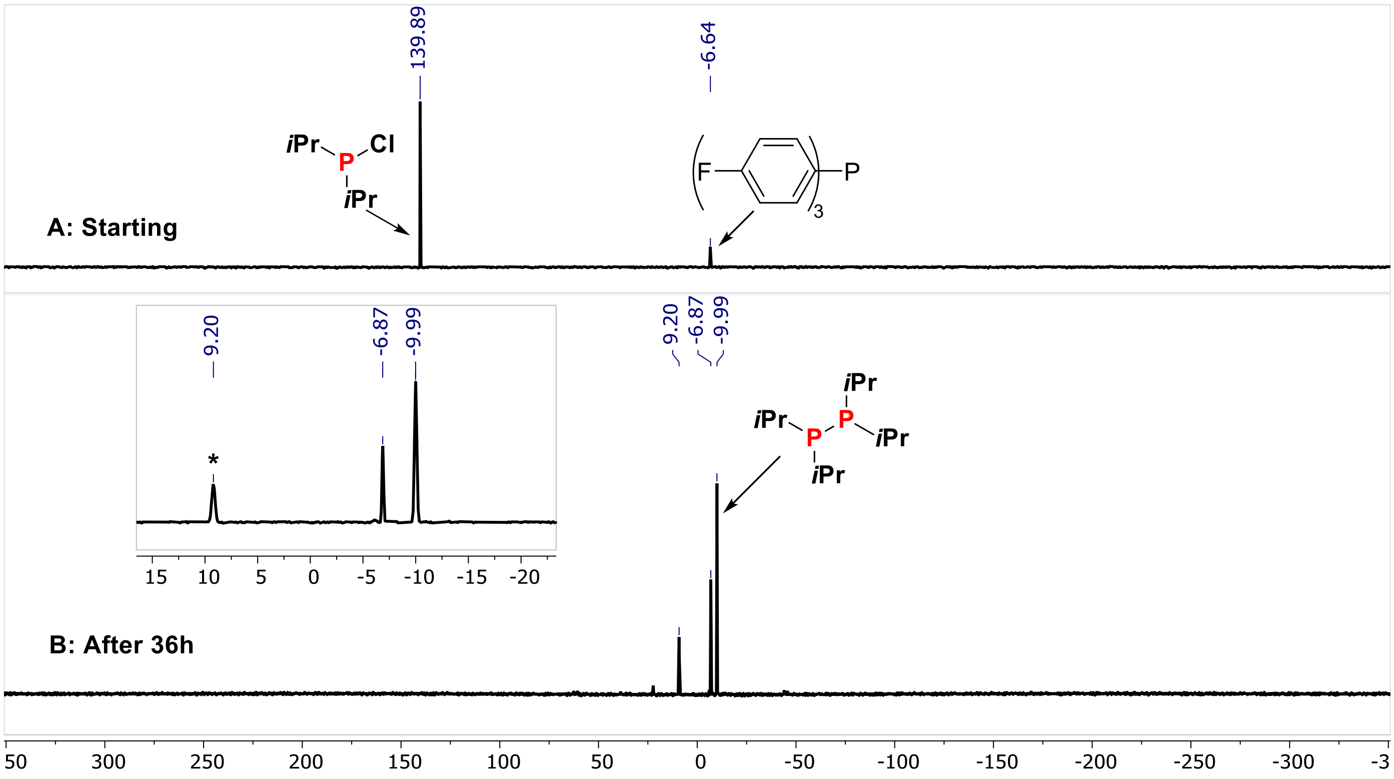

Figure S14: Crude ^{31}P NMR spectrum for **2a** using tris(4-fluorophenyl)phosphine as internal standard (Entry 1, Table S1). * marks the signal of an unknown by-product.

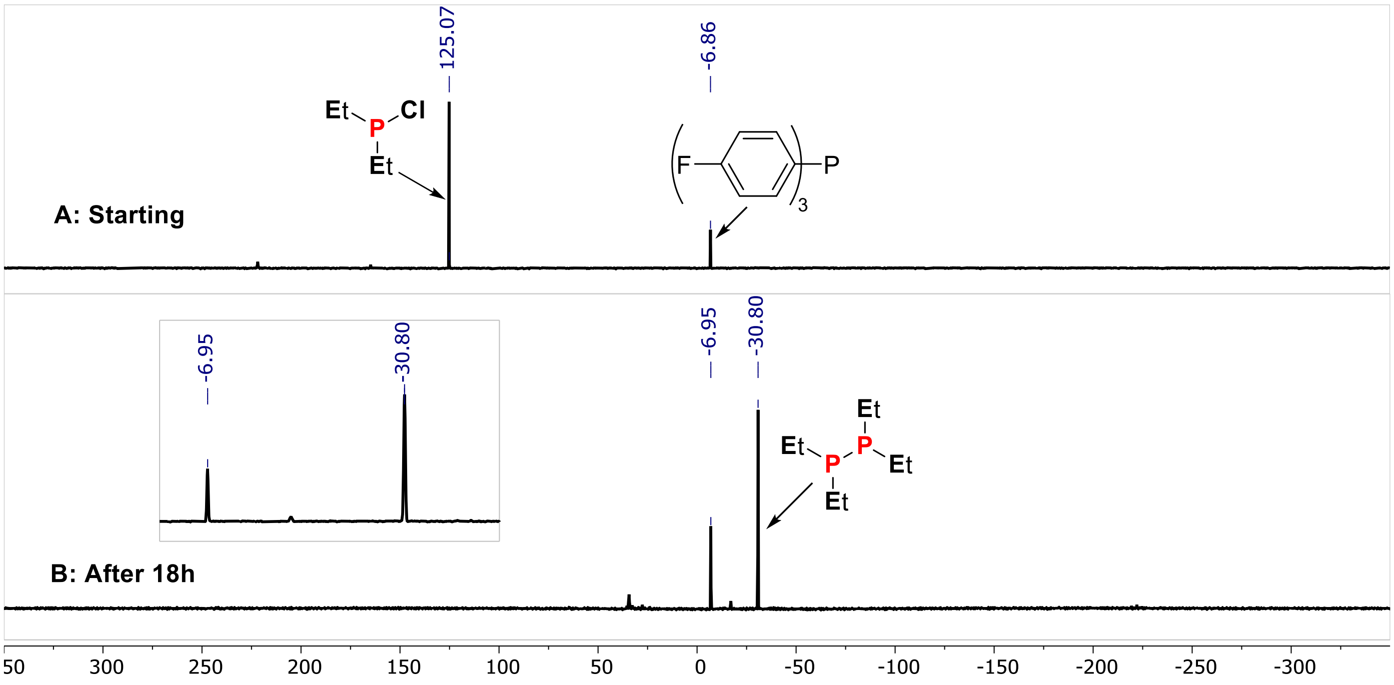

Figure S15: Crude ^{31}P NMR spectrum for **2b** using tris(4-fluorophenyl)phosphine as internal standard (Entry 2, Table S1).

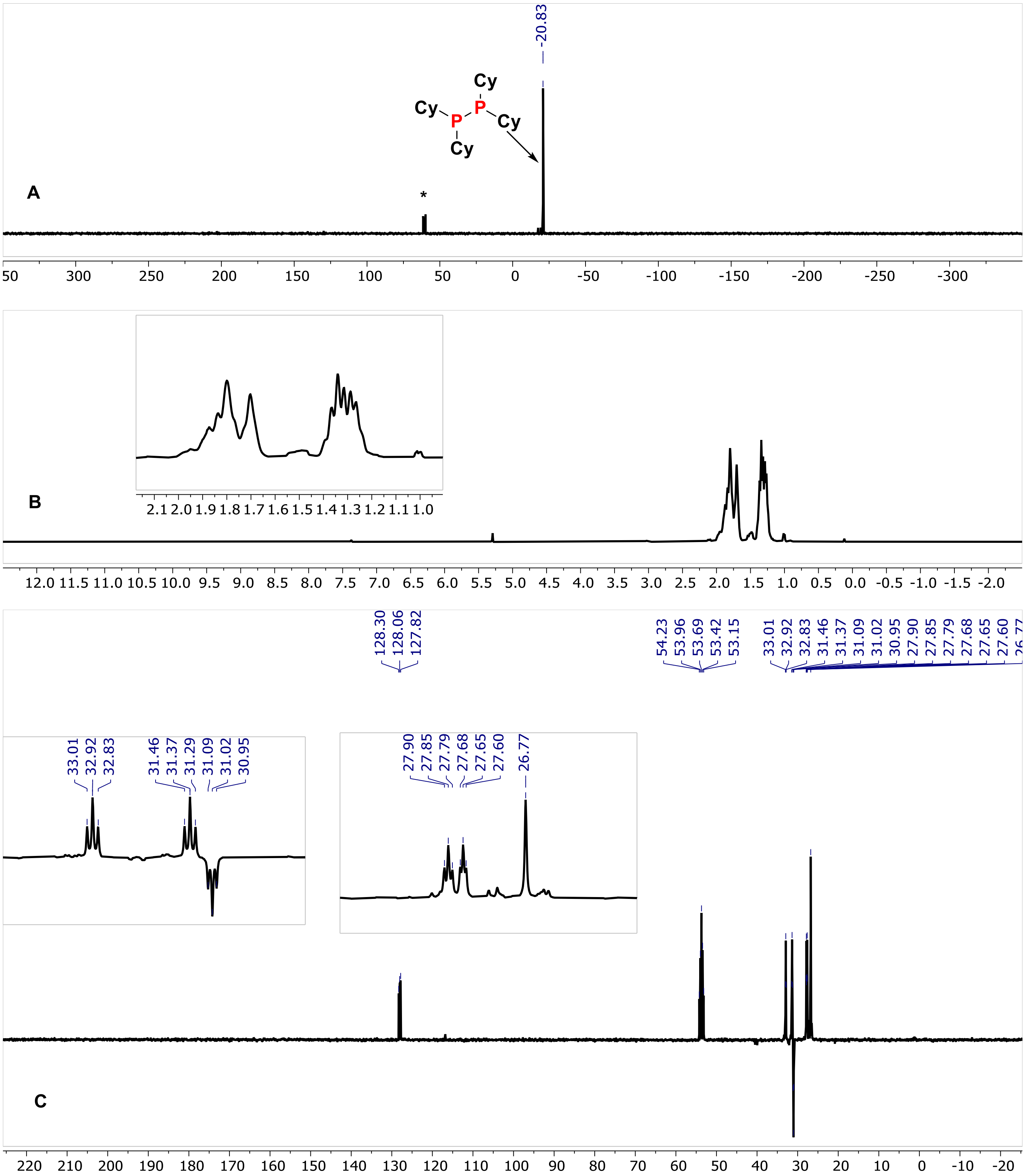

Figure S16: ^{31}P NMR (A), ^{1}H NMR (B) and ^{13}C NMR (C) spectrum of isolated **2c** (CD_2Cl_2/C_6D_6) (Entry 3, Table S1). * the signal is tentatively assigned to the HCl adduct of **2c**.

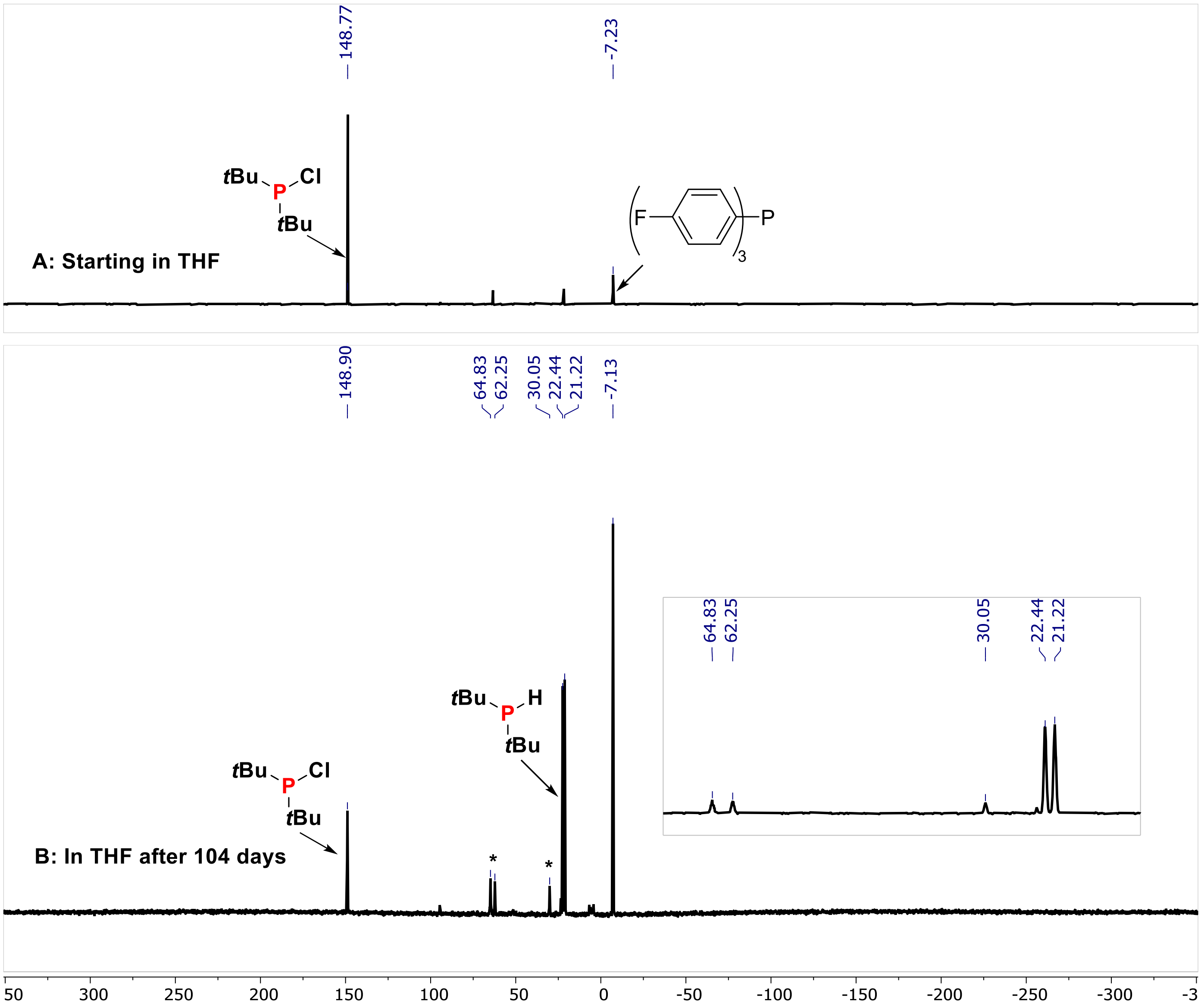

Figure S17: Crude ^{31}P NMR spectrum for **3d** using tris(4-fluorophenyl)phosphine as internal standard (Entry 4, Table S1). * marks the signal of an unknown by-product.

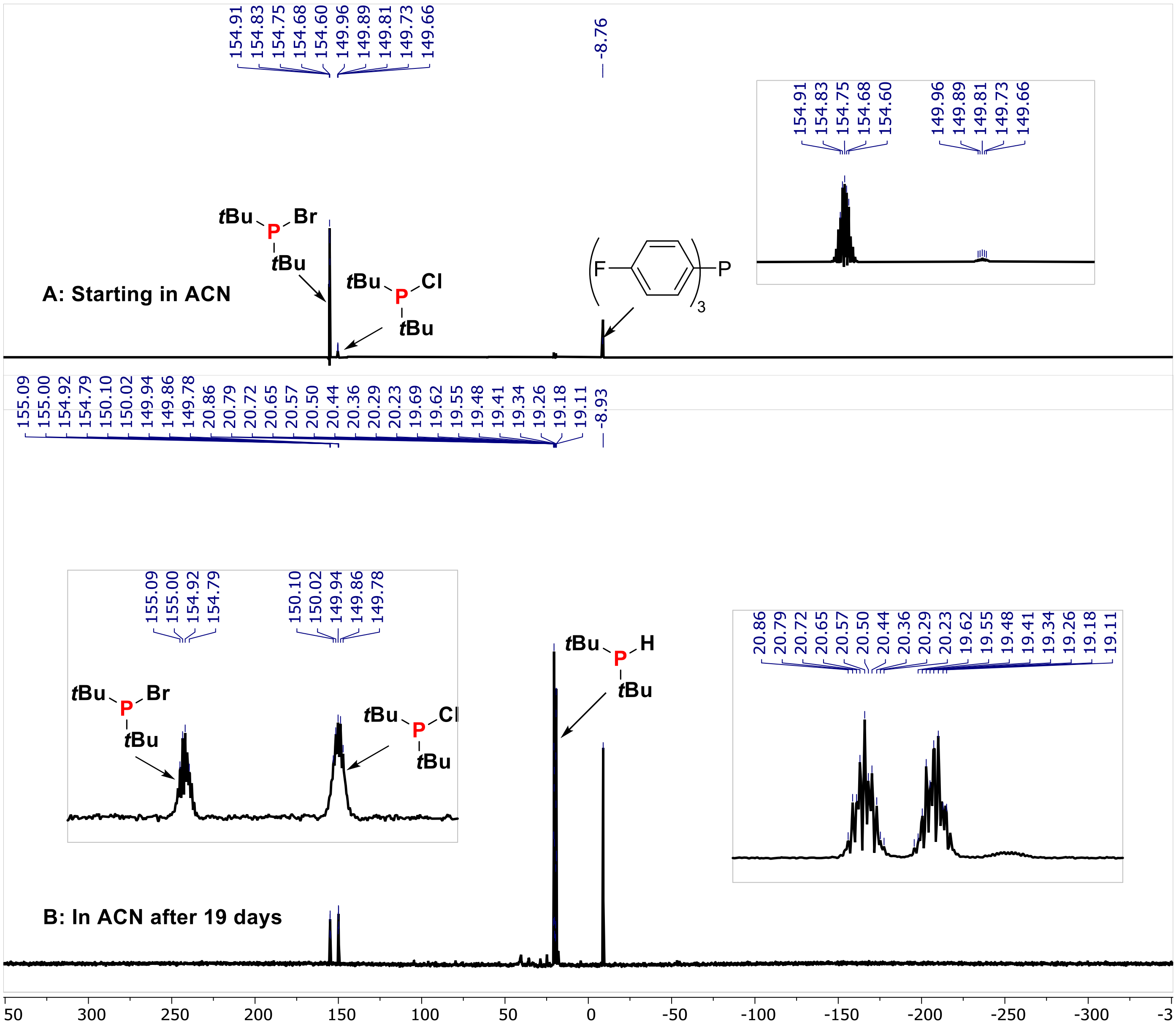

Figure S18: Crude ^{31}P NMR spectrum for **3d** using tris(4-fluorophenyl)phosphine as internal standard (Entry 5, Table S1).

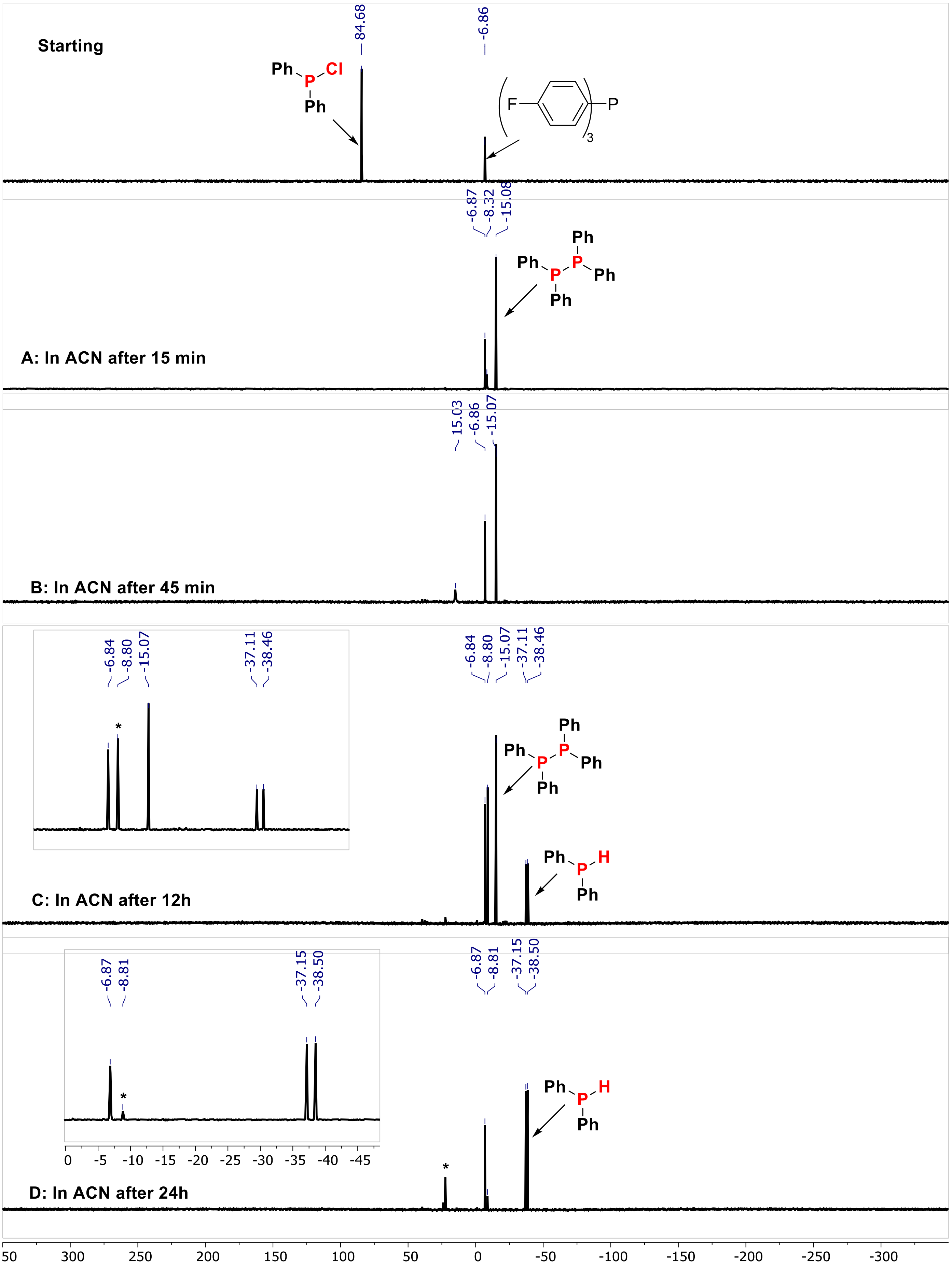

Figure S19: Crude ^{31}P NMR spectrum for **2e** and **3e** starting from Ph_2PCl in ACN using tris(4-fluorophenyl)phosphine as internal standard (Entry 6, Table S1).* marks the signal of an unknown by-product.

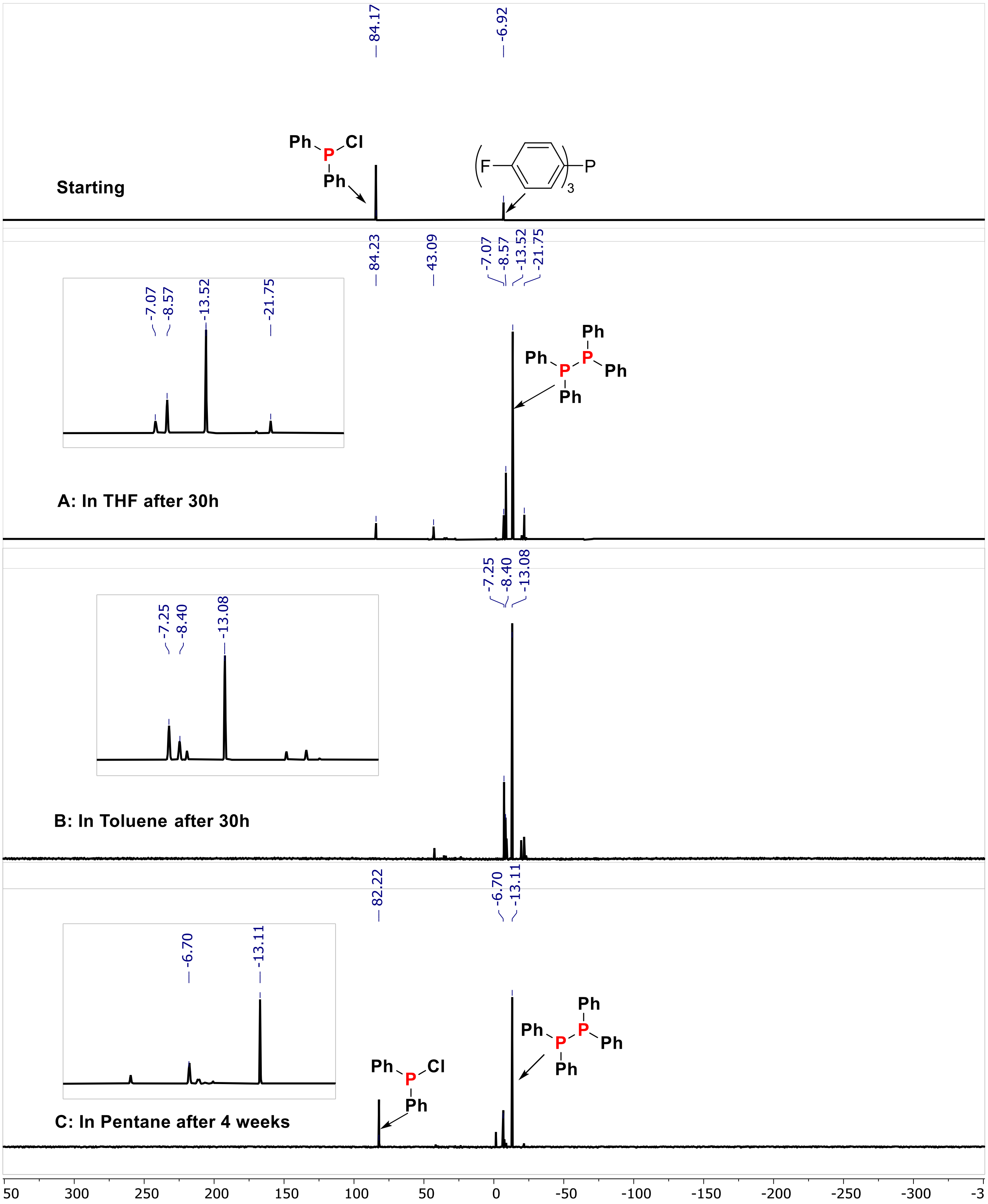

Figure S20: Crude ^{31}P NMR spectrum for **2e** starting from Ph_2PCl in different solvents using tris(4-fluorophenyl)phosphine as internal standard (Entry 7, 8, and 9, Table S1).

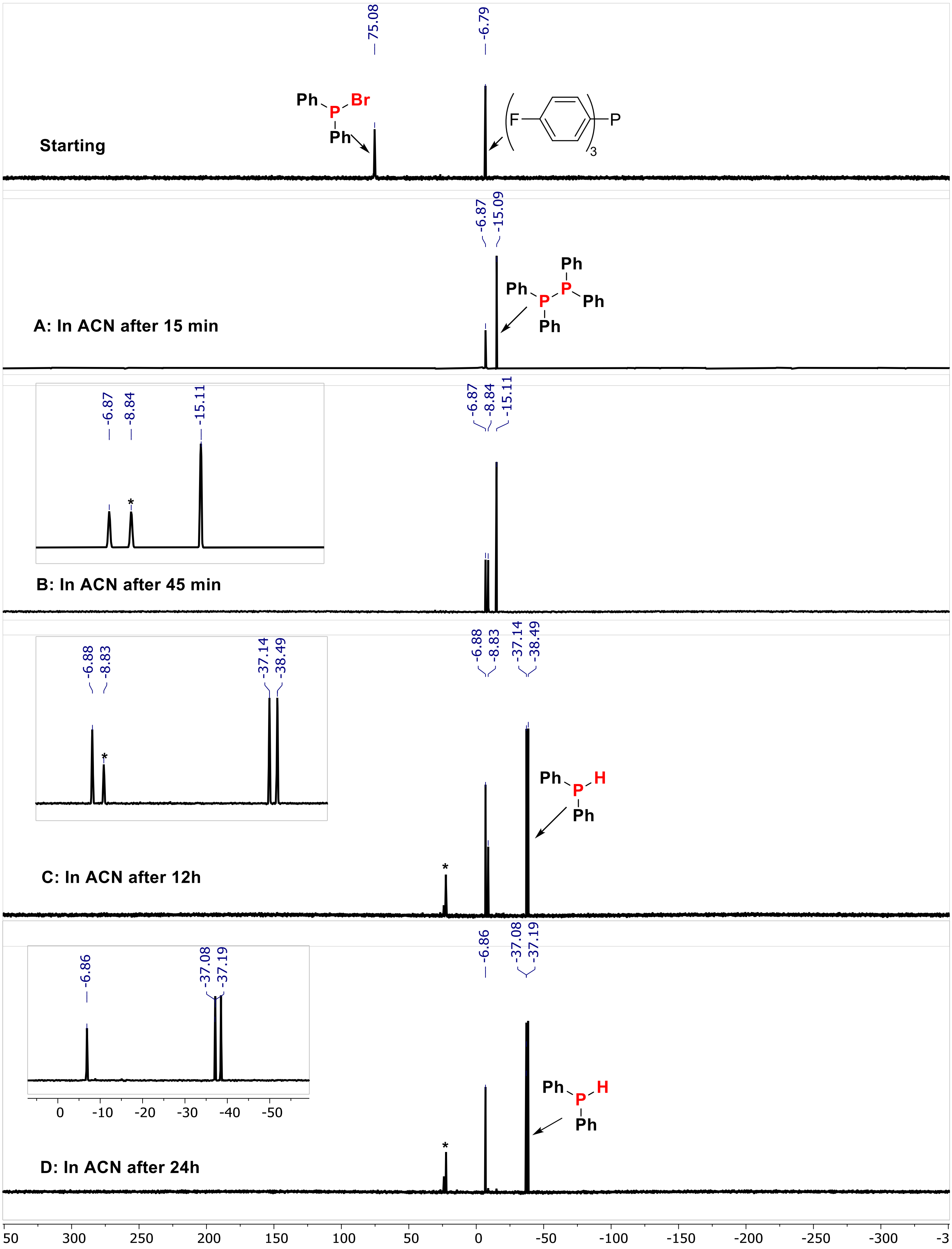

Figure S21*:* Crude ^{31}P NMR spectrum for **2e** and **3e** starting from Ph_2PBr using tris(4-fluorophenyl)phosphine as internal standard (Entry 10, Table S1). * marks the signal of an unknown by-product.

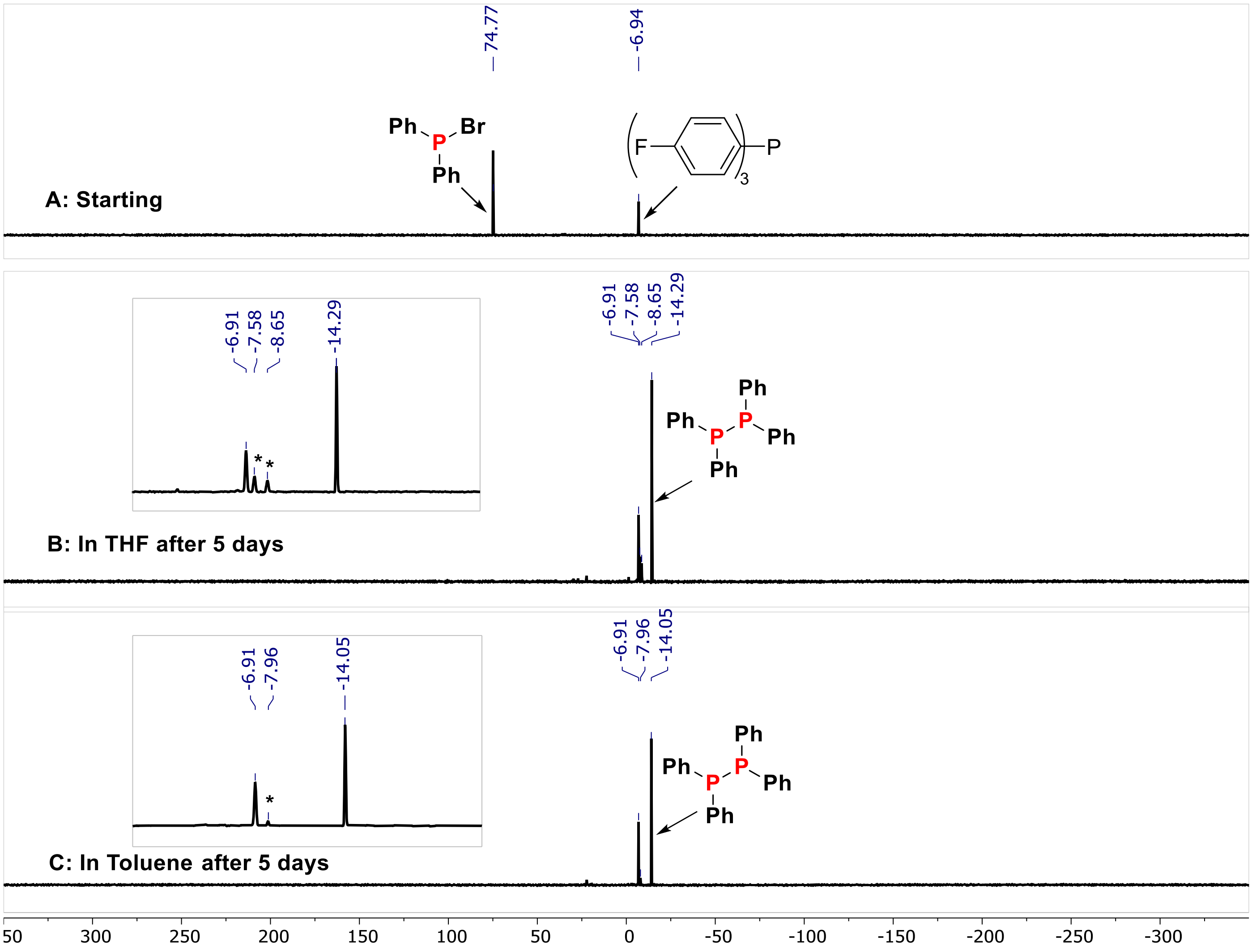

Figure S22: Crude ^{31}P NMR spectrum for **2e** starting from Ph_2PBr in different solvents using tris(4-fluorophenyl)phosphine as internal standard (Entry 11 and 12, Table S1). * marks the signal of an unknown by-product.

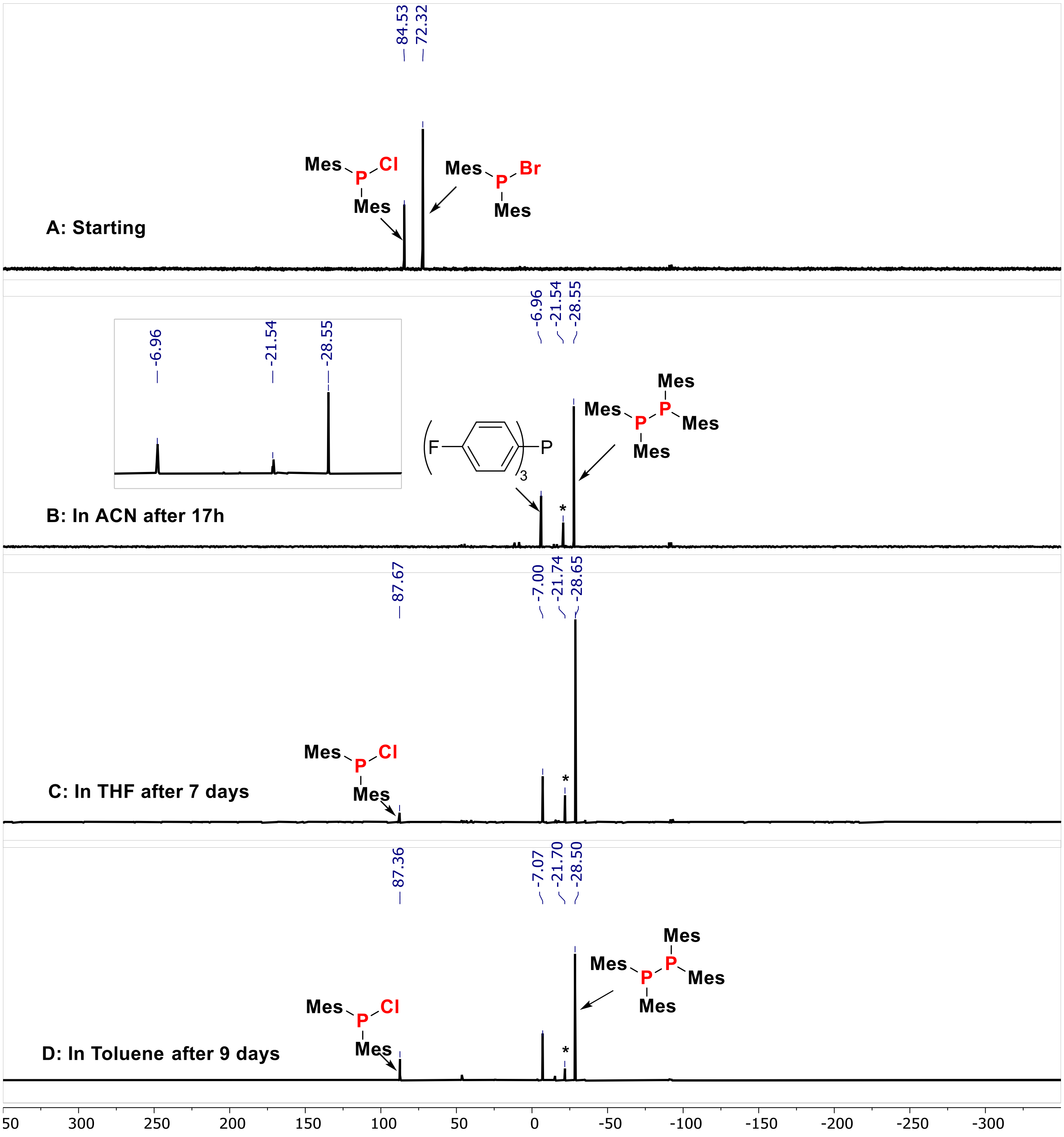

Figure S23: Crude ^{31}P NMR spectrum for **2f** starting from Mes_2PHal in different solvents using tris(4-fluorophenyl)phosphine as internal standard (Entry 13, 14 and 15, Table S1). * marks the signal of an unknown by-product.

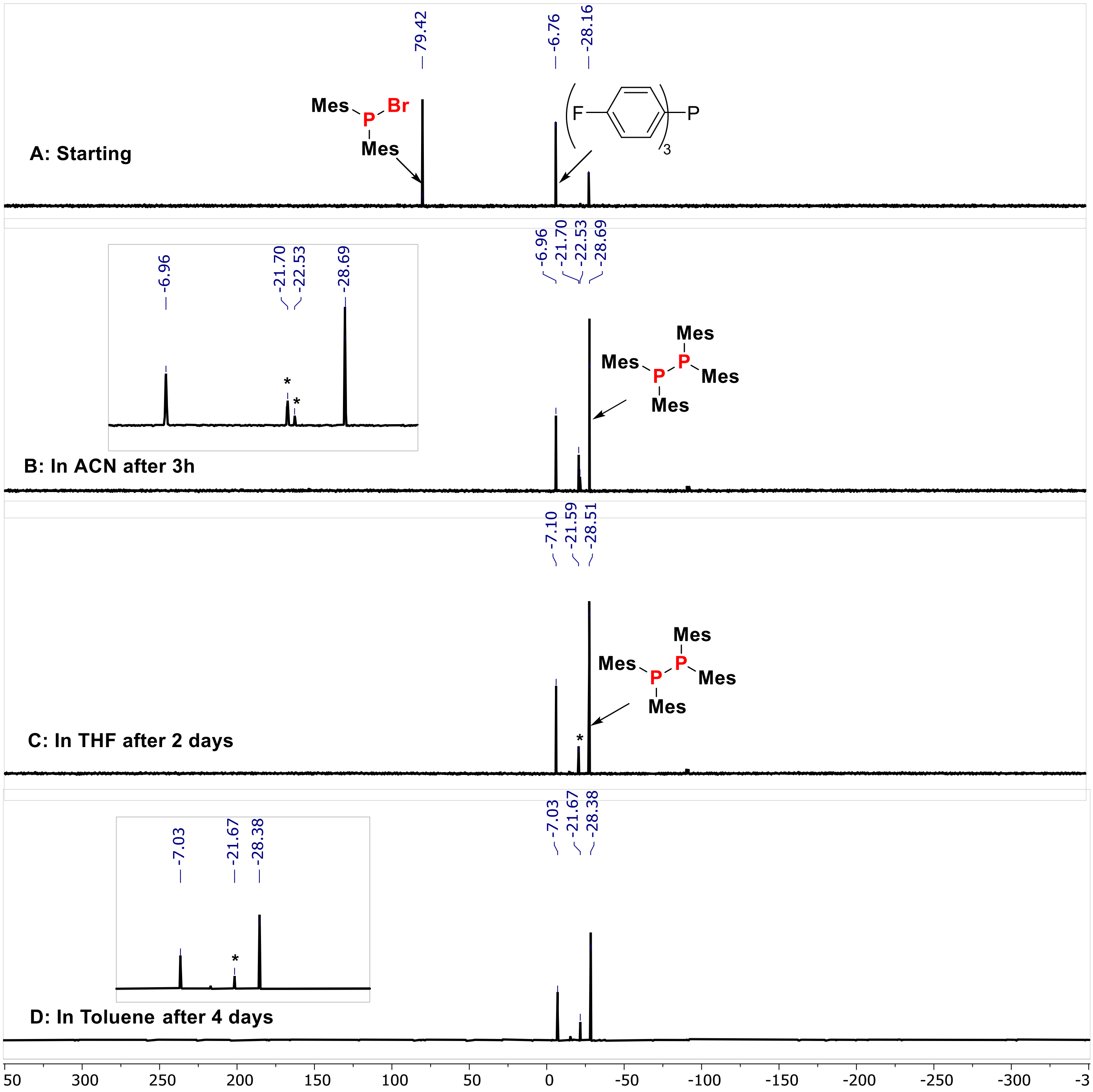

Figure S24: Crude ^{31}P NMR spectrum for **2f** starting from Mes_2PBr in different solvents using tris(4-fluorophenyl)phosphine as internal standard (Entry 16, 17 and 18, Table S1). * marks the signal of an unknown by-product.

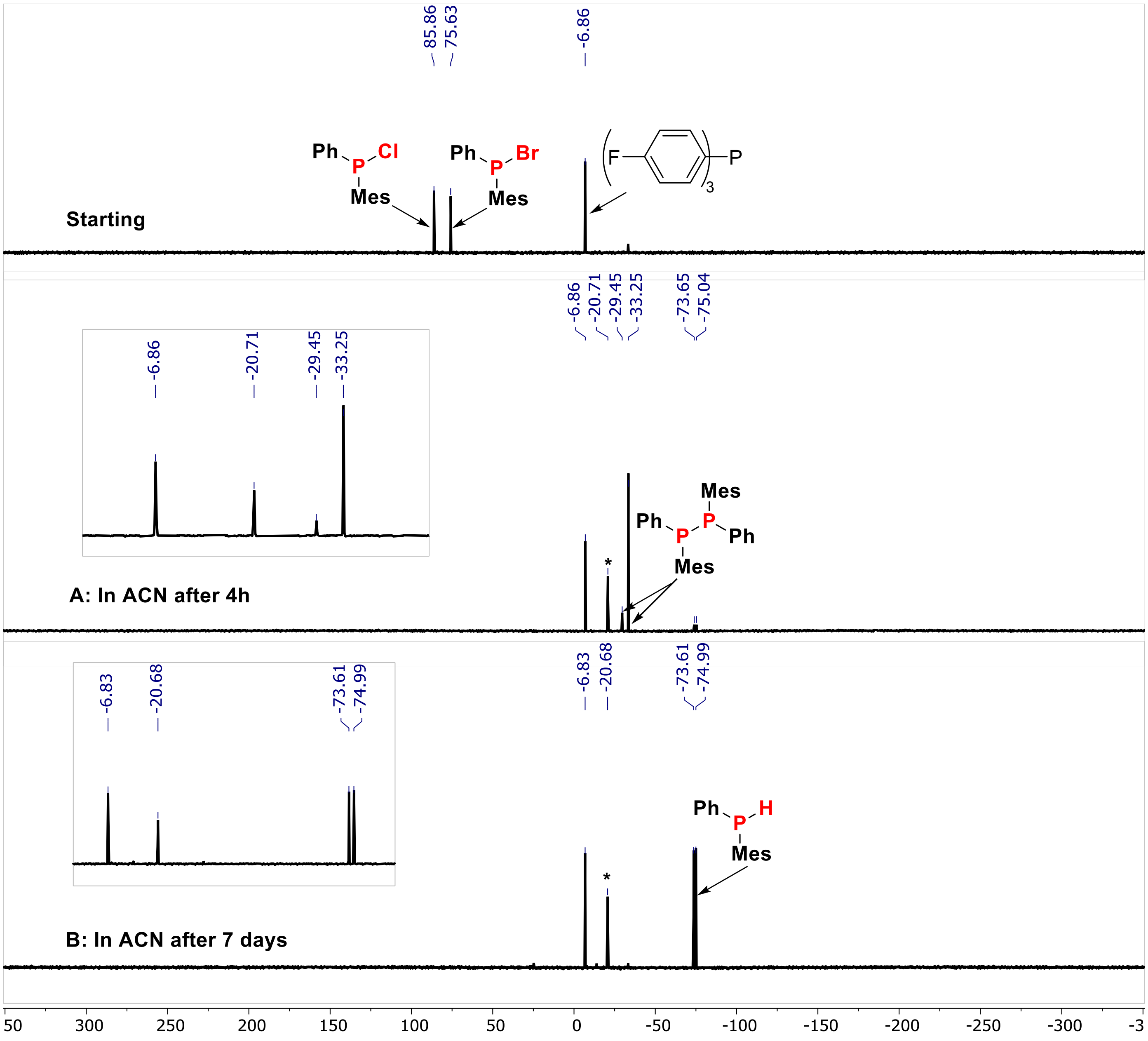

Figure S25: Crude ^{31}P NMR spectrum for **2g** and **3g** starting from MesPhPHal using tris(4-fluorophenyl)phosphine as internal standard (Entry 19, Table S1). * marks the signal of an unknown by-product.

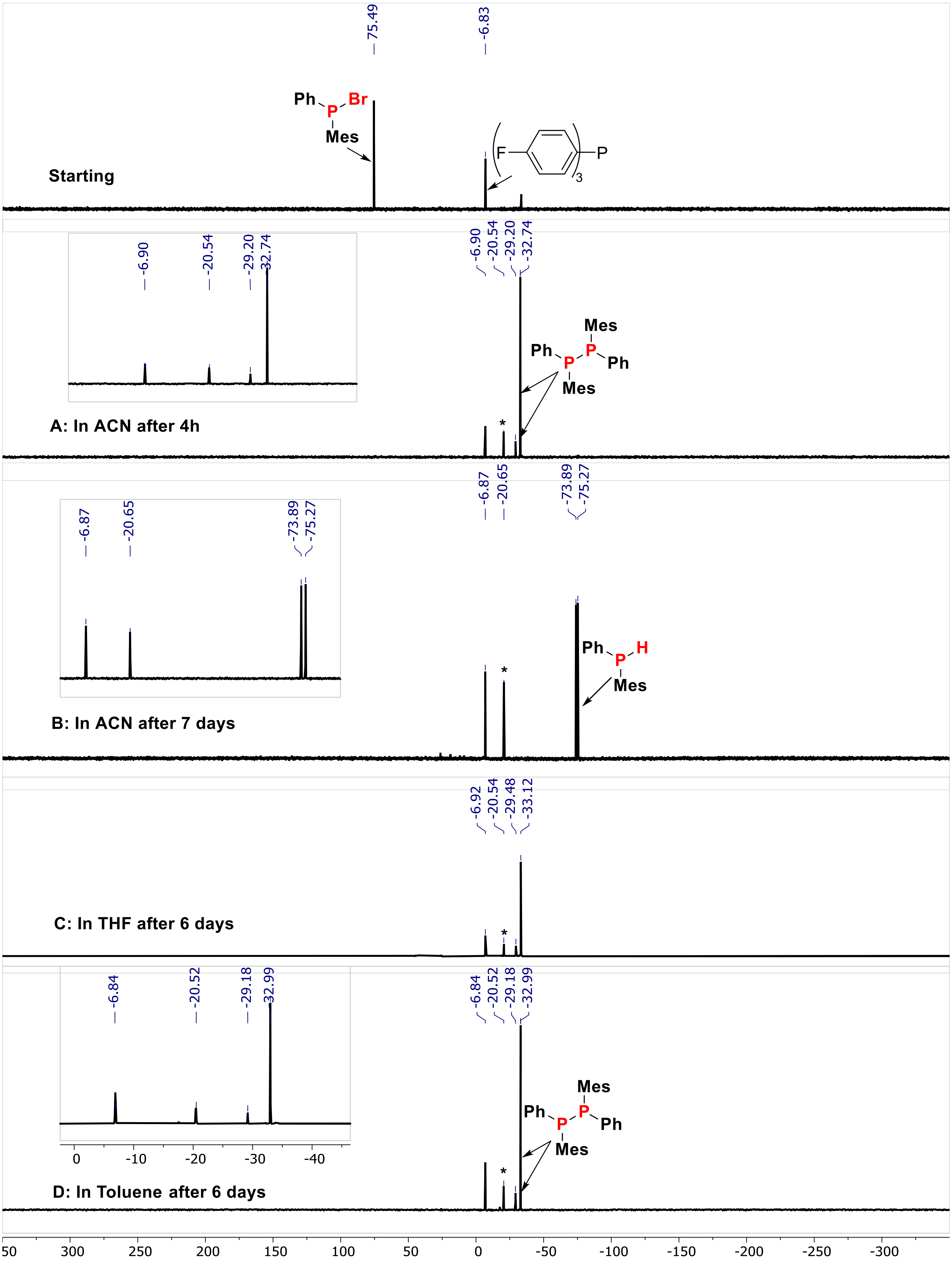

Figure S26: Crude ^{31}P NMR spectrum for **2g** and **3g** starting from MesPhPBr in different solvents using tris(4-fluorophenyl)phosphine as internal standard (Entry 20, 21 and 22, Table S1). * marks the signal of an unknown by-product.

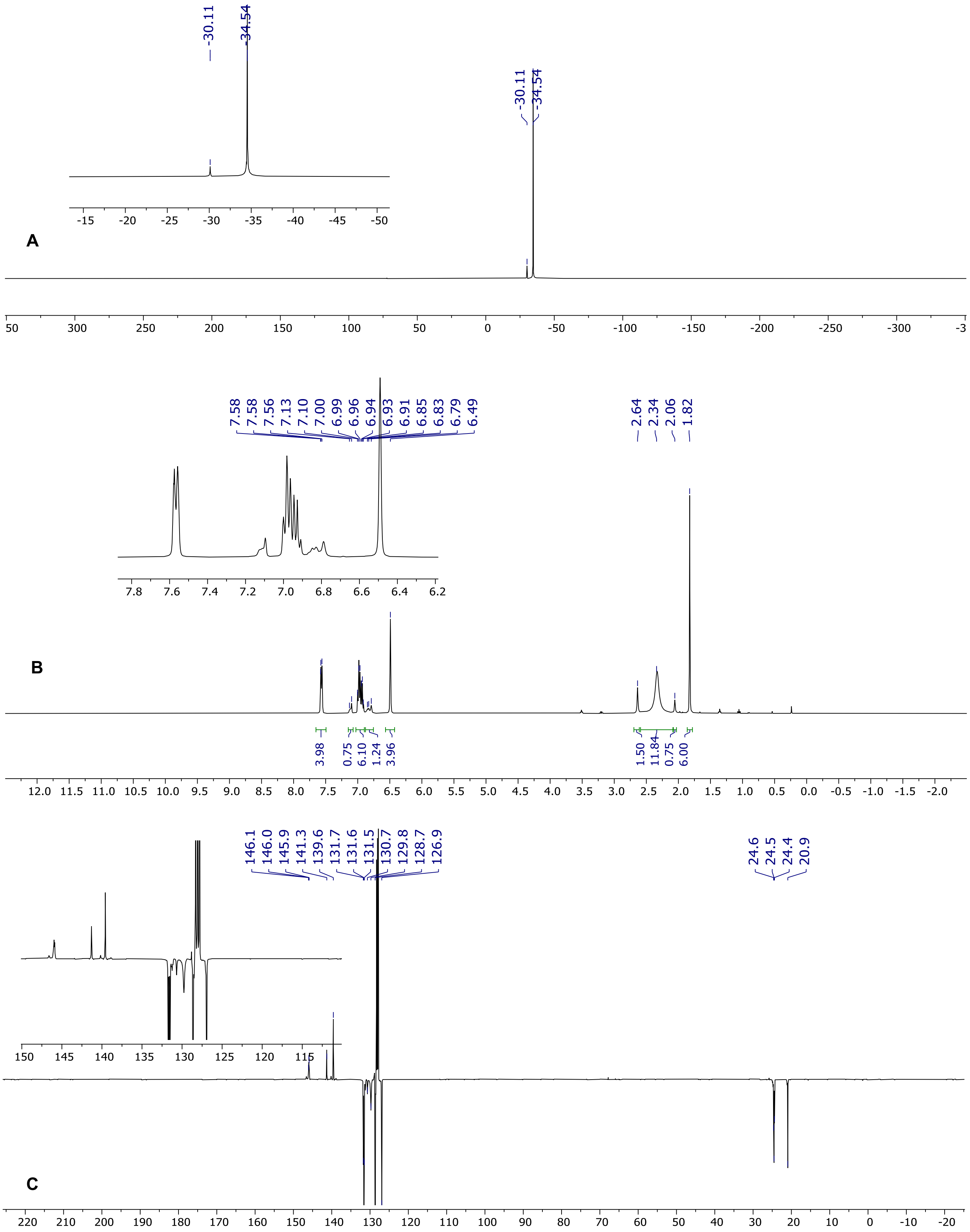

Figure S27: ^{31}P NMR (A), ^{1}H NMR (B) and ^{13}C NMR (C) spectrum of isolated **2g** (C_6D_6) (Entry 20, Table S1).

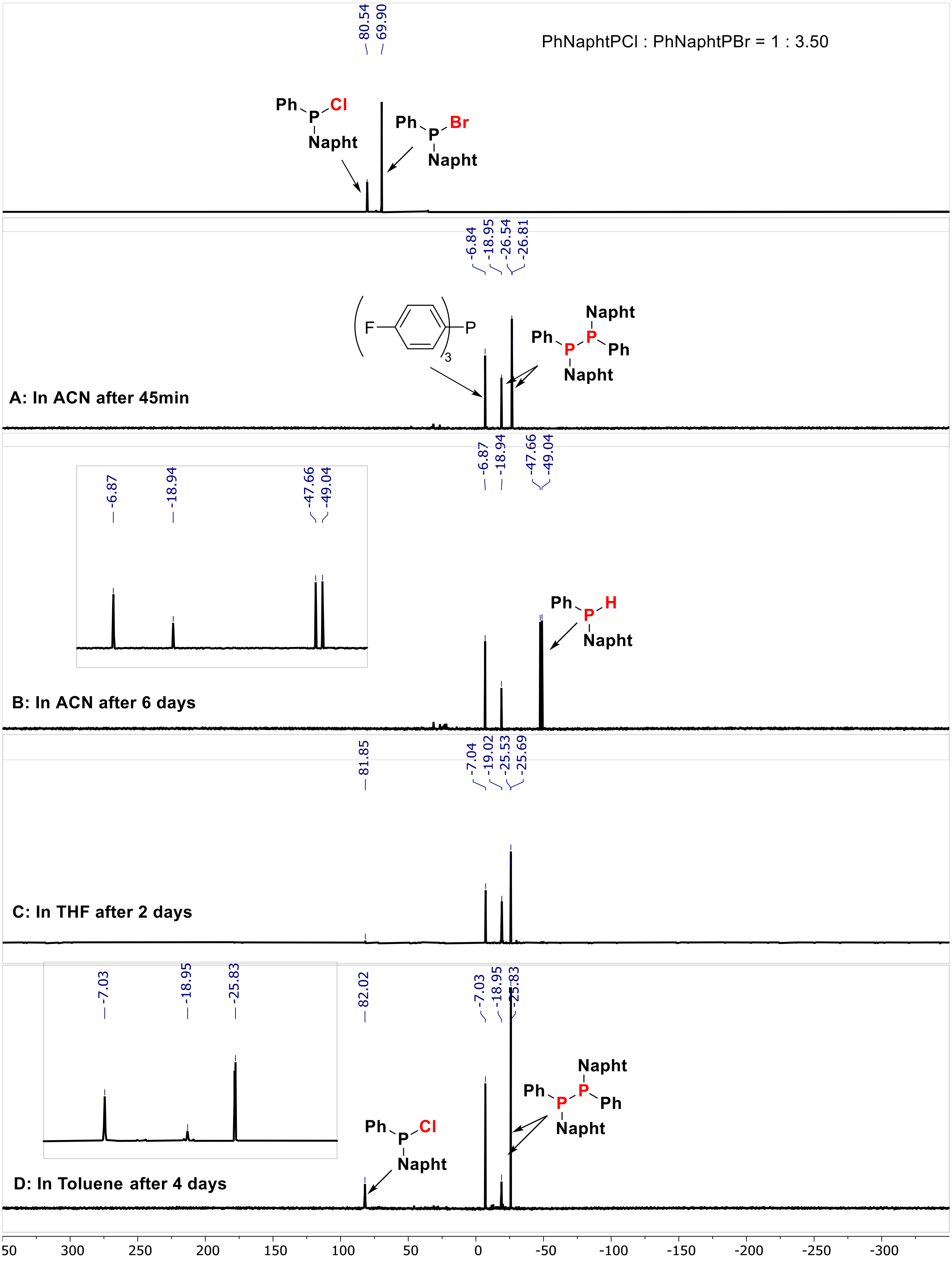

Figure S28: Crude ^{31}P NMR spectrum for **2h** and **3h** starting from PhNaphtPHal in different solvents using tris(4-fluorophenyl)phosphine as internal standard (Entry 23, 24 and 25, Table S1).

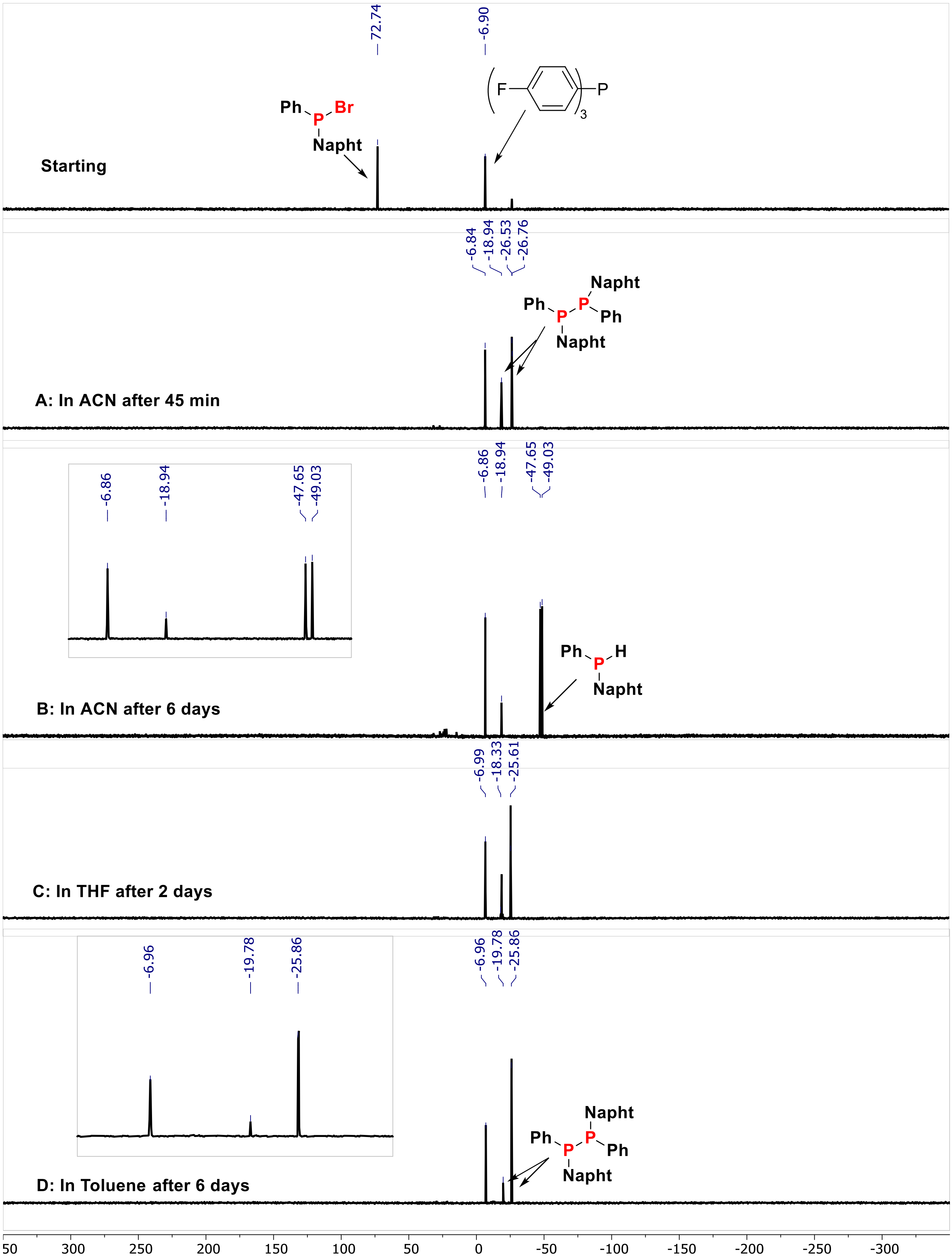

Figure S29: Crude ^{31}P NMR spectrum for **2h** and **3h** starting from PhNaphtPBr in different solvents using tris(4-fluorophenyl)phosphine as internal standard (Entry 26, 27 and 28, Table S1).

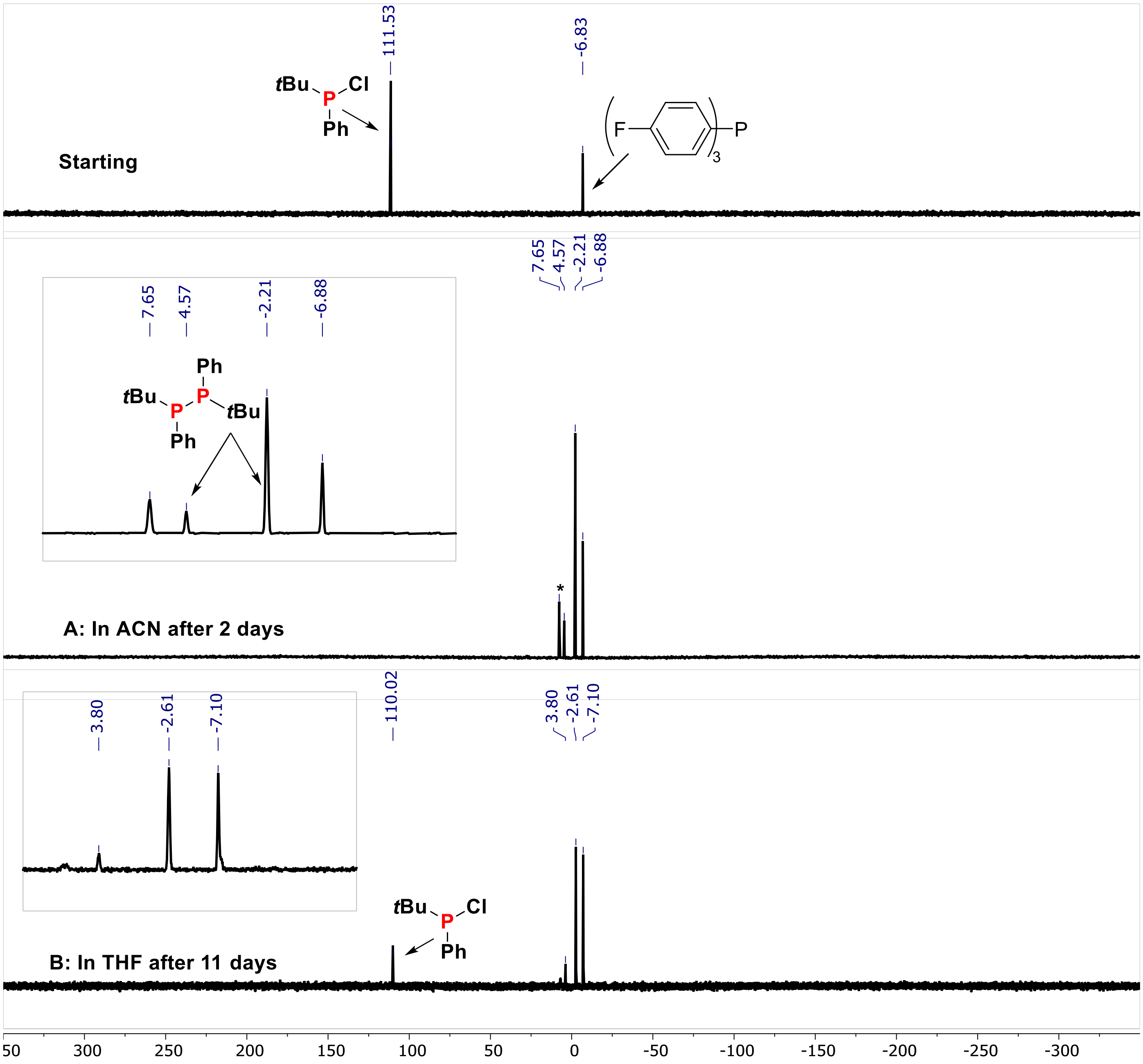

Figure S30: Crude ^{31}P NMR spectrum for **2i** starting from tBuPhPCl using tris(4-fluorophenyl)phosphine as internal standard (Entry 29 and 30, Table S1). * marks the signal of an unknown by-product.

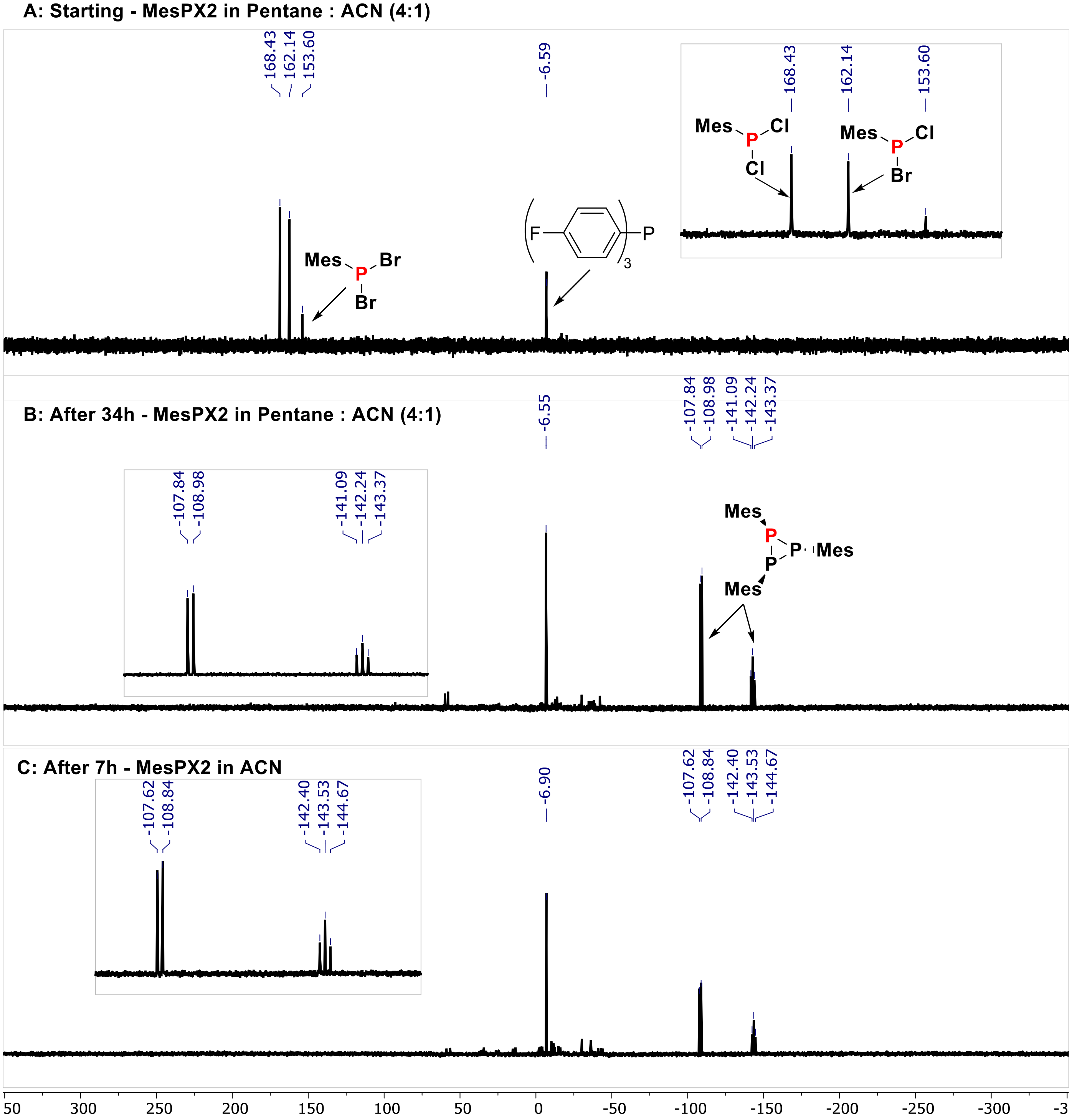

Figure S31: Crude ^{31}P NMR spectrum for **5j** starting from $MesPX_2$ in different solvents using tris(4-fluorophenyl)phosphine as internal standard (Entry 1 and 2, Table S2).

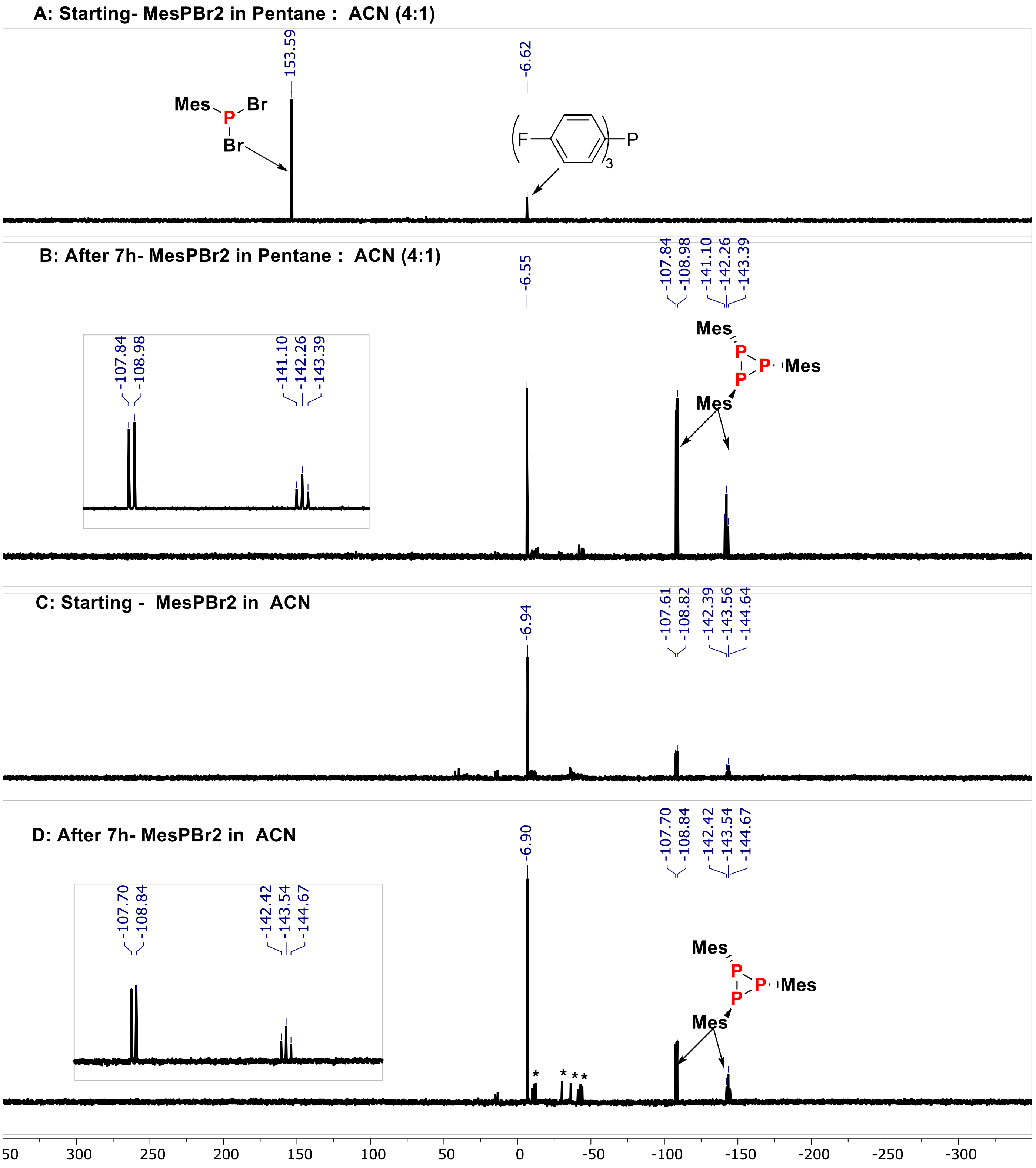

Figure S32: Crude ^{31}P NMR spectrum for **5j** starting from $MesPBr_2$ in different solvents using tris(4-fluorophenyl)phosphine as internal standard (Entry 3 and 4, Table S2). * marks the signal of an unknown by-product.

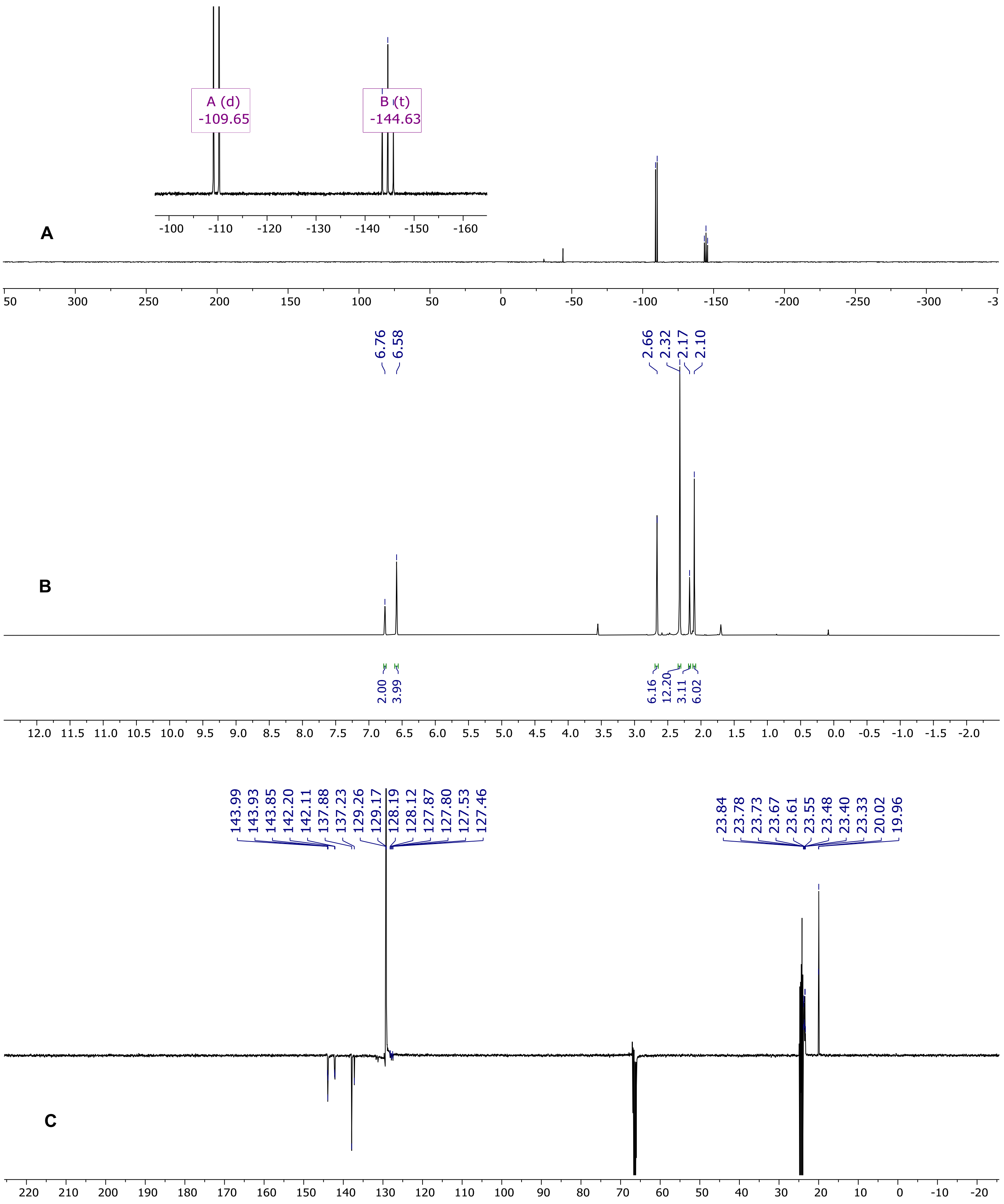

Figure S33: ^{31}P NMR (A), ^{1}H NMR (B) and ^{13}C NMR (C) spectrum of isolated **5j** (THF-D_8) (Entry 3, Table S2).

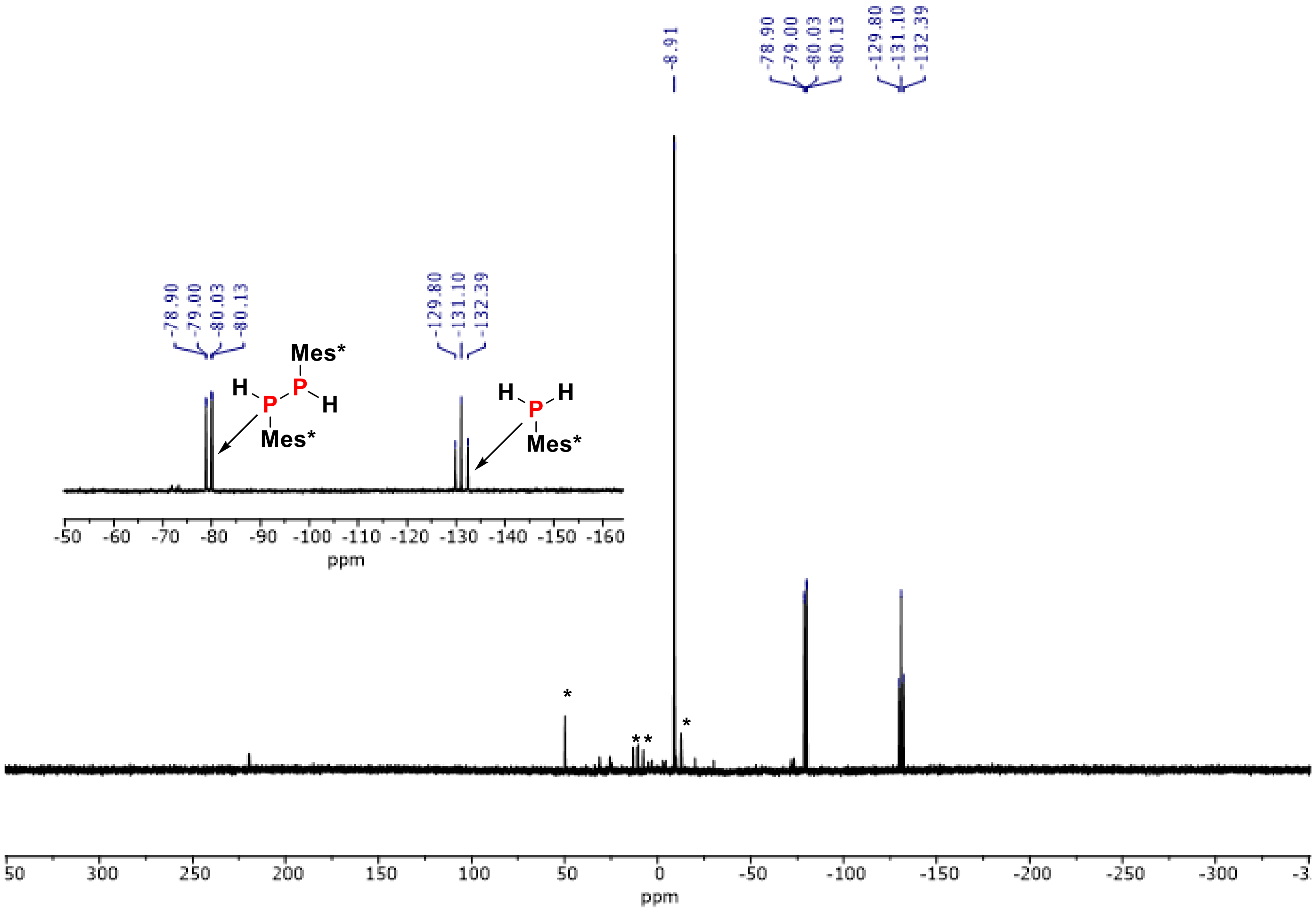

Figure S34: Crude ^{31}P NMR spectrum for starting from Mes*PCl_2 in ACN-d3 using tris(4-fluorophenyl)phosphine as internal standard (Entry 5, Table S2). * marks the signal of an unknown by-product.

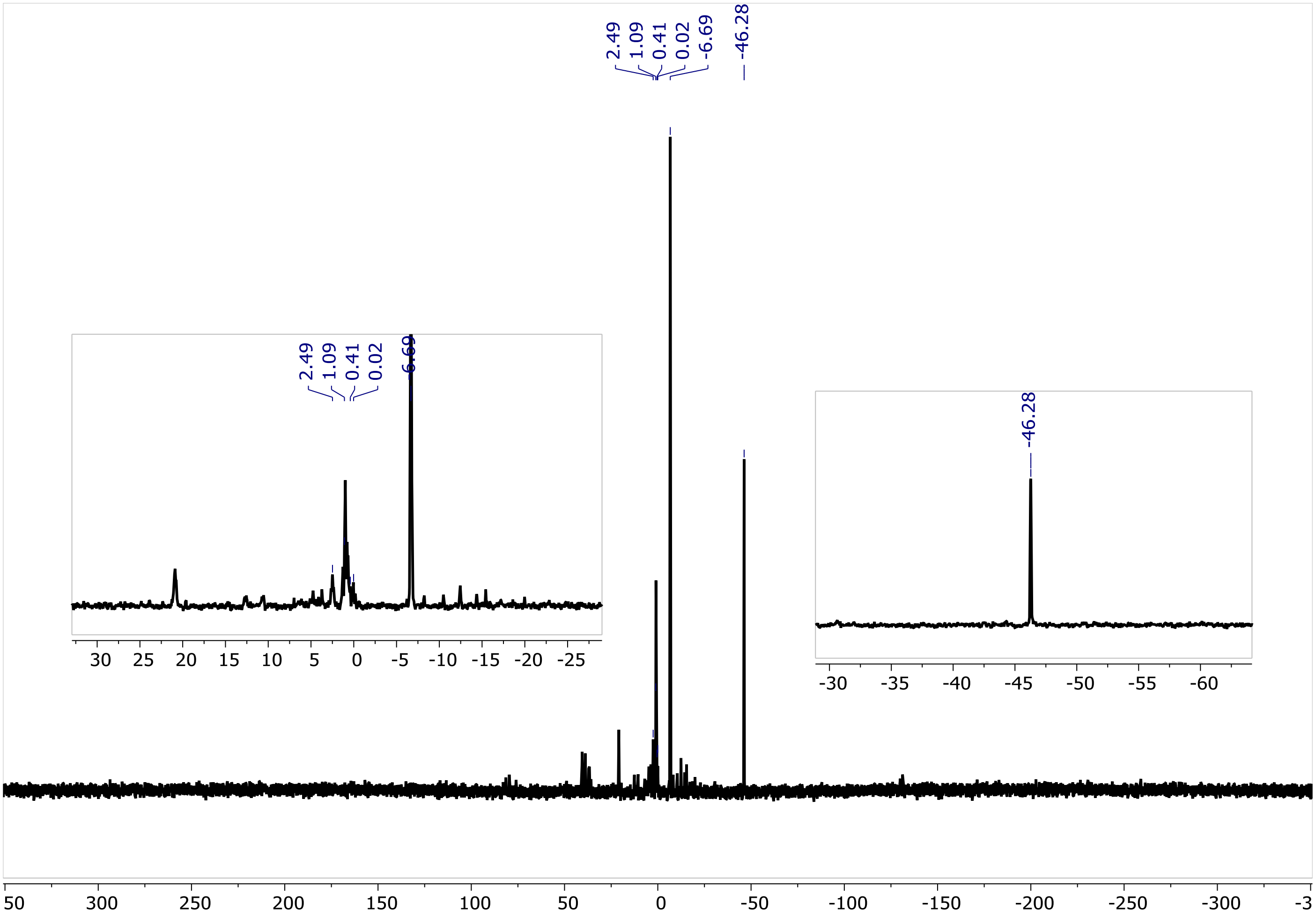

Figure S35: ^{31}P NMR spectrum for Iridium-Catalyzed photoreaction of $PhPBr_2$ using tris(4-fluorophenyl)phosphine as internal standard (Entry 6, Table S2).

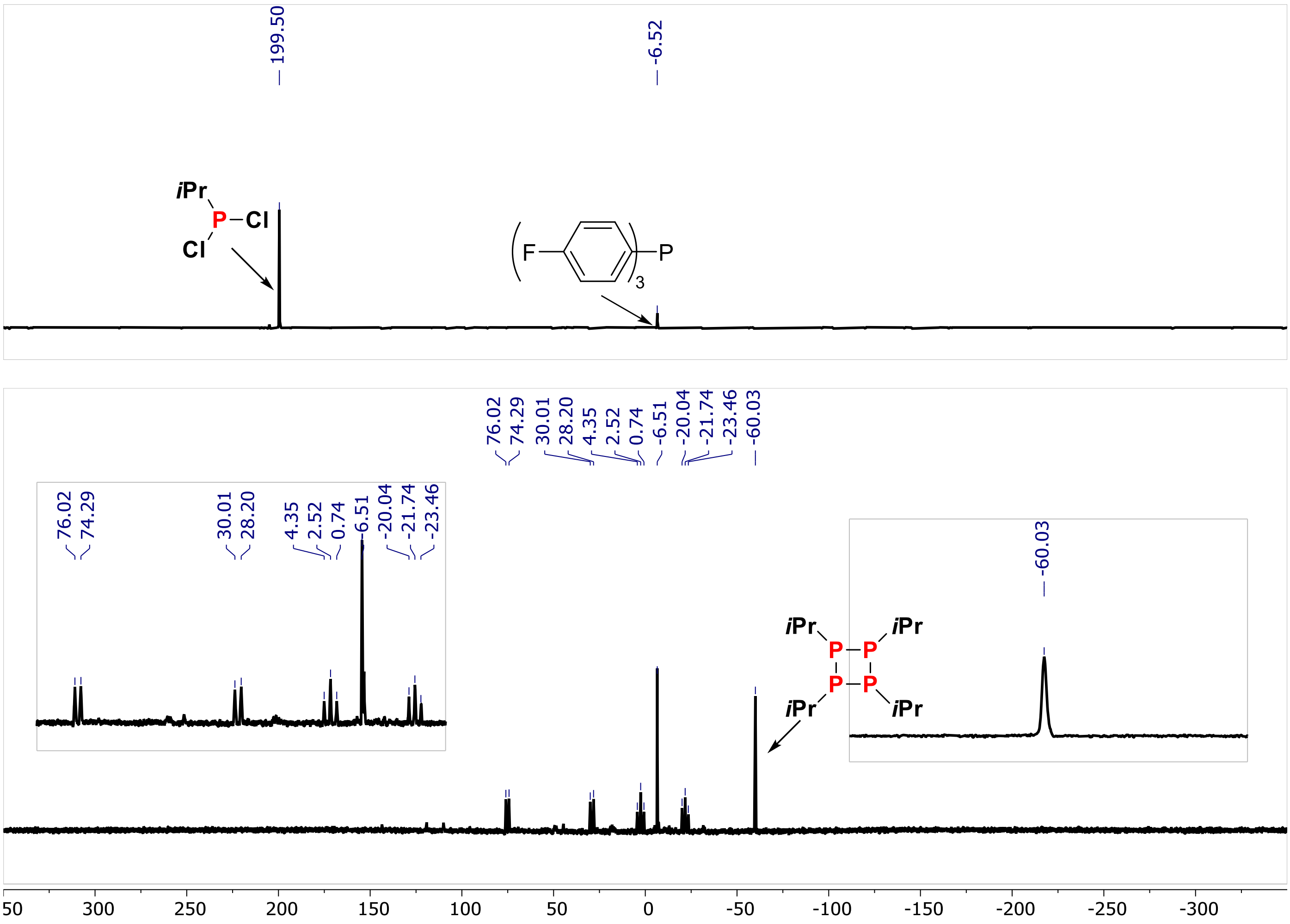

Figure S36: ^{31}P NMR spectrum for Iridium-catalyzed photoreaction of *i*PrPCl_2 using tris(4-fluorophenyl)phosphine as internal standard (Entry 7, Table S2). The ^{31}P NMR spectrum shows four additional signals that are tentatively assigned to a linear version of a P4 species. This compound is the major product, obtained in ca. 70 % crude yield.

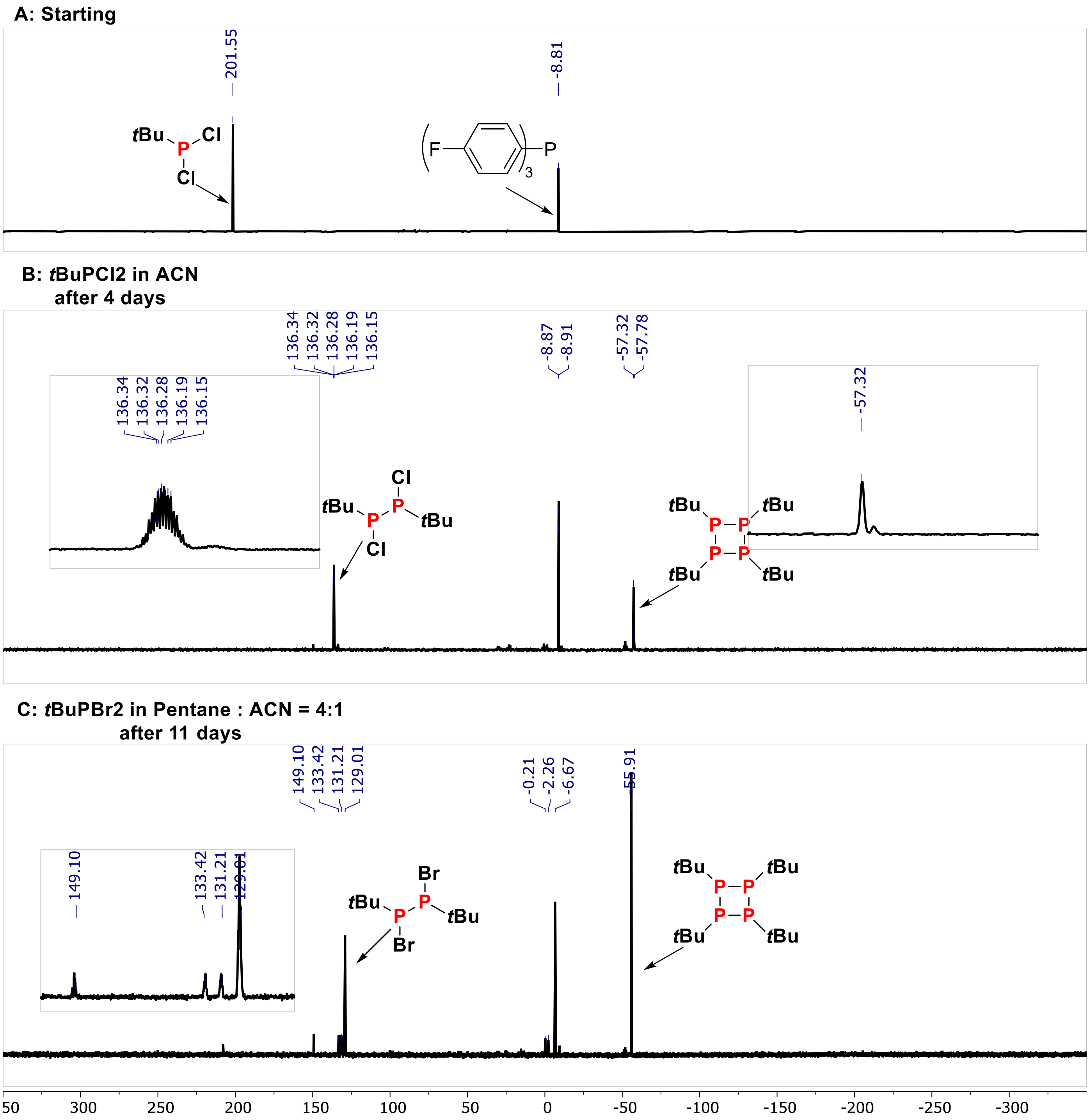

Figure S37: ^{31}P NMR spectrum for Iridium-catalyzed photoreaction of *t*BuPCl$_2$ and *t*BuPBr$_2$ using tris(4-fluorophenyl)phosphine as internal standard (Entry 8 and 9, Table S2).

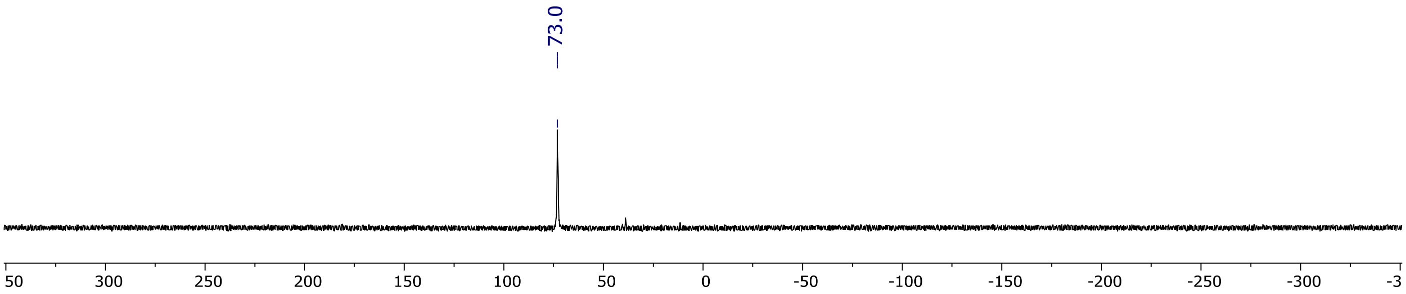

Figure S38: ^{31}P NMR (C_6D_6) spectrum of Ph_2PBr

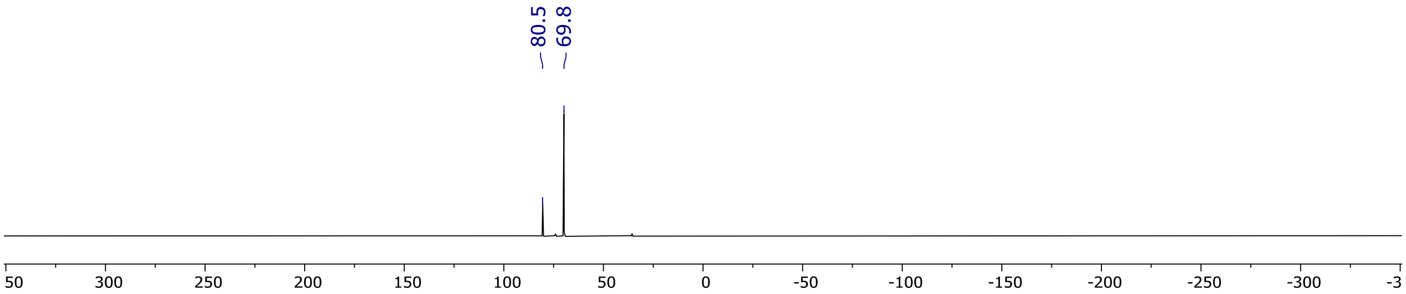

Figure S39: ^{31}P NMR (C_6D_6) spectrum of PhNaphtPX

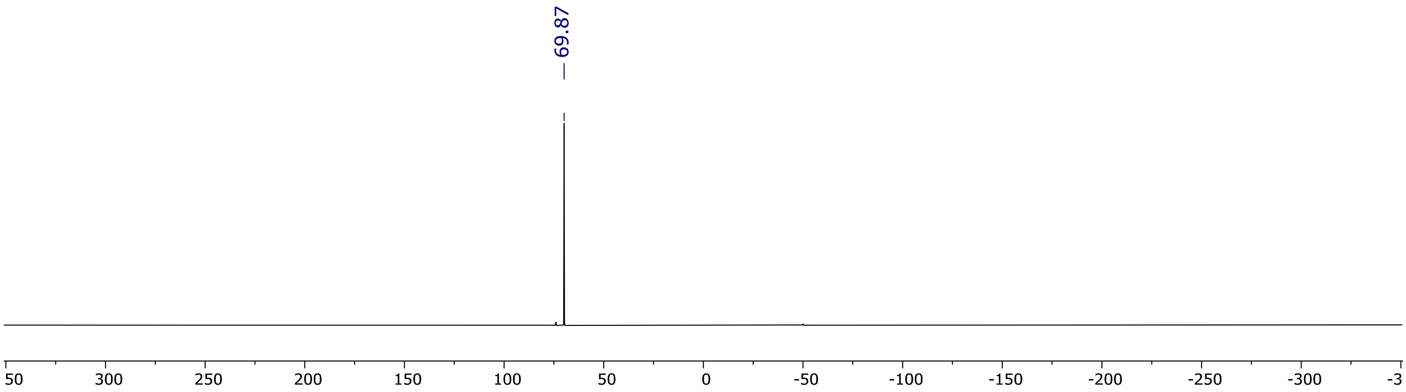

Figure S40: ^{31}P NMR (C_6D_6) spectrum of PhNaphtPBr

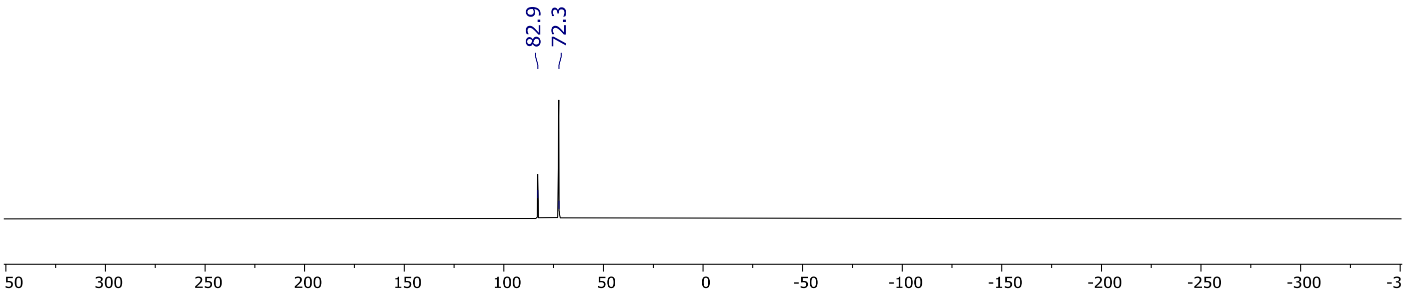

Figure S41: ^{31}P NMR (C_6D_6) spectrum of PhMesPX

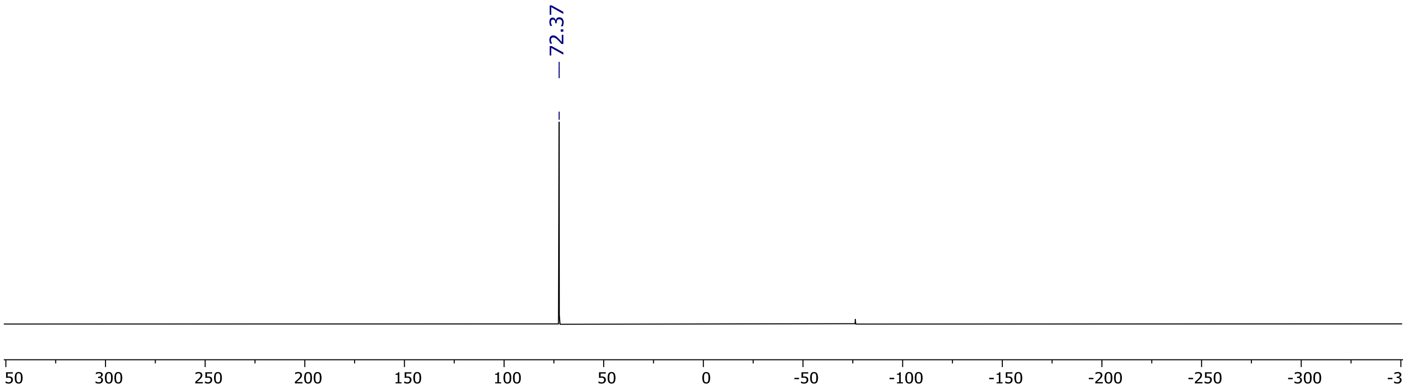

Figure S42: ^{31}P NMR (C_6D_6) spectrum of PhMesPBr

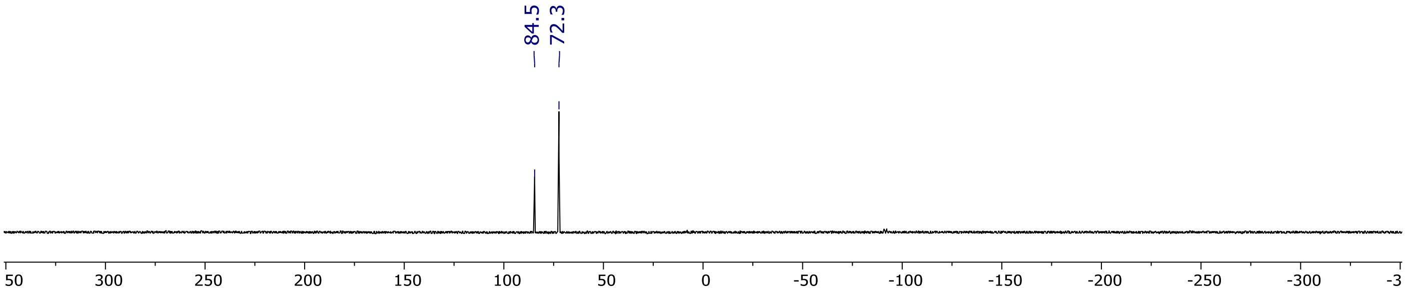

Figure S43: ^{31}P NMR (C_6D_6) spectrum of Mes_2PX

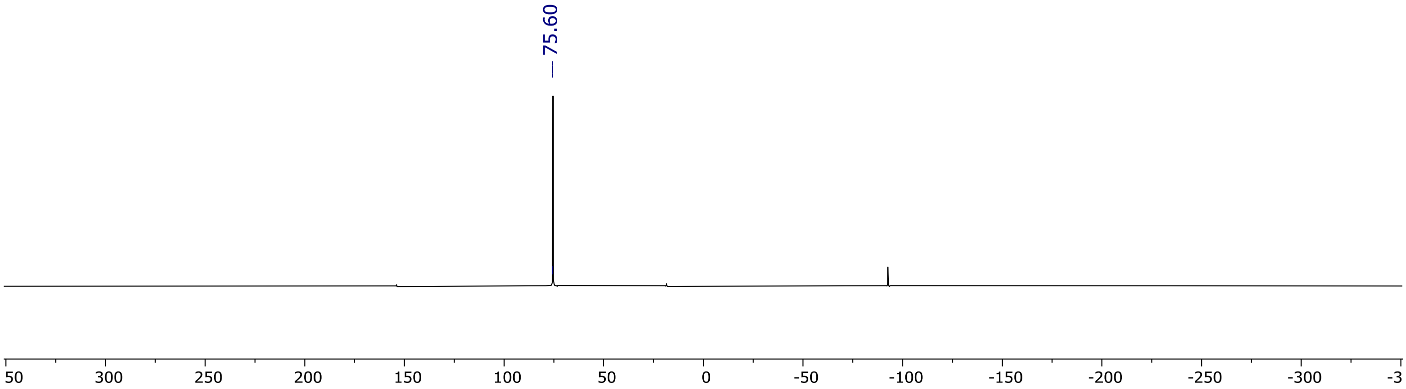

Figure S44: ^{31}P NMR (C_6D_6) spectrum of Mes_2PBr

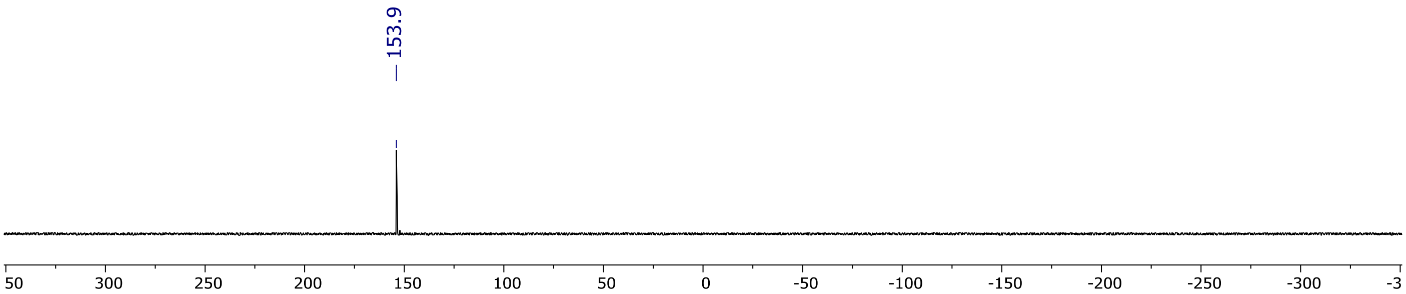

Figure S45: ^{31}P NMR spectrum of Mes^*PCl_2

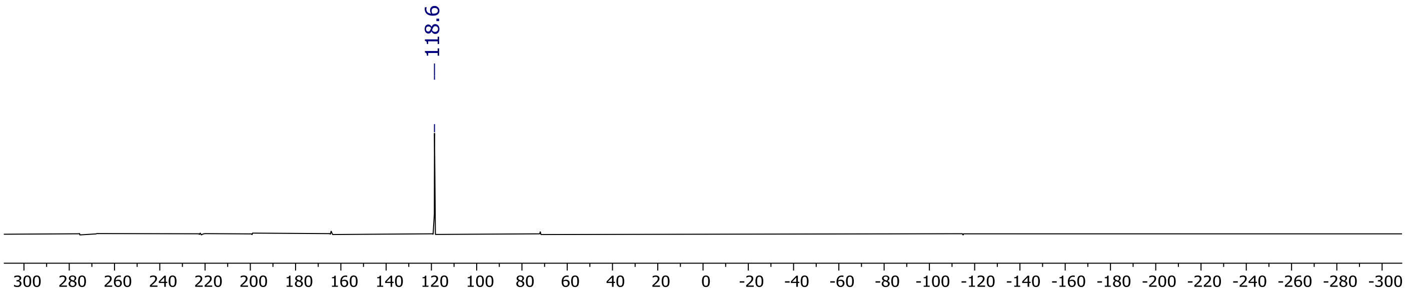

Figure S46: ^{31}P NMR (C_6D_6) spectrum of Et_2PCl

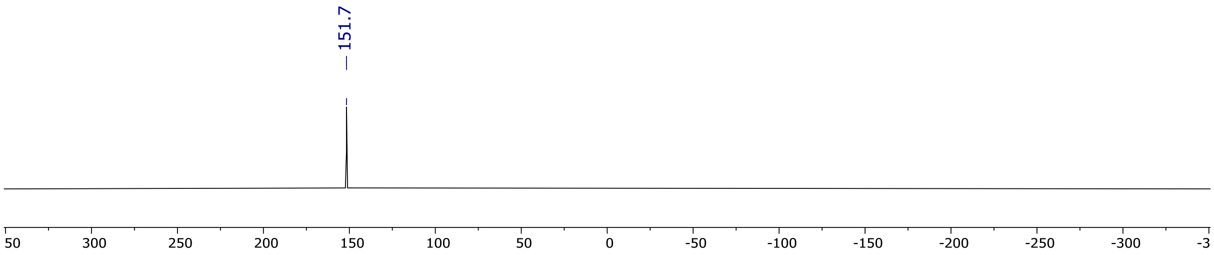

Figure S47: ^{31}P NMR (ACN-D_3) spectrum of *t*Bu_2PBr.

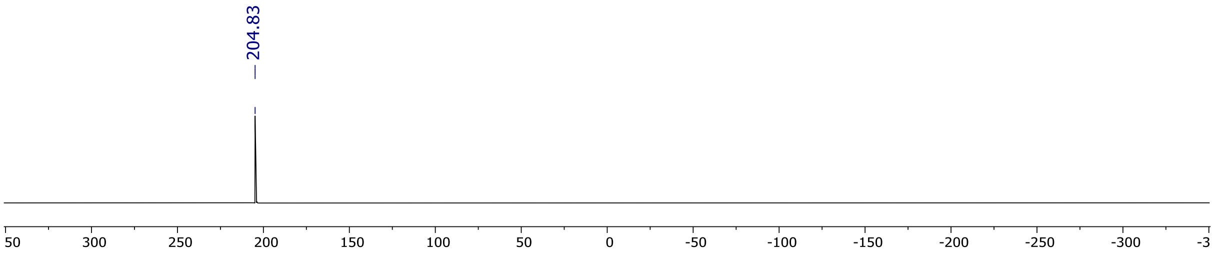

Figure S48: ^{31}P NMR (ACN-D_3) spectrum of *t*$BuPBr_2$.

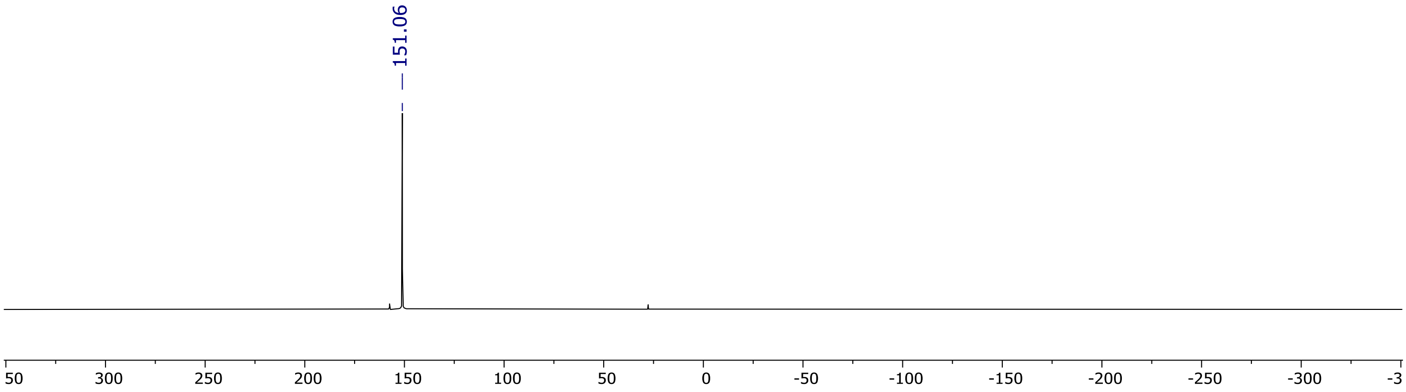

Figure S49: ^{31}P NMR (C_6D_6) spectrum of $PhPBr_2$.

7. References

[1] F. Brunner, A. Babaei, A. Pertegás, J. M. Junquera-Hernández, A. Prescimone, E. C. Constable, H. J. Bolink, M. Sessolo, E. Ortí, C. E. Housecroft, *Dalton Trans.* **2019**, *48*, 446-460.

[2] K. Dziuba, A. Flis, A. Szmigielska, K. M. Pietrusiewicz, *Tetrahedron: Asymmetry* **2010**, *21*, 1401-1405.

[3] J.-E. Siewert, A. Schumann, C. Hering-Junghans, *Dalton Trans.* **2021**, *50*, 15111-15117.

[4] D. J. Dellinger, D. M. Sheehan, N. K. Christensen, J. G. Lindberg, M. H. Caruthers, *J. Am. Chem. Soc.* **2003**, *125*, 940-950.

[5] I. Bonnaventure, A. B. Charette, *J. Org. Chem.* **2008**, *73*, 6330-6340.

[6] A. H. Cowley, N. C. Norman, M. Pakulski, G. Becker, M. Layh, E. Kirchner, M. Schmidt, *Inorg. Synth*, **1990**, *27*, 235-240.

[7] L. Liu, H.-C. Wu, J.-Q. Yu, *Chem. Eur. J*, **2011**, *17*, 10828-10831.

[8] S. Humbel, C. Bertrand, C. Darcel, C. Bauduin, S. Jugé, *Inorg. Chem*, **2003**, *42*, 420-427.

[9] A. Hinke, W. Kuchen, *Phosphorus Sulfur Relat. Elem*, **1983**, *15*, 93-98.

[10] P. Kutzer, S. Hartung, O. Pirali, R. Pietschnig, C. Medcraft, K. M. T. Yamada, T. F. Giesen, *Heteroat. Chem* **2017**, *28*, 21361.

[11] A. Schumann, F. Reiß, H. Jiao, J. Rabeah, J.-E. Siewert, I. Krummenacher, H. Braunschweig, C. Hering-Junghans, *Chem. Sci*, **2019**, *10*, 7859-7867.

[12] S. Aime, R. K. Harris, E. M. McVicker, M. Fild, *J. Chem. Soc., Dalton Trans*, **1976**, *21*, 2144-2153.

[13] D. L. Dodds, M. F. Haddow, A. G. Orpen, P. G. Pringle, G. Woodward, *Organometallics*, **2006**, *25*, 5937-5945.

[14] E. Baum, E. Matern, J. Pikies, A. Robaszkiewicz, *Z. Anorg. Allg. Chem.*, **2004**, *630*, 1090-1095.

[15] G. Baccolini, C. Boga, M. Mazzacurati, F. Sangirardi, *Org. Lett*, **2006**, *8*, 1677-1680.

[16] S. Yasui, T. Ando, M. Ozaki, Y. Ogawa, K. Shioji, *Heteroat. Chem*, **2018**, *29*, e21468.

[17] J. Albert, R. Bosque, J. M. Cadena, S. Delgado, J. Granell, G. Muller, J. I. Ordinas, M. Font Bardia, X. Solans, *Chem. Eur. J.*, **2002**, *8*, 2279-2287.

[18] Y. Huang, Y. Li, P.-H. Leung, T. Hayashi, *J. Am. Chem. Soc.*, **2014**, *136*, 4865-4868.

[19] K. G. Pearce, A. M. Borys, E. R. Clark, H. J. Shepherd, *Inorg. Chem.*, **2018**, *57*, 11530-11536.

[20] J. Heinicke, R. Kadyrov, *J. Organomet. Chem.*, **1996**, *520*, 131-137.

[21] K. B. Dillon, V. C. Gibson, L. J. Sequeira, *J. Chem. Soc., Chem. Commun.*, **1995**, *23*, 2429-2430, B. M. Cossairt, C. C. Cummins, *New J. Chem*., **2010**, *34*, 1533-1536, A. Schumann, F. Reiß, H. Jiao, J. Rabeah, J.E. Siewert, I. Krummenacher, H. Braunschweig, C.H. Junghas. *Chem. Sci*., **2019**,*10*, 7859-7867.

[22] M. Yoshifuji, K. Shibayama, N. Inamoto, T. Matsushita, K. Nishimoto, *J. Am. Chem. Soc.*, **1983**, *105*, 2495-2497.

[23] A. H. Cowley, J. E. Kilduff, T. H. Newman, M. Pakulski, *J. Am. Chem. Soc.*, **1982**, *104*, 5820-5821.

[24] M. Baudler, B. Carlsohn, B. Kloth, D. Koch, *Z. Anorg. Allg. Chem.*, **1977**, *432*, 67-78.

[25] M. Baudler, G. Reuschenbach, *Z. Anorg. Allg. Chem.*, **1980**, *464*, 9-16.

[26] L. Wu, V. T. Annibale, H. Jiao, A. Brookfield, D. Collison, I. Manners, *Nat. Commun.*, **2019**, *10*, 2786.

[27] W. A. Henderson, M. Epstein, F. S. Seichter, *J. Am. Chem. Soc.*, **1963**, *85*, 2462-2466.

[28] Y. Mei, Z. Yan, L. L. Liu, *J. Am. Chem. Soc.*, **2022**, *144*, 1517-1522.
